# ROCK1 promotes B cell differentiation and proteostasis under stress through the heme-regulated proteins, BACH2 and HRI

**DOI:** 10.1172/jci.insight.180507

**Published:** 2025-02-04

**Authors:** Juan Rivera-Correa, Sanjay Gupta, Edd Ricker, Danny Flores-Castro, Daniel Jenkins, Stephen Vulcano, Swati P. Phalke, Tania Pannellini, Matthew M. Miele, Zhuoning Li, Nahuel Zamponi, Young-Bum Kim, Yurii Chinenov, Eugenia Giannopoulou, Leandro Cerchietti, Alessandra B. Pernis

**Affiliations:** 1 Autoimmunity and Inflammation Program, Hospital for Special Surgery, New York, New York, USA.; 2 Biological Sciences Department, New York City College of Technology, City University of New York, Brooklyn, New York, USA.; 3 Research Division and Precision Medicine Laboratory, Hospital for Special Surgery, New York, New York, USA.; 4 Microchemistry & Proteomics Core at Memorial Sloan Kettering Cancer Center, New York, New York, USA.; 5 Hematology and Oncology Division, Weill Cornell Medicine, New York, New York, USA.; 6 Division of Endocrinology, Diabetes, and Metabolism, Beth Israel Deaconess Medical Center and Harvard Medical School, Boston, Massachusetts, USA.; 7 David Z. Rosensweig Genomics Research Center, Hospital for Special Surgery, New York, New York, USA.; 8 Department of Medicine, Weill Cornell Medicine, New York, New York, USA.

**Keywords:** Aging, Autoimmunity, Immunology, Cell stress, Immunoglobulins, Protein kinases

## Abstract

The mechanisms utilized by differentiating B cells to withstand highly damaging conditions generated during severe infections, like the massive hemolysis that accompanies malaria, are poorly understood. Here, we demonstrate that ROCK1 regulates B cell differentiation in hostile environments replete with pathogen-associated molecular patterns (PAMPs) and high levels of heme by controlling 2 key heme-regulated molecules, BACH2 and heme-regulated eIF2α kinase (HRI). ROCK1 phosphorylates BACH2 and protects it from heme-driven degradation. As B cells differentiate, furthermore, ROCK1 restrains their pro-inflammatory potential and helps them handle the heightened stress imparted by the presence of PAMPs and heme by controlling HRI, a key regulator of the integrated stress response and cytosolic proteotoxicity. ROCK1 controls the interplay of HRI with HSP90 and limits the recruitment of HRI and HSP90 to unique p62/SQSTM1 complexes that also contain critical kinases like mTOR complex 1 and TBK1, and proteins involved in RNA metabolism, oxidative damage, and proteostasis like TDP-43. Thus, ROCK1 helps B cells cope with intense pathogen-driven destruction by coordinating the activity of key controllers of B cell differentiation and stress responses. These ROCK1-dependent mechanisms may be widely employed by cells to handle severe environmental stresses, and these findings may be relevant for immune-mediated and age-related neurodegenerative disorders.

## Abstract 2

We find that ROCK1, a serine/threonine kinase, plays a critical role in protecting B cells when exposed to damaging and stressful environments.

## Graphical abstract

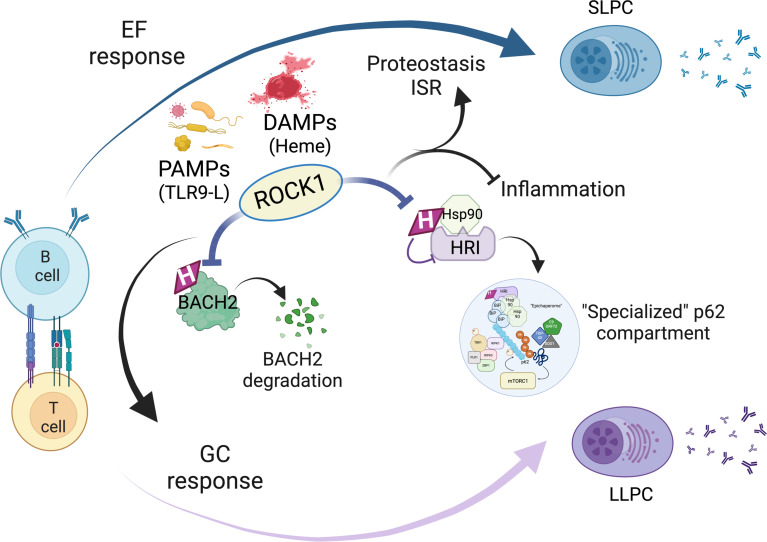

## Introduction

Precise orchestration of B cell differentiation is critical for protective immunity (1). Once activated, B cells can either migrate to extrafollicular (EF) areas and differentiate into short-lived plasmablasts/plasma cells (PB/PC) or to the follicle, where they form germinal centers (GC), eventually becoming high-affinity long-lived plasma cells or memory B cells (MBCs). B cells differentiating via the EF route include B cells that express CD11c and/or T-bet, also known as ABCs, DN2, or atypical B cells (atBCs) (2, 3). Disrupting B cell differentiation is a well-known strategy employed by pathogens to evade immune defenses as observed in malaria caused by *Plasmodium* parasites, which is accompanied by several disturbances ranging from exuberant polyclonal plasmablast responses to enhanced accumulation of atBCs (4). These alterations result in impaired long-lasting immunity allowing for repeated infections.

Severe pathogens can leverage the complex inflammatory environment elicited during the infection to influence B cell differentiation and the establishment of protective immunity. Among the environmental stressors faced by B cells during infections are large amounts of extracellular heme, a critical damage-associated molecular pattern (DAMP) released during the hemolysis triggered by *Plasmodium* and several other pathogens. Interestingly, physiologic levels of heme have recently emerged as an important factor in B cell differentiation (5, 6). This is partly due to the ability of heme to bind to and promote the degradation of BACH2, a transcription factor that not only regulates the expression of the heme-metabolic enzyme HO-1 (encoded by *Hmox1*) but also controls B cell differentiation by repressing the expression of B lymphocyte-induced maturation protein-1 (BLIMP1; encoded by *Prdm1*), thus preventing premature PC differentiation (7). Besides changes in BACH2-controlled transcriptional networks, B cell terminal differentiation also requires the coordinated execution of pathways aimed at handling the increased metabolic requirements and high rate of protein synthesis needed for robust and durable antibody secretion (8). Implementation of this program occurs in distinct phases whereby an X-box-binding protein 1–independent (XBP1-independent) “anticipatory” unfolded protein response (UPR) begins in activated B cells and is followed by the classical IRE1α-XBP1–dependent UPR during the early stages of PC differentiation (9). The “anticipatory” UPR is controlled by mTOR complex 1 (mTORC1), whose activity needs to be precisely controlled during terminal B cell differentiation to ensure adequate PC formation and maximal antibody secretion. Indeed, while mTORC1 is initially critical for PC generation, persistent mTORC1 activation, as observed with activating mutations in PI3K, a crucial upstream regulator of mTORC1, leads to decreased PC survival because of impaired autophagy and increased ER stress (10, 11). The mechanisms that fine-tune mTORC1 activity in differentiating B cells are poorly understood.

Besides PI3K, mTORC1 activation also depends on the presence of amino acids, which enable the recruitment of mTORC1 to the lysosome (12). This repositioning is controlled in a complex manner and can be mediated by a docking system that relies on p62/SQSTM1 (hereafter termed p62), an adaptor that binds raptor and positions mTOR near TNF receptor associated factor 6 (TRAF6), resulting in mTOR activation via K63-linked polyubiquitination (13, 14). Recruitment and activation of mTORC1 by p62 is facilitated by the multidomain structure of p62, a feature that enables this protein to undergo phase separation, mediate the formation of membrane-less condensates, and function as a central signaling hub positioned at the intersection of pathways that regulate not only mTORC1 activation but also inflammation, autophagy, and proteostasis (15, 16). Precise coordination of these processes is critical during intense tissue damage and may be particularly important for secretory cells like PCs, which need to meet the high bioenergetic demands of antibody production even when exposed to dwindling resources and increased stress.

Severe pathogens often manipulate host defenses by targeting RhoA GTPases, whose disarming in innate cells leads to inflammasome activation because of the inhibition of their downstream effectors, the PKN1/2 kinases (17, 18). RhoA signaling also activates another key pair of serine-threonine kinases, ROCK1 and ROCK2, which are well-known controllers of cytoskeletal dynamics. Although intensely investigated in the nonhematopoietic system (19, 20), few studies, mostly focused on ROCK2, have assessed their role in B cells. In this compartment, ROCK2 is activated in response to adaptive signals such as the engagement of CD40 and regulates the proper positioning and cholesterol biosynthesis of GC B cells and PC differentiation (21, 22). These effects have been linked to the ability of ROCK2 to phosphorylate either interferon regulatory factor 8 (IRF8) or IRF4 depending on the stage of B cell differentiation (21, 22). While ROCK1 and ROCK2 share a highly homologous N-terminal kinase domain, they exhibit a lower degree of similarity in the remainder of the molecule and are encoded by different genes, suggesting that each family member can mediate specific functions. Whether ROCK1 helps coordinate B cell activation and differentiation is, however, unknown.

Here, we demonstrate that ROCK1 plays a role in controlling B cell responses in very damaging environments accompanied by high levels of pathogen-associated molecular patterns (PAMPs) and DAMPs like heme. In the absence of ROCK1, B cells exposed to these conditions exhibit altered differentiation, functional capabilities, and stress responses. Notably, absence of ROCK1 results in the aberrant assembly of p62 complexes enriched in critical kinases and molecules involved in RNA metabolism, oxidative damage, and proteostasis. ROCK1 regulates these processes by controlling 2 key heme-regulated molecules, the transcription factor BACH2 and the heme-regulated eIF2α kinase (HRI). These studies thus uncover a surprising role for ROCK1 in the regulation of pathways that enable B cells to efficiently cope with severe damaging conditions and establish durable humoral responses.

## Results

### B cell ROCK1 regulates humoral responses upon immunization.

**Figure 1:**
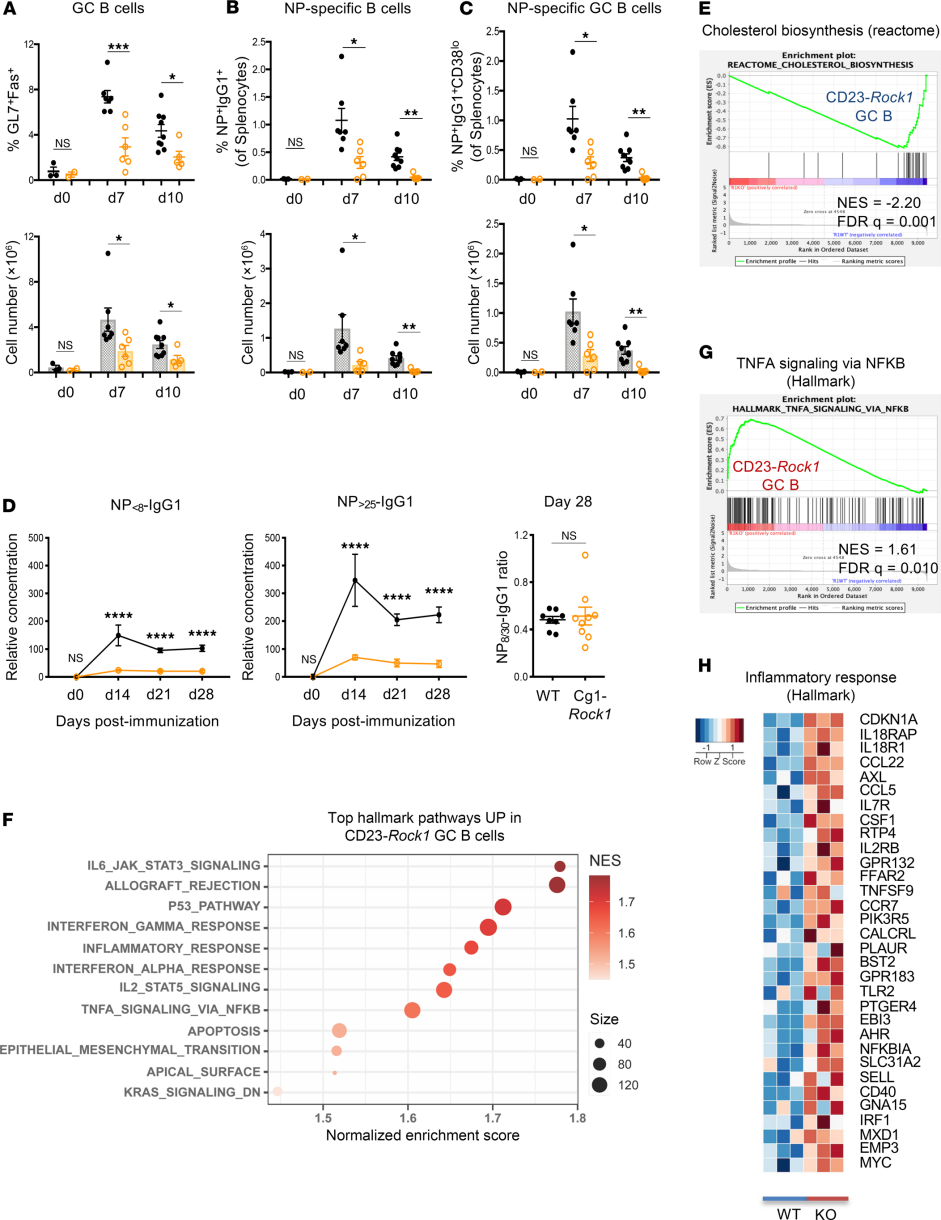
B cell ROCK1 regulates humoral responses during TD immunization. (**A**–**E**) WT *(black)* and Cg1-*Rock1* *(orange)* mice were immunized intraperitoneally (ip) with 100 mg NP-CGG for 7–28 days as indicated. Pooled quantifications of germinal center (GC) B cells (**A**; B220^+^GL7^+^Fas^+^), NP-specific B cells (**B**; B220^+^IgM^–^IgD^–^Gr1^–^IgG1^+^NP^+^), NP-specific GC B cells (**C**; B220^+^IgM^–^IgD^–^Gr1^–^IgG1^+^NP^+^CD38^lo^) from WT and Cg1-*Rock1* mice as assessed by flow cytometry. Data pooled from 7 WT and 6 Cg1-*Rock1* mice across 2 independent experiments and show mean ± SEM; *P* value by unpaired 2-tailed *t* tests. (**D**) ELISA data showing relative concentrations of NP_<8_-IgG1 and NP_>25_-IgG1 in the serum of the indicated mice at days 0–28 after immunization. Data pooled from 4 mice at day 14 and 8 mice from days 0, 21, and 28 per genotype across 2 independent experiments and show mean ± SEM; *P* value by 2-way ANOVA followed by Holm-Šídák test for multiple comparisons. (**E**–**H**) WT or CD23-*Rock1* mice were immunized ip with 100 μg NP-CGG, and GC B cells (B220^+^GL7^+^CD38^lo^) were sorted at day 7 for bulk RNA-Seq. Data shown are from 3 independent experiments. (**E**) GSEA plot shows the downregulation of the REACTOME_CHOLESTEROL_BIOSYNTHESIS pathway in CD23-*Rock1* GC B cells. (**F**) Dot plot shows the top enriched HALLMARK pathways upregulated in CD23-*Rock1* GC B cells as compared with WT GC B cells at FDR < 0.1. (**G**) GSEA plot showing the enrichment of the HALLMARK_TNFA_signaling_via_NFKB in CD23-*Rock1* GC B cells. (**H**) Heatmap of the *z*-score–scaled expression of genes enriching the HALLMARK Inflammatory response pathway in CD23-*Rock1* GC B cells. *P value < 0.05, **P value < 0.01, ***P value < 0.001, and ****P value < 0.0001.

Since B cells express both ROCK1 and ROCK2 (21) (Supplemental Figure 1A; supplemental material available online with this article; https://doi.org/10.1172/jci.insight.180507DS1), we employed a genetic approach to specifically investigate the role of B cell ROCK1 in humoral responses. To this end, we generated *CD23Cre.Rock1^fl/fl^* mice (termed CD23-*Rock1*) and compared them with *Rock1^fl/fl^* (WT) mice. ROCK1 was efficiently deleted in B cells, and there was no compensatory increase in ROCK2 activity by in vitro kinase assays (IVKs) (Supplemental Figure 1, A–C). Except for a small decrease in marginal zone B cells, CD23-*Rock1* mice displayed normal B cell populations in the bone marrow and spleen at baseline (Supplemental Figure 1, D and E). However, after intraperitoneal immunization with a T cell–dependent (TD) antigen, NP-CGG, CD23-*Rock1* mice exhibited fewer GC B cells than WT mice (Supplemental Figure 1F). We also generated *Rock1^fl/fl^* mice expressing Cγ1-Cre (termed Cg1-*Rock1* mice) to induce deletion during the early stages of GC B cell differentiation. Similarly to Cg1-*Rock2* mice (21), TD immunization of Cg1-*Rock1* mice resulted in decreased total and antigen-specific GC B cells (Figure 1, A–C). Unlike mice lacking B cell ROCK2 (21), the ratio of dark zone to light zone GC B cells was, however, not affected by the absence of ROCK1 (Supplemental Figure 1G). No significant differences in the mutation rate were observed between WT and Cγ1-*Rock1* GC B cells (Supplemental Figure 1H), suggesting that ROCK1 is not required for somatic hypermutation. Cg1-*Rock1* mice also exhibited a decrease in NP-specific IgG-producing antibody-secreting cells, and lower titers of NP-specific antibodies, though the ratio of high-affinity to total NP-specific antibodies was unchanged (Figure 1D and Supplemental Figure 1I). Lack of B cell ROCK1, furthermore, did not affect the T follicular helper cell/T follicular regulatory cell ratio or the frequencies of cytokine-producing T cells (Supplemental Figure 1, J and K). These data thus suggest that, similarly to ROCK2 (21), B cell ROCK1 functions in a cell-intrinsic manner to regulate optimal GC formation after immunization.

To gain insights into the mechanisms employed by ROCK1 to control GC formation, we next performed bulk RNA-Seq on sorted GC B cells from immunized WT and CD23-*Rock1* mice (Supplemental Figure 1L). Key GC markers were comparable in WT and CD23-*Rock1* GC B cells (Supplemental Figure 1M). Similar to CD23-*Rock2* GC B cells (21), gene set enrichment analysis (GSEA) revealed that the only downregulated pathway (FDR < 0.1) in CD23-*Rock1* GC B cells was cholesterol biosynthesis (Figure 1E), indicating that both ROCKs participate in the control of this critical metabolic pathway in GC B cells. In contrast to the selective downregulation of only 1 major pathway, lack of ROCK1 led to the upregulation of several pathways in GC B cells (Figure 1F). Some of these pathways, such as HALLMARK-Epithelial Mesenchymal Transition, were related to the known cytoskeletal role of the ROCKs. Interestingly, the most upregulated pathways in CD23-*Rock1* GC B cells included several pro-inflammatory pathways such as HALLMARK-Inflammatory response and HALLMARK-TNFA_signaling_via_NFKB and several targets related to inflammation (e.g., *Ccl22*) (Figure 1, F–H, and Supplemental Figure 1L). These findings, thus, surprisingly suggest that ROCK1 may limit the pro-inflammatory profile of GC B cells.

### B cell ROCK1 regulates humoral responses and pathology during experimental malaria.

**Figure 2:**
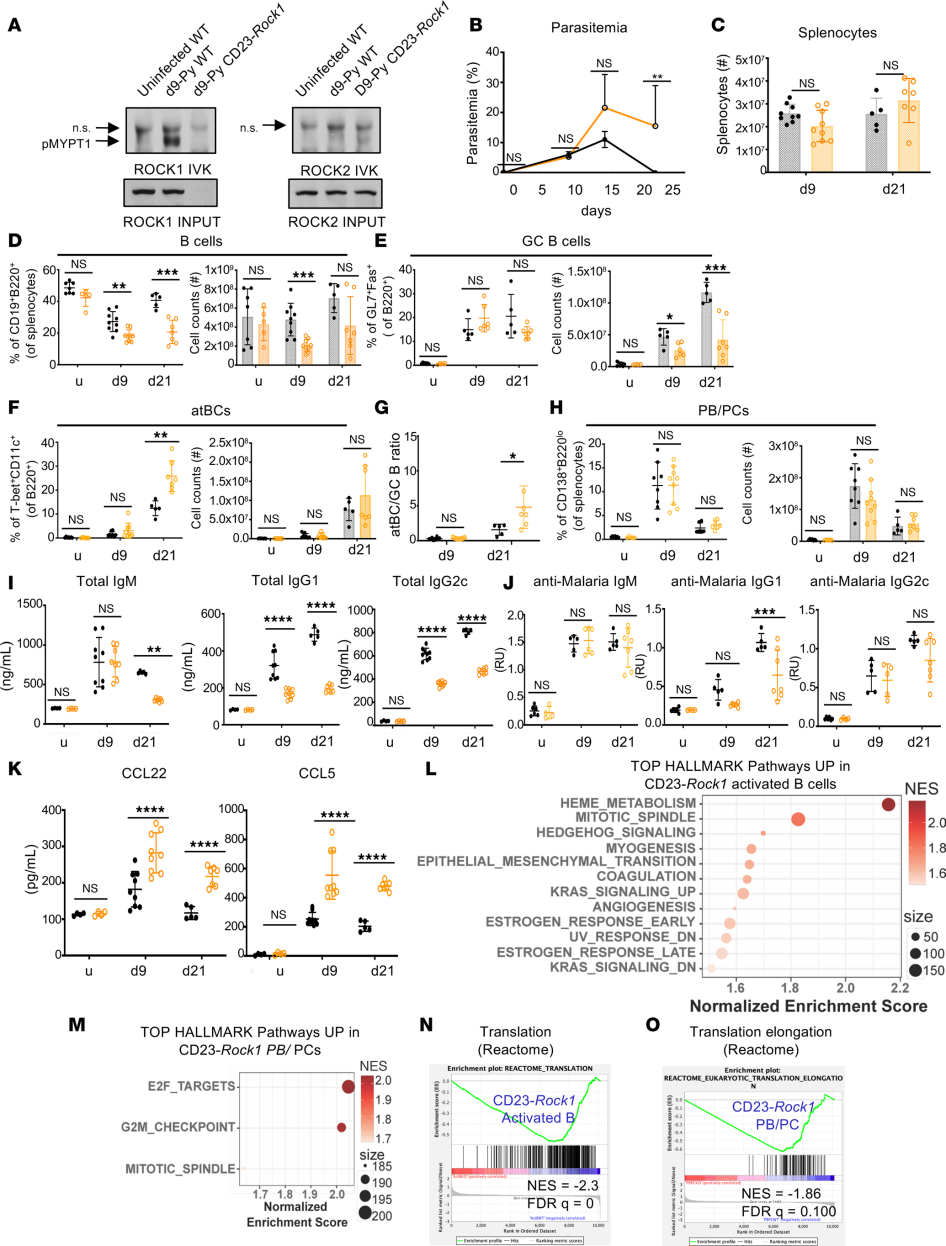
ROCK1 controls humoral responses to *Plasmodium* infection. WT (black) or CD23-*Rock1* (orange) mice were infected with 10^6^ *Plasmodium yoelii* 17XNL-infected erythrocytes. Uninfected (u) or infected mice at the indicated times pi were analyzed. (**A**) ROCK1 and ROCK2 activity in purified CD23^+^ splenic B cells at day 9 pi. Immunoblotting shows p-MYPT1 in IVKs and total ROCK1 and ROCK2 in inputs. Data representative of 3 independent experiments. n.s., nonspecific band; p-, phosphorylated. (**B**) Parasitemia. Data from at least 5 mice per day and per genotype across 3 independent experiments and show mean ± SEM; *P* value by nonparametric Mann-Whitney test between the 2 genotypes for each day of infection. (**C**) Total splenocyte numbers. (**D**–**H**) B cell populations by FACS. Frequencies (symbols) and total cell numbers (bars) of splenic B cells (CD19^+^) (**D**), GC B cells (B220^+^GL7^+^Fas^+^) (**E**), atBCs (B220^+^CD11c^+^T-bet^+^) (**F**), PB/PC (B220^int^ CD138^+^) (**H**). (**G**) Ratio of atBC to GC B cells. Data from at least 5 mice per day and per genotype across 3 independent experiments and show mean ± SEM; *P* value by unpaired 2-tailed *t* tests. (**I**–**K**) Total IgM, IgG1, and IgG2c levels (**I**); anti-malaria IgM, IgG1, and IgG2c relative levels (**J**); and CCL22 and CCL5 levels (**K**) in the sera by ELISA. Data from at least 5 mice per day and per genotype across 3 independent experiments and show mean ± SEM; *P* value by 2-way ANOVA followed by Holm-Šídák test for multiple comparisons. (**L**–**O**) RNA-Seq of splenic B cell populations sorted from WT or CD23-*Rock1* mice at day 9 pi. Data are from 3 independent experiments. (**L** and **M**) Top enriched HALLMARK pathways upregulated in CD23-*Rock1* compared with WT for activated B cells (**L**) and PB/PCs (**M**) at FDR < 0.1. (**N** and **O**) GSEA enrichment plots representing selected downregulated pathways in CD23-*Rock1* activated B cells (**N**) and PB/PCs (**O**). *P value < 0.05, **P value < 0.01, ***P value < 0.001, and ****P value < 0.0001.

The enhanced pro-inflammatory profile of ROCK1-deficient B cells upon a mild challenge like immunization led us to assess how B cell ROCK1-deficient mice would respond to a more complex and hostile milieu. We opted to employ *Plasmodium yoelii 17XNL* (*P*. *yoelii*), a nonlethal self-healing malaria model, which, in C57BL/6 mice, leads to RBC destruction and hemolysis, severe anemia, and parasitemia mimicking features observed in malaria-naive individuals infected with human *Plasmodium* species (23–25). An assessment of ROCK activity revealed that WT B cells increased ROCK1, but not ROCK2, activation as a physiologic response to this parasite at acute day 9 postinfection (pi) (Figure 2A and Supplemental Figure 2A). We next infected WT and CD23-*Rock1* mice with *P*. *yoelii* and analyzed them at acute day 9 pi and at late day 21 pi when mice are normally in a convalescent phase. Although parasitemia levels were similar at day 9, lack of B cell ROCK1 impaired resolution of the infection at day 21 (Figure 2B). Total splenic B cells decreased to a greater extent in CD23-*Rock1* than in WT mice at day 9 pi and did not recover as readily at day 21 pi, and GC B cells were significantly reduced at both time points (Figure 2, C–E). Expansion of atBCs at day 21 pi was unaffected by the absence of B cell ROCK1, resulting in a relative increase in atBCs over GC B cells at day 21 pi (Figure 2, F and G). Only minor decreases in total CD4^+^ and T_FH_ cells were observed (Supplemental Figure 2, B and C). Despite a comparable expansion of PB/PCs at day 9 pi in WT and CD23-*Rock1* mice, CD23-*Rock1* mice exhibited marked decreases in total IgG1 and IgG2c at both day 9 and day 21 and in total IgM at day 21 (Figure 2, H and I). Absence of B cell ROCK1 also resulted in lower titers of anti-malaria IgG1 antibodies but not of anti-malaria IgG2c antibodies, an isotype classically produced by atBCs (Figure 2J). Thus, in this malaria model, B cell ROCK1 is important for resolution of the infection, and its absence affects B cell differentiation and the robust polyclonal antibody responses known to accompany this infection (24, 25).

The increased pro-inflammatory profile of CD23-*Rock1* GC B cells also prompted us to specifically assess the production of CCL22 and CCL5 in response to *P*. *yoelii* infection. CD23-*Rock1* exhibited significantly higher serum levels of these chemokines than WT mice at both day 9 and day 21 pi (Figure 2K). CD23-*Rock1* mice furthermore developed more severe disease as indicated by persistent anemia, thrombocytopenia, increases in IFN-γ–producing CD4^+^ T cells, and increased spleen weight at day 21 pi and required early euthanasia (Supplemental Figure 2, D–F). CD23-*Rock1* mice also displayed increased hepatonecrosis and sinusoidal enlargement in the liver despite similar levels of malaria pigment (hemozoin) deposition (Supplemental Figure 2, G and H). Thus, B cell ROCK1 is important for limiting pathology in experimental malaria.

To further investigate the B cell abnormalities, we sorted B cells representing distinct stages of differentiation (naive, activated, and PB/PC) (24) from day 9 *P*. *yoelii–*infected WT and CD23-*Rock1* mice and performed bulk RNA-Seq (Supplemental Figure 2I). WT and CD23-*Rock1* B cell populations exhibited similar proliferative capabilities and key markers (Supplemental Figure 2, J and K). While only few differentially expressed genes (DEGs) were detected in naive B cells, an increasing number of DEGs could be observed in activated B cells from WT and CD23-*Rock1* mice; GSEA revealed that absence of ROCK1 led to the upregulation of signatures related to heme metabolism, cytoskeletal processes, and pathways related to mitotic spindle assembly and the G2/M checkpoint (Figure 2L). Enrichment in these pathways as well as in E2F targets was also detected in PB/PCs lacking ROCK1 (Figure 2M). Only few downregulated pathways were observed in the absence of ROCK1 in activated B cells and PB/PCs. They were largely related to translation and oxidative phosphorylation (Figure 2, N and O). Thus, in addition to the pathways uncovered by the immunization experiments, the damaging and stressful environment of experimental malaria revealed that B cell ROCK1 also regulates pathways related to their response to heme and programs important for coping with the increased protein synthesis and metabolic demands of antibody-secreting cells.

### ROCK1 regulates the heme-sensing transcription factor BACH2.

**Figure 3:**
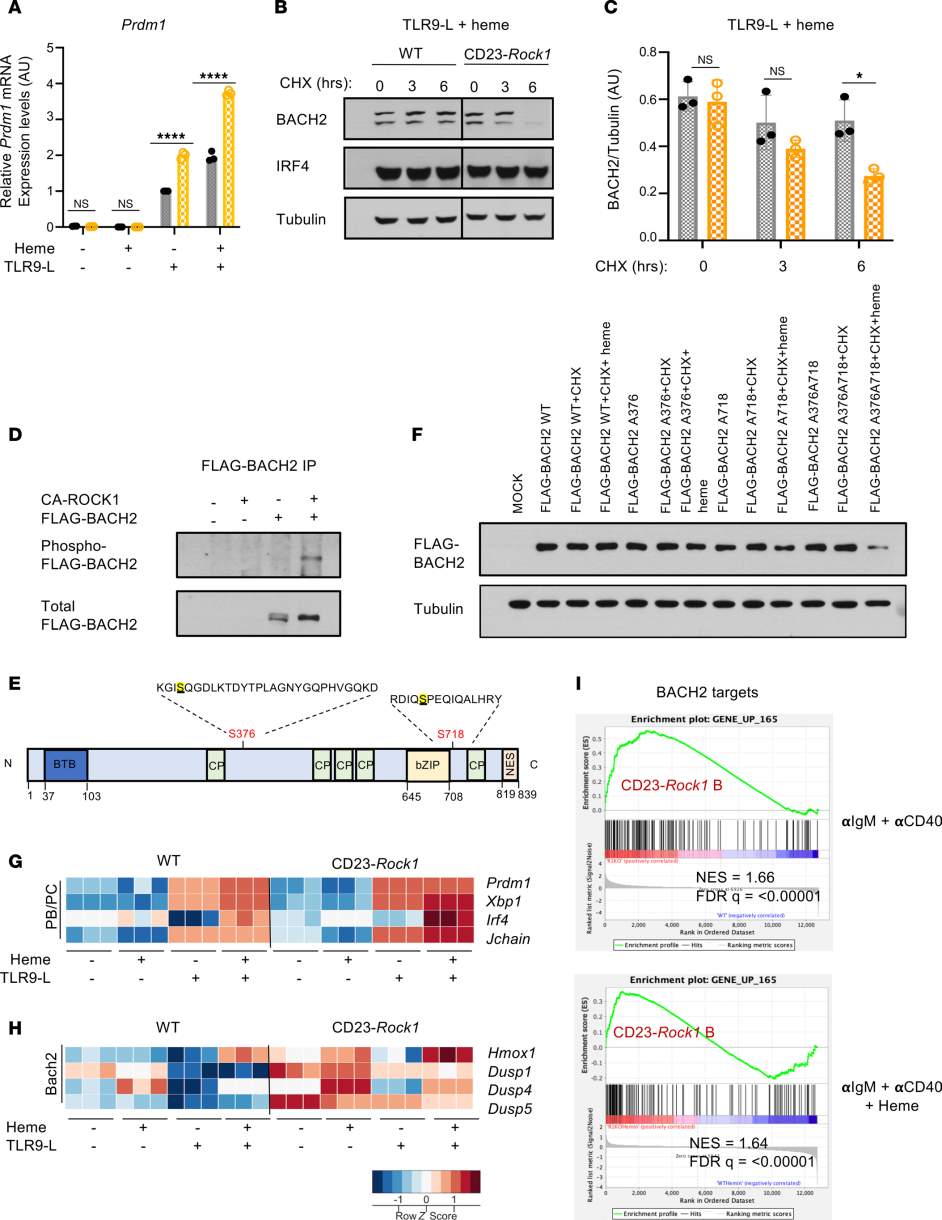
ROCK1 phosphorylates BACH2 and controls its stability. Purified CD23^+^ B cells from WT (black) and CD23-*Rock1* (orange) mice were cultured with αIgM (5 μg/mL) + αCD40 (5 μg/mL), ± combinations of a TLR9-L (1 μg/mL) and heme (60 μM) for 3 days. (**A**) RT-qPCR showing *Prdm1* expression relative to WT TLR9-L treatment, whose value was set at 1. Data are from 3 independent experiments and show mean ± SEM; *P* value by 2-way ANOVA followed by Holm-Šídák test for multiple comparisons. -L, ligand; Prdm1, PR/SET domain 1; RT-q, quantitative reverse transcription. (**B**) Representative immunoblot of BACH2 protein levels in the presence of cycloheximide (CHX) added on day 3 to αIgM+αCD40+TLR9-L+heme conditions for 0, 3, or 6 hours. (**C**) Densitometry ratio of BACH2 to tubulin levels. Data pooled from 3 independent experiments and show mean ± SEM; *P* value by 2-way ANOVA followed by Holm-Šídák test for multiple comparisons. (**D**) FLAG-tagged BACH2 was immunoprecipitated from 293T cells and incubated with CA-ROCK1. p-BACH2 was detected with an anti–phospho-serine Ab recognizing a consensus site shared by ROCK1 and PKA followed by reprobing with an anti-BACH2 Ab. Data are representative of 3 independent experiments. (**E**) Schematic diagram (adapted from https://mutagenetix.utsouthwestern.edu/phenotypic/phenotypic_rec.cfm?pk=3232) showing the putative ROCK phosphorylation sites in murine BACH2 identified by mass spectrometry. (**F**) 293T cells were transfected with constructs expressing FLAG-tagged wild-type BACH2 (WT) or mutants of BACH2 (S376A=A376, S718A=A718, or both S376A and S718A=A376A718). Transfectants were treated ± cycloheximide (CHX) ± heme for 7 hours at day 2 after transfection. Data are representative of 3 independent experiments. (**G**) Heatmap of the *z*-score–scaled expression of selected genes involved in PB/PC differentiation in stimulated WT and CD23-*Rock1* B cells. (**H**) Heatmap of the *z*-score–scaled expression of selected BACH2 targets in stimulated WT and CD23-*Rock1* B cells. (**I**) GSEA plots show the enrichment of BACH2 targets (26) in CD23-*Rock1* B cells stimulated as indicated. *P value < 0.05 and ****P value < 0.0001.

Upregulation of a heme metabolism signature by ROCK1-deficient B cells exposed to malaria suggested a potential role for ROCK1 in regulating how B cells respond to the hemolysis that accompanies this infection. This led us to explore whether ROCK1 could regulate BACH2, a heme-regulated transcription factor that not only represses BLIMP1 (7) but also controls the expression of the heme metabolic enzyme HO-1 (26). To investigate this possibility, we stimulated WT and ROCK1-deficient B cells in vitro in the presence/absence of key cues released during malaria, the CpG PAMP recognized by TLR9, and heme, a critical DAMP released during the hemolysis that the infection triggers. WT and CD23-*Rock1* B cells exhibited similar viability except for a slight increase in apoptosis when cultured with anti-IgM (αIgM) + αCD40 + heme (Supplemental Figure 3, A and B). Interestingly, B cells lacking ROCK1 expressed higher levels of *Prdm1* mRNA than WT B cells in the presence of CpG, and this effect was augmented by the presence of heme (Figure 3A). While no significant changes in *Bach2* transcripts were observed (Supplemental Figure 3C), CD23-*Rock1* B cells exhibited lower levels of BACH2 protein than WT B cells when stimulated with CpG and heme, an effect that was confirmed by adding cycloheximide to block new protein synthesis (Figure 3, B and C, and Supplemental Figure 3, D and E). Thus, ROCK1 helps maintain adequate BACH2 protein levels when B cells are exposed to PAMPs and DAMPs like TLR9 ligands and heme.

BACH2 can be phosphorylated on several serine and threonine residues within its intrinsically disordered region (aa 331–520), which is involved in heme-binding and protein-protein interactions (27). To assess whether ROCK1 could phosphorylate BACH2, we immunoprecipitated FLAG-tagged BACH2 and performed IVKs with constitutively active ROCK1 (CA-ROCK1). Incubation with CA-ROCK1 resulted in the phosphorylation of BACH2 as assessed by immunoblotting with a phospho-serine antibody (Figure 3D). To verify these findings, the immunoprecipitated in vitro phosphorylated BACH2 protein was subjected to mass spectrometry analysis, which revealed that ROCK1 could phosphorylate BACH2 at 2 sites: S376 located in the heme-binding domain just downstream of the first CP motif and S718 just downstream of the bZIP region, which mediates heterodimer formation and DNA binding (27) (Figure 3E). To assess the functional effects of the ROCK1-mediated phosphorylation of BACH2, we generated BACH2 mutants in which S376 and S718 were mutated to alanine either individually or in combination (BACH2A376, BACH2A718, and BACH2A376A718) and assessed their stability in heme-treated HEK293T (293T) cells (which express active ROCK1 in the presence of serum) (Figure 3F and Supplemental Figure 3F). Addition of heme in the presence of cycloheximide to block new protein synthesis did not affect the protein levels of WT BACH2 and exerted only slight effects on the single mutants (BACH2A376 and BACH2A718) but markedly decreased the abundance of BACH2A376A718. Thus, phosphorylation of BACH2 by ROCK1 prevents its degradation in response to heme.

We next performed RNA-Seq on WT and CD23-*Rock1* B cells stimulated in vitro under the various conditions (Supplemental Figure 3, G–J). Exposure to a TLR9 ligand, particularly in the presence of heme, led to higher expression of markers associated with PC differentiation in CD23-*Rock1* than WT B cells, in line with the decreased BACH2 stability and the enhanced *Prdm1* mRNA upregulation observed under those conditions (Figure 3G). B cells from CD23-*Rock1* mice expressing a BLIMP1–yellow fluorescence protein (BLIMP1-YFP) reporter, furthermore, exhibited increased PC differentiation when stimulated with CpG and heme (Supplemental Figure 3K). Interestingly, lack of ROCK1 also affected the expression of other targets normally repressed by BACH2, such as *Hmox1* and *Dusp4*, even when cells were cultured with αIgM + αCD40 alone (Figure 3H and Supplemental Figure 3, L and M). A comparison with a recently published dataset (26) further verified that, under these conditions, absence of B cell ROCK1 resulted in an enrichment in BACH2-repressed targets (Figure 3I). Taken together these data suggest that ROCK1 can modulate the BACH2-controlled transcriptional program by mechanisms beyond controlling BACH2 stability,

### Absence of ROCK1 leads to enhanced pro-inflammatory and mTORC1 signatures.

**Figure 4:**
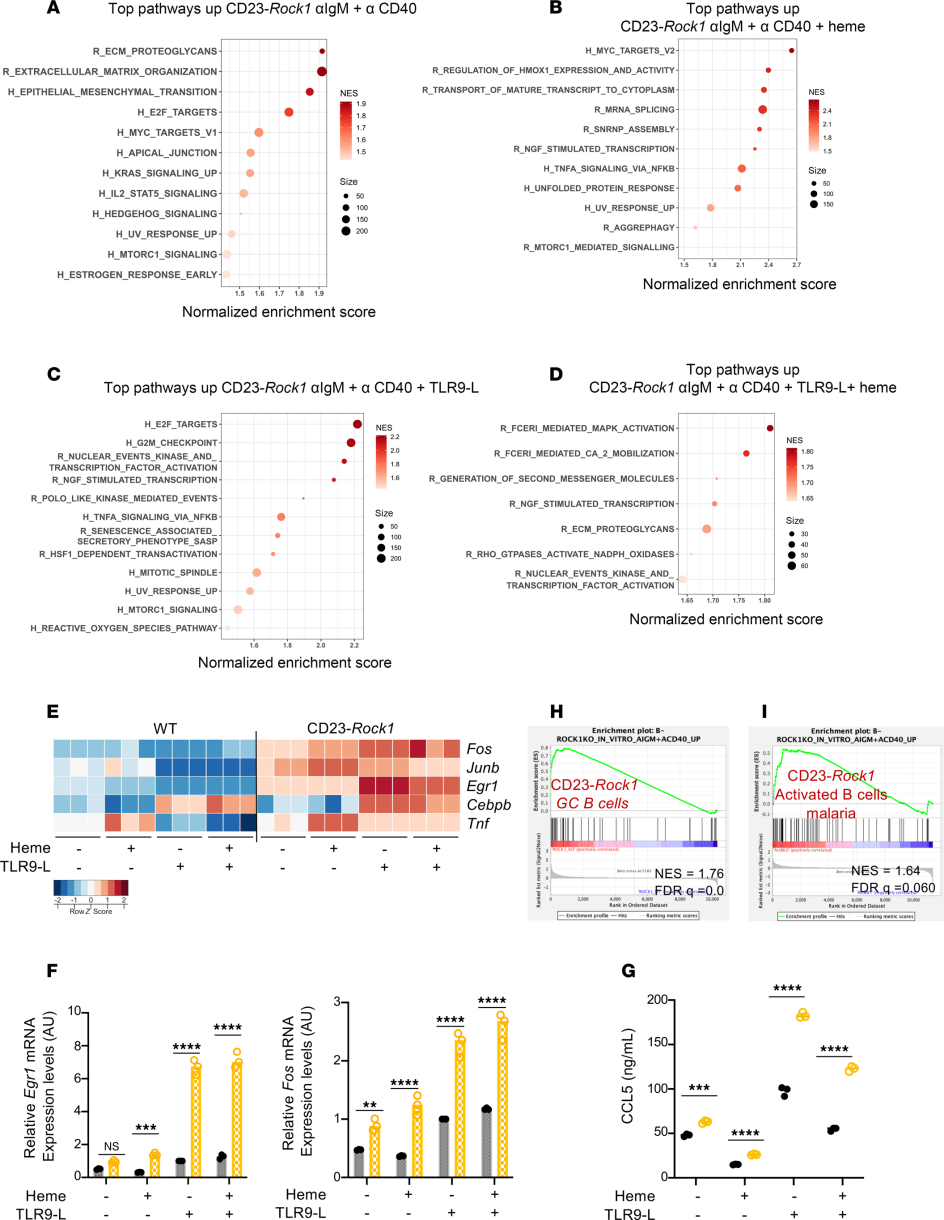
ROCK1-deficient B cells activated in vitro exhibit increased pro-inflammatory and mTORC1-related transcriptional signatures. Purified CD23^+^ B cells from WT and CD23-*Rock1* mice were cultured with αIgM (5 μg/mL) + αCD40 (5 μg/mL), ± combinations of a TLR9-L (1 μg/mL) and heme (60 μM) for 3 days. (**A**–**D**) Top enriched HALLMARK (H) and REACTOME (R) pathways upregulated in stimulated CD23-*Rock1* B cells as compared with WT B cells at FDR < 0.1. (**E**) Heatmap of the *z*-score–scaled expression of selected genes involved in inflammation in stimulated WT and CD23-*Rock1* B cells. (**F**) RT-qPCR showing *Fos* and *Egr1* mRNA expression under the indicated conditions relative to WT TLR9-L treatment, whose value was set at 1. Data are from 3 independent experiments and show mean ± SEM; *P* value by 2-way ANOVA followed by Holm-Šídák test for multiple comparisons. (**G**) CCL5 levels in the supernatants were assessed by ELISA. Data are from 3 independent experiments and show mean ± SEM; *P* value by 2-way ANOVA followed by Holm-Šídák test for multiple comparisons. (**H** and **I**) GSEA plots showing the enrichment of the CD23-*Rock1* B cells stimulated with αIgM + αCD40 gene set in CD23-*Rock1* GC B cells (**H**) and CD23-*Rock1* activated B cells from the experimental malaria model (**I**). **P value < 0.01, ***P value < 0.001, and ****P value < 0.0001.

Since some of the abnormalities exhibited by ROCK1-deficient B cells, such as the enhanced pro-inflammatory profile and the inability to maintain robust antibody secretion, could not be explained if ROCK1 only controlled BACH2, we next leveraged the in vitro system to gain insights into the mechanisms underlying these findings. GSEA employing HALLMARK and REACTOME gene sets (Figure 4, A–D) revealed that CD23-*Rock1* B cells upregulated cytoskeletal pathways, including targets like *Rgs16*, and cell cycle–related pathways like G2/M checkpoint and E2F targets (Figure 4, A–D, and Supplemental Figure 4A). Notably, B cells lacking ROCK1 were enriched for inflammatory pathways like TNFA_signaling_via_NFKB and upregulated the expression of immediate early genes like *Fos* and *Egr1* (Figure 4, A–F). In agreement with these findings, ROCK1-deficient B cells produced higher levels of CCL5 compared with WT B cells (Figure 4G). Some of the alterations could already be detected in cells stimulated with αIgM + αCD40 alone. Presence of TLR9 ligands or heme augmented several of the abnormalities, particularly those related to the acquisition of an enhanced pro-inflammatory profile. In vitro–stimulated CD23-*Rock1* B cells, furthermore, upregulated a similar transcriptional profile to that observed in CD23-*Rock1* B cells upon immunization and *P*. *yoelii* infection (Figure 4, H and I). Thus, key features exhibited by ROCK1-deficient B cells in vivo, such as their enhanced pro-inflammatory profile, were replicated under the in vitro stimulatory conditions.

The GSEA also revealed that, relative to WT B cells, absence of B cell ROCK1 resulted in an enrichment in pathways related to mTORC1 signaling (Figure 4, A–D). Given that mTORC1 controls a preparative UPR in activated B cells that precedes the classical UPR associated with PC differentiation (9), we employed GSEA to assess this stress response. CD23-*Rock1* B cells showed a greater enrichment for the B cell activating UPR signature than WT B cells under most stimulatory conditions, except in the presence of both TLR9 ligands and heme, consistent with the accelerated PC differentiation of these cultures (Supplemental Figure 4B). These data thus suggest that absence of ROCK1 may lead to dysregulated mTORC1 activity and predispose activated B cells to inappropriately implement biochemical programs necessary for the transition to PCs.

### The phospho-proteome of ROCK1-deficient B cells reveals increased mTORC1 activity.

**Figure 5:**
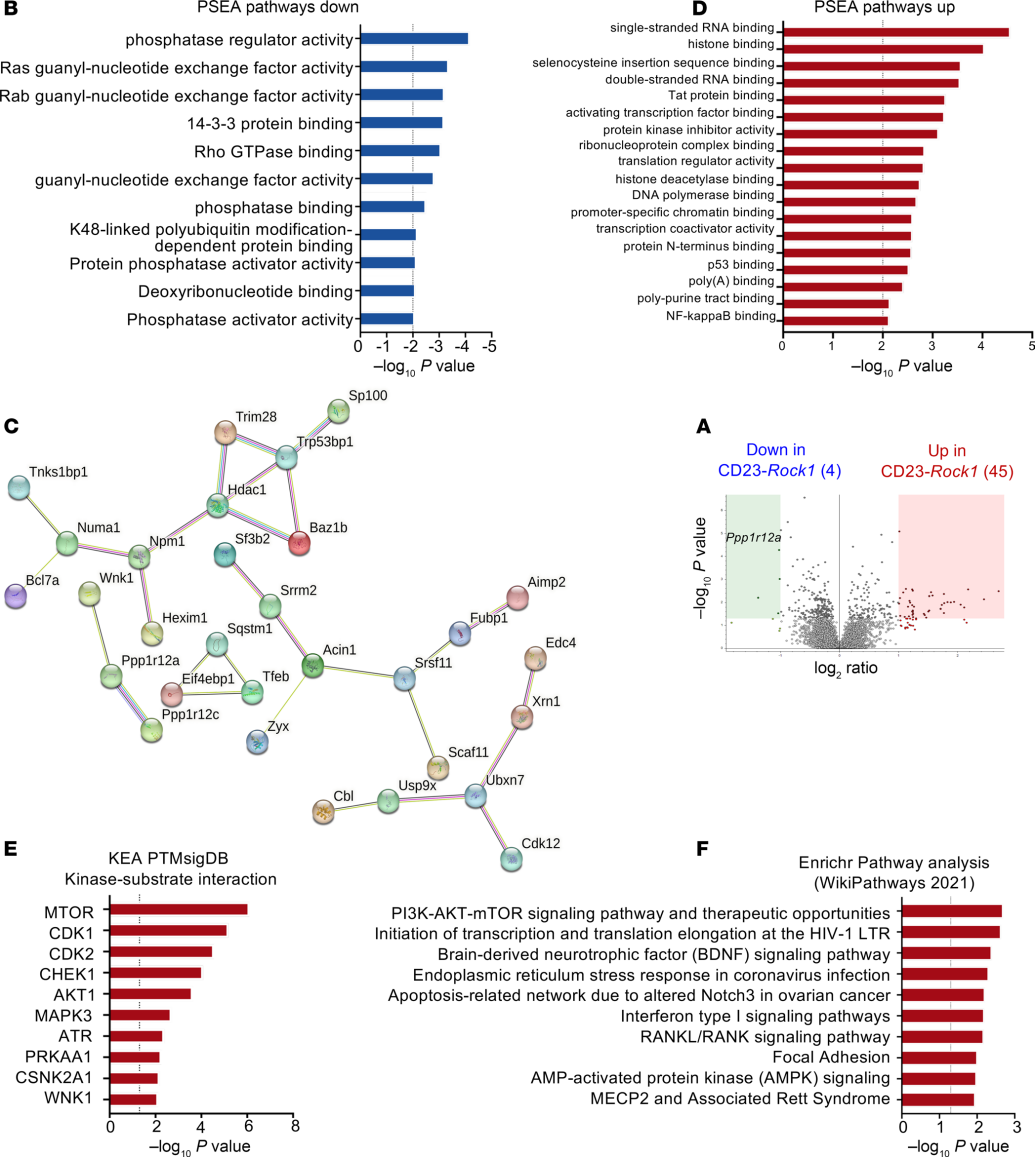
B cell ROCK1 controls a distinctive phospho-proteomic profile. Purified CD23^+^ B cells from WT and CD23-*Rock1* mice were cultured with αIgM (5 μg/mL) + αCD40 (5 μg/mL) for 3 days, and 4 independent replicates were submitted for phospho-proteomic analysis using TMT mass spectrometry. (**A**) Volcano plot shows differentially enriched phospho-proteins in stimulated WT (green rectangle) and CD23-*Rock1* (red rectangle) B cells (log_2_FC > 1, *P* < 0.05). (**B**) Protein set enrichment analysis (PSEA) (*P* < 0.01) of the phospho-proteins downregulated in stimulated CD23-*Rock1* versus WT B cells based on Gene Ontology (GO) database. (**C**) STRING analysis of phospho-proteomic targets (https://string-db.org/). (**D**) PSEA (*P* < 0.01) of the phospho-proteins upregulated in stimulated CD23-*Rock1* versus WT B cells based on GO database. (**E**) Top kinase-substrate interactions from the kinase enrichment analysis (KEA) of the differentially enriched phospho-proteins from stimulated CD23-*Rock1* versus WT B cells (*P* < 0.05). (**F**) Top pathways obtained from the Enrichr pathway analysis of the differentially enriched phospho-proteins from stimulated CD23-*Rock1* versus WT B cells (*P* < 0.05).

To better delineate how ROCK1 controls the biochemical state of activated B cells, we next conducted a phospho-proteomic analysis. In vitro–stimulated WT and CD23-*Rock1* B cells were harvested, then subjected to phospho-serine/phospho-threonine tandem mass tag mass spectrometry (pS/pT TMT) and total proteome TMT, and results were analyzed by Proteome Discoverer. A total of 49 phosphorylated peptides were significantly altered in CD23-*Rock1* B cells compared with WT B cells (Figure 5A). Phosphorylation of 4 peptides was significantly downregulated (log_2_FC > 1, *P* < 0.05) in the absence of ROCK1 (Supplemental Table 1). Two of the downregulated phosphorylation sites were in distinct regulatory subunits of myosin phosphatase (ppp1r12a = MYPT1 and ppp1r12c = MBS85), which are well-known ROCK substrates. Lack of ROCK1 also decreased the phosphorylation status of 2 sites within AKAP13, an X-linked RhoA GEF (28). The site whose phosphorylation was most profoundly downregulated in the absence of ROCK1 was contained within the active site of tissue-nonspecific alkaline phosphatase (TNAP/ALPL) (29), which, in addition to its role in phosphate metabolism, possesses antiinflammatory properties (30). In line with these findings, a protein set enrichment analysis (PSEA) demonstrated enrichment in phosphatase regulator activity as the most downregulated pathway (*P* < 0.01) (Figure 5B). These results thus support the idea that B cell ROCK1 phosphorylates key phosphatases, which include known targets involved in cytoskeletal dynamics like myosin phosphatase and potentially novel targets like TNAP/ALPL.

Surprisingly, absence of B cell ROCK1 also resulted in significant increases in the phosphorylation of several peptides (Supplemental Table 2). Most of the phosphorylated sites were in well-known proteins like 53BP1, 4EBP1, and p62. Several of the targets were functionally connected by STRING analysis (Figure 5C). PSEA pathway analysis demonstrated an enrichment for pathways involved in chromatin regulation, RNA handling, and translation (*P* < 0.01) (Figure 5D). Consistent with these results, many of the processes coordinated by proteins whose phosphorylation is altered in CD23-*Rock1* B cells normally take place in the nucleus, spliceosome, and RNA-processing structures like P-bodies (Supplemental Figure 5, A and B). Stimulation with a TLR9 ligand in addition to αIgM + αCD40 resulted in a smaller number of changes in the pS/pT TMT analysis (Supplemental Figure 5C). Many of the phospho-peptides affected by the lack of ROCK1 upon TLR9 costimulation were similar to those detected in CD23-*Rock1* B cells activated with αIgM + αCD40 alone except for 6 proteins whose phosphorylation was significantly increased specifically in CpG-stimulated cells (Supplemental Table 3) and included components of cytoplasmic granules and PML-nuclear bodies, the ubiquitin ligase Cbl, and aspartate carbamoyltransferase, a rate-limiting enzyme in de novo pyrimidine synthesis. Thus, the ROCK1-regulated phospho-proteome in B cells extends beyond the predicted involvement of this kinase in cytoskeletal dynamics and encompasses several proteins that regulate processes involved in DNA damage, RNA processing and handling, and translational regulation.

Given the unexpected increase in the phosphorylation of several targets in the absence of ROCK1, we employed KEA PTMsigDB and Enrichr pathway analysis to gain insights into the kinases that might phosphorylate these sites. The top kinase-substrate interaction predicted by these algorithms implicated mTOR as the kinase most likely to be responsible for these phosphorylation events (Figure 5, E and F). This finding was corroborated by a survey of PhosphoSitePlus (31), which revealed that many of these phosphorylation events were occurring at sites previously identified in proteomic analyses linked to mTOR signaling and particularly to mTORC1 (Supplemental Table 4). Some of the sites uncovered by this analysis had also been reported in phospho-proteomic studies of PLK1 as well as of TBK1 and RIPK3, kinases that play key roles in cell cycle regulation and inflammatory responses (Supplemental Table 4). These results suggest that the dysregulated transcriptional profile of ROCK1-deficient B cells is coupled with aberrant activation of mTORC1 and, possibly, of other key kinases.

### ROCK1 limits formation of p62 complexes containing key kinases and amyotrophic lateral sclerosis–linked molecules.

**Figure 6:**
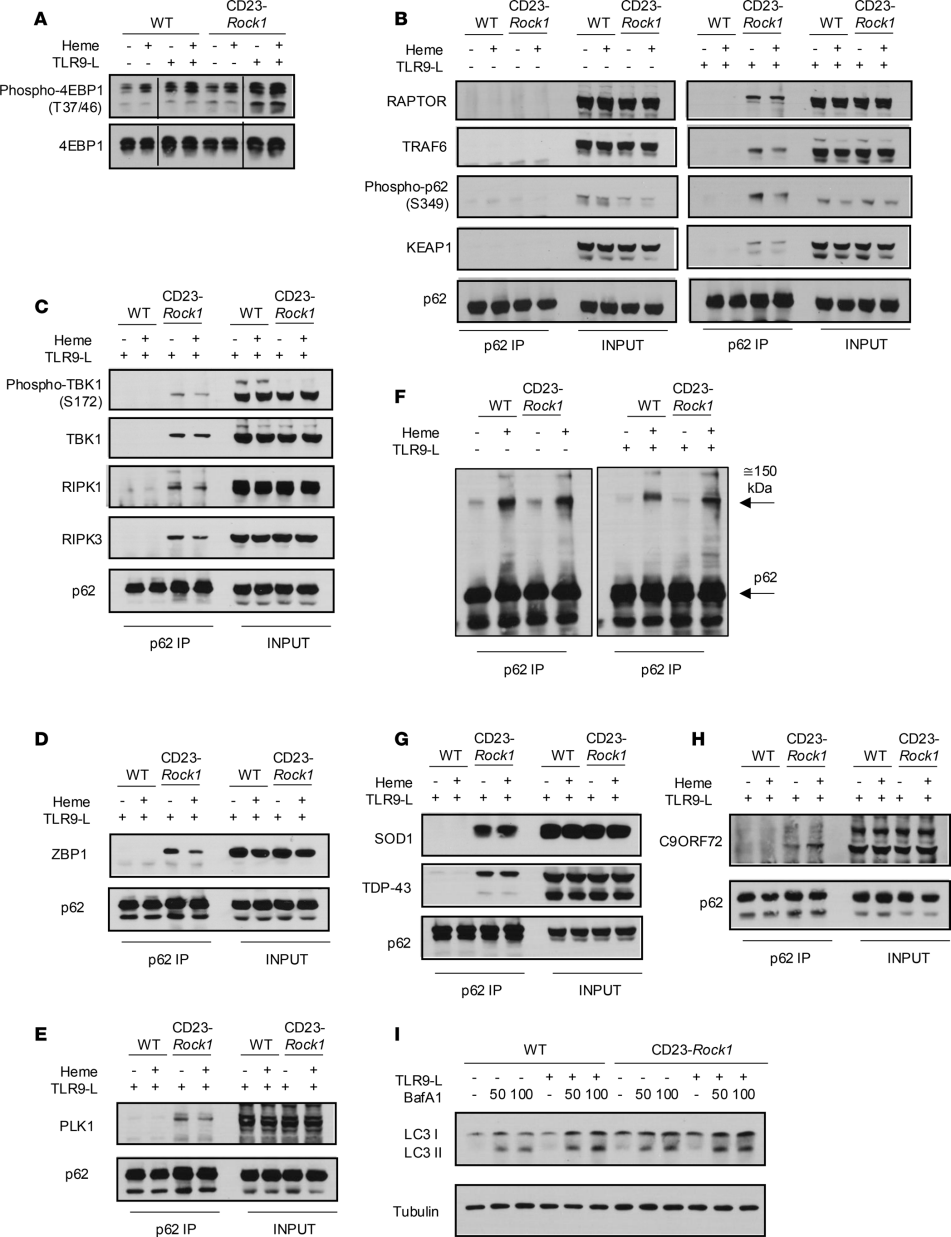
ROCK1 limits the assembly of p62 complexes enriched in mTORC1, ripoptosome components, and ALS-linked molecules. Purified CD23^+^ B cells from WT and CD23-*Rock1* mice were cultured with αIgM (5 μg/mL) + αCD40 (5 μg/mL), ± combinations of a TLR9-L (1 μg/mL) and heme (60 μM) for 3 days. (**A**) Western blotting analysis of the levels of p-4EBP1 in cytoplasmic extracts from WT and CD23-*Rock1* B cells stimulated as indicated. (**B**–**H**) p62 was immunoprecipitated from cytoplasmic extracts of WT or CD23-*Rock1* B cells stimulated as indicated. The precipitates were probed by Western blotting to assess p62 phosphorylation as well as the interaction of p62 with raptor, TRAF6, and Keap1 (**B**); p62 was immunoprecipitated from cytoplasmic extracts of WT or CD23-*Rock1* B cells stimulated as indicated. The precipitates were probed by Western blotting to assess for the presence of phospho-TBK1, TBK1, RIPK1, and RIPK3 (**C**); ZBP1 (**D**); PLK1 (**E**); SOD1 and TDP-43 (**G**); and C9ORF72 (**H**). (**F**) Western blotting analysis of high–molecular weight p62 complexes in WT or CD23-*Rock1* B cells stimulated as indicated. (**I**) Western blots of LC3-I and LC3-II in extracts from WT and CD23-*Rock1* B cells. Cells were treated for 4 hours with DMSO or bafilomycin A1 (BafA1; 50 nM or 100 nM as indicated). All results are representative of 3 independent experiments. ALS, amyotrophic lateral sclerosis

To delineate the mechanisms by which lack of ROCK1 leads to increased mTORC1 activity, we next investigated key steps in the mTORC1 signaling cascade. In agreement with the phospho-proteomic analysis, absence of ROCK1 resulted in higher levels of p-4EBP1, a key downstream effector of mTORC1 (12) (Figure 6A). Phosphorylation of other mTORC1 targets like S6, p70S6K, and ULK1 was instead unchanged (Supplemental Figure 6, A–C). No differences were observed in the phosphorylation of AKT and AMPK, upstream regulators of mTORC1, in WT and CD23-*Rock1* B cells (Supplemental Figure 6, D–F). Furthermore, p-4EBP1 levels in CD23-*Rock1* B cells stimulated with LPS ± heme were similar to WT B cells (Supplemental Figure 6G). These findings suggest that ROCK1 does not affect mTORC1 activity by regulating growth factor–mediated or energy-dependent pathways and that dysregulated mTORC1 activity in the absence of ROCK1 is restricted to a specific subset of TLR ligands.

Activation of mTORC1 in response to nutrients can be mediated by the interaction of raptor with p62 and the subsequent activation of mTOR by TRAF6 (13, 14). An assessment of this complex revealed that p62 did not associate with TRAF6 in WT B cells regardless of the stimulation conditions (Figure 6B). In contrast, p62 strongly interacted with both raptor and TRAF6 in CD23-*Rock1* B cells in the presence of a TLR9 ligand ± heme (Figure 6B). Under these conditions, furthermore, the immunoprecipitated p62 was phosphorylated at S349 (Figure 6B), a site whose phosphorylation is mTORC1 dependent (15), indicating that the raptor–p62–TRAF6 complex formed under these conditions contains active mTORC1. As predicted, phosphorylation of p62 at S349 was accompanied by an increased ability of p62 to interact with Keap1 (Figure 6B). Thus, ROCK1 restrains the assembly of a p62–raptor–TRAF6 complex and fine-tunes mTORC1 activation.

p62 acts as a signaling hub coordinating the activity of several pathways (15, 16). Importantly, p62 can interact with TBK1 and RIPK1, which, together with RIPK3 and ZBP1, are critical components of ripoptosome complexes regulating TNF-α signaling and inflammatory cell death in innate cells (32, 33). Given the enhanced pro-inflammatory profile of CD23-*Rock1* B cells and the increased phosphorylation of TBK1 and RIPK3 targets detected in the phospho-proteome, we thus investigated the presence of these molecules in p62 precipitates. p62 strongly interacted with TBK1, RIPK1, RIPK3, and ZBP1 in CD23-*Rock1* B cells stimulated with a TLR9 agonist ± heme (Figure 6, C and D, and Supplemental Figure 6, H and I). TBK1 in those p62 precipitates was furthermore phosphorylated at S172, suggesting that TBK1 in these complexes is active (33) (Figure 6C). Although MLKL could not be reliably identified in the p62 complexes, no significant differences in its cleavage were observed between WT and CD23-*Rock1* B cells (Supplemental Figure 6J). Furthermore, lactate dehydrogenase levels in the supernatants of CD23-*Rock1* B cells were lower than in those of WT B cells (Supplemental Figure 6K). Since PLK1 can assemble in “mitotic ripoptosomes” with RIPK1 and RIPK3 (34, 35) and CD23-*Rock1* B cells exhibit dysregulation in cell cycle pathways, we also investigated the presence of PLK1 in the p62 precipitates. Stimulation with a TLR9 agonist ± heme led to the accumulation of PLK1 in the p62 complexes of ROCK1-deficient but not of WT B cells (Figure 6E and Supplemental Figure 6L). In the absence of TLR9-L, heme was unable to recruit any of the molecules to p62 despite leading to the formation of high–molecular weight p62 complexes as reported (36) (Figure 6F). In the presence of TLR9-L, however, heme altered the composition of the p62 complexes formed in CD23-*Rock1* B cells, as shown by the decrease in the recruitment of ZBP1 (Figure 6D). Thus, when B cells differentiate in the presence of pathogen-associated signals, ROCK1 limits the formation of p62 multimolecular complexes that contain major kinases and signaling components that control the cell cycle and inflammation.

p62 aggregates are a well-known feature of neurodegenerative disorders like ALS, and p62 can colocalize with other ALS-linked proteins such as TDP-43, SOD1, and C9ORF72 (15), leading us to investigate the presence of these targets in the p62 complexes formed in the absence of ROCK1. Both TDP-43 and SOD1 strongly interacted with p62 in CD23-*Rock1* B cells, a finding that was again primarily restricted to cells stimulated with a TLR9 agonist ± heme (Figure 6G and Supplemental Figure 6M). C9ORF72 was also detected in the p62 precipitates and followed a pattern similar to that of TDP-43 and SOD1 (Figure 6H and Supplemental Figure 6N). Molecules known to be recruited to cytoplasmic granules and P-bodies under stress, such as DEF6 (37), and its homolog SWAP-70, were also present in the p62 complexes (Supplemental Figure 6O). No recruitment of TDP-43 or other key targets like RIPK3 to p62 was observed upon stimulation with LPS, a TLR4 ligand (Supplemental Figure 6P). An assessment of autophagic flux as evaluated by formation of lipidated LC3 (LC3-II), furthermore, did not reveal any substantial differences between WT and CD23-*Rock1* B cells (Figure 6I). Thus, in the presence of specific pathogen-driven stressors, absence of ROCK1 leads to the formation of distinctive p62 complexes, which, in addition to major kinases, contain several proteins involved in RNA metabolism, proteostasis, and oxidative stress, many of which have been linked to ALS pathogenesis.

### ROCK1 controls the heme-regulated kinase HRI, a key sensor of stress and protein aggregation.

**Figure 7:**
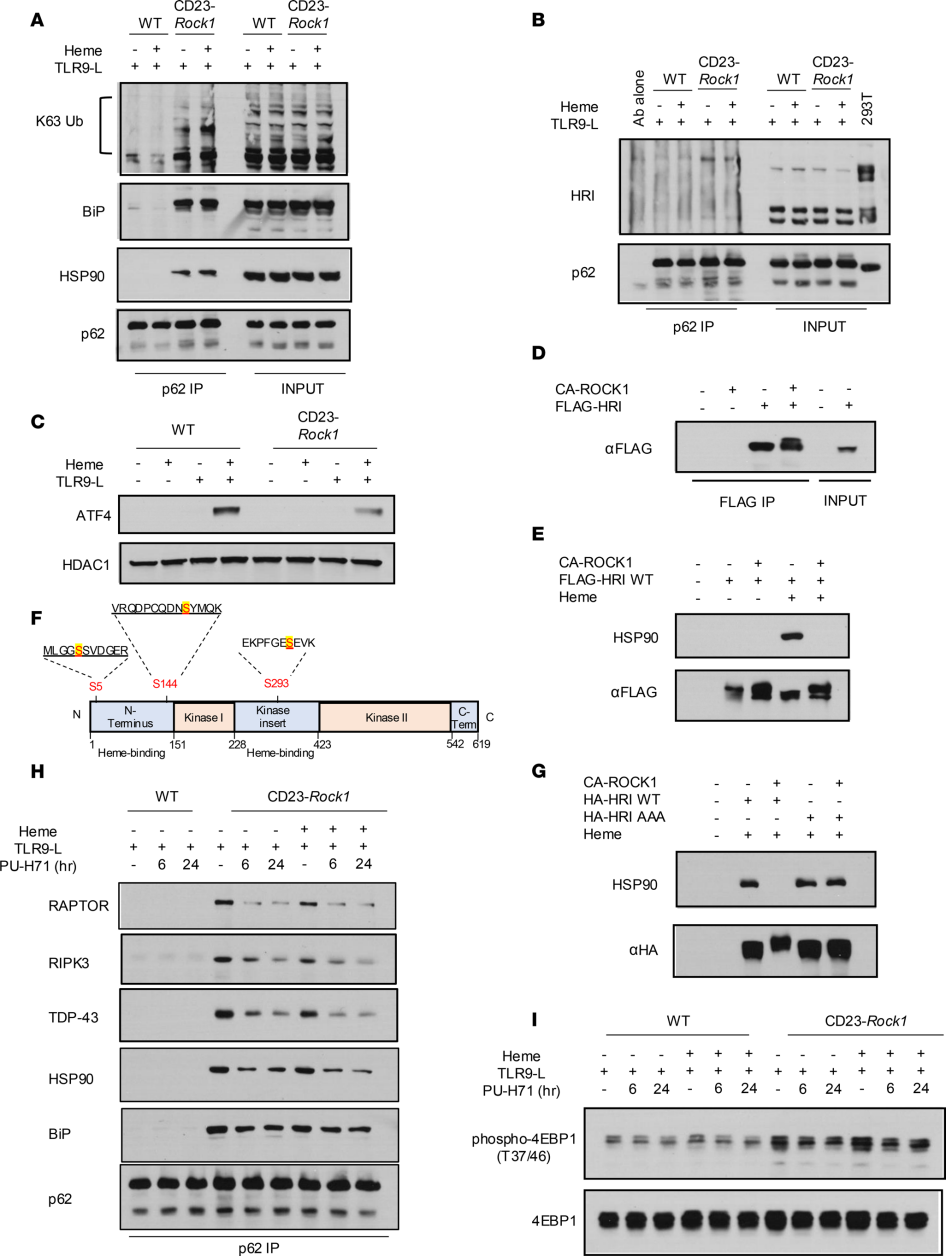
ROCK1 regulates HRI. Purified CD23^+^ B cells from WT and CD23-*Rock1* mice were cultured with αIgM (5 μg/mL) + αCD40 (5 μg/mL) ± combinations of a TLR9-L (1 μg/mL) and heme (60 μM) for 3 days. (**A** and **B**) p62 was immunoprecipitated from cytoplasmic extracts of WT or CD23-*Rock1* B cells. Precipitates were probed by Western blotting for K63-ubiquitinated proteins, BiP, HSP90 (**A**), and HRI (**B**). Results are representative of 3 independent experiments. (**C**) Western blotting analysis of ATF4 levels in extracts from WT and CD23-*Rock1* B cells. Results are representative of 3 independent experiments. (**D**) FLAG-tagged HRI immunoprecipitated from 293T cells was incubated with CA-ROCK1 followed by immunoblotting with an anti-FLAG Ab. Results are representative of 3 independent experiments. (**E**) FLAG-tagged HRI immunoprecipitated from 293T cells stimulated ± heme (60 μM for 4 hours) was incubated with CA-ROCK1 followed by immunoblotting with an anti-HSP90 (upper) or anti-FLAG Ab (lower). Results are representative of 3 independent experiments. (**F**) Schematic diagram (adapted from ref. 48) showing the sites in murine HRI whose phosphorylation was increased upon incubation with CA-ROCK1 by mass spectrometry. (**G**) 293T cells transfected with either HA-tagged wild-type HRI (WT) or an HA-tagged HRI triple mutant (HRI-AAA with S5, S144, and S293 mutated to alanine) were stimulated ± heme (60 μM for 4 hours). Immunoprecipitated HA-tagged HRI WT or HA-tagged HRI-AAA was incubated ± CA-ROCK1 followed by immunoblotting with anti-HSP90 or anti-HA Ab. Results are representative of 3 independent experiments. (**H** and **I**) Purified CD23^+^ B cells were cultured for 3 days with DMSO or PU-H71 (1 μM) added for the last 6 or 24 hours. p62 was immunoprecipitated from cytoplasmic extracts and precipitates probed by immunoblotting for raptor, RIPK3, TDP-43, HSP90, and BiP. (**I**) Western blots of p-4EBP1 levels in cytoplasmic extracts from WT and CD23-*Rock1* B cells stimulated as indicated. Results are representative of 3 independent experiments.

We next investigated the mechanisms by which ROCK1 could regulate the formation of p62 complexes upon exposure to stressors. Since p62 is known to form condensates upon binding ubiquitin chains (38), we first probed the p62 precipitates for the presence of K63-linked ubiquitin chains. Only p62 precipitates from CD23-*Rock1* B cells exposed to TLR9-L ± heme contained K63-ubiquitinated proteins (Figure 7A and Supplemental Figure 7A). Since the ER chaperone BiP (also known as HSPA5/GRP78) can accumulate in the cytoplasm under stress, interact with p62, and promote its oligomerization (39), we also probed the p62 precipitates for BiP. BiP did not interact with p62 in WT B cells but was strongly detected in the p62 complexes formed in CD23-*Rock1* B cells when these cells were stimulated with TLR9-L ± heme (Figure 7A and Supplemental Figure 7A). Consistent with the ability of distinct HSPs to assemble in higher order structures termed “epichaperomes” (40, 41), the p62 complexes in CD23-*Rock1* B cells stimulated with TLR9-L ± heme also contained HSP90 (Figure 7A and Supplemental Figure 7A). Thus, in the presence of pathogen-associated stressors, absence of ROCK1 results in the formation of p62 complexes containing ubiquitinated proteins and HSPs that can promote the oligomerization and phase separation of p62.

We reasoned that exposure to intense damage may require differentiating B cells to engage a unique machinery to handle the increased stress. EIF2AK1/HRI (hereafter termed HRI), 1 of the 4 eIF2α kinases, regulates the integrated stress response (ISR) in response to heme deprivation and several other stressors (42, 43). HRI can also restrain the accumulation of protein aggregates and interact with HSPs, and dissociation of HRI from HSPs is critical for its role in proteostasis (44, 45). We thus explored the possibility that ROCK1 might control HRI. We first investigated whether the presence of HSPs within the p62 complexes in CD23-*Rock1* B cells was accompanied by aberrant recruitment of HRI. HRI was present in the p62 complexes formed in the absence of ROCK1 only under conditions that also resulted in the recruitment of HSPs (Figure 7B and Supplemental Figure 7B). Since HRI activity can also be controlled by heme binding, which can inhibit the ability of HRI to engage the ISR (42, 43), we also assessed eIF2α phosphorylation and ATF4 expression (Figure 7C and Supplemental Figure 7C). Compared with WT B cells, both p-eIF2α and ATF4 levels were diminished in CD23-*Rock1* B cells stimulated with a TLR9-L and heme. No differences were instead observed in levels of XBP1s (Supplemental Figure 7D). Taken together, these results suggest that lack of B cell ROCK1 leads to the inappropriate recruitment of HRI to p62 complexes and modifies the ability of HRI to respond to heme and engage the ISR.

HRI undergoes extensive phosphorylation, which regulates its intramolecular interactions, its activity, and its sensitivity to heme (46). To investigate whether ROCK1 might modulate HRI by regulating its phosphorylation, we transfected FLAG-tagged HRI in 293T cells, immunoprecipitated it, and performed IVKs with CA-ROCK1. Incubation with CA-ROCK1 resulted in the appearance of a slower mobility form of HRI (Figure 7D) consistent with previous studies showing changes in HRI mobility upon its phosphorylation (46). To assess whether ROCK1 could affect HRI interaction with HSPs and response to heme, we transfected FLAG-tagged HRI in 293T cells, stimulated the cells ± heme, and assessed the ability of immunoprecipitated FLAG-tagged HRI to interact with HSP90 after performing IVKs with CA-ROCK1 (Figure 7E). IVKs with CA-ROCK1 resulted in a marked decrease in the association of HRI with HSP90 in the presence of heme. Immunoprecipitated HRI incubated in the presence/absence of CA-ROCK1 and subjected to mass spectrometry analysis, furthermore, showed increased phosphorylation at S5 and S144 located in the heme-binding domain and S293 located in the kinase insert region (47, 48) (Figure 7F). To verify these findings, we mutated S5, S144, and S293 in HRI to alanine (HRI-AAA). HRI-AAA lost the ability to be phosphorylated by CA-ROCK1 (Supplemental Figure 7E). Importantly, these mutations prevented the ability of CA-ROCK1 to mediate the dissociation of HRI from HSP90 in the presence of heme, supporting the functional relevance of these sites (Figure 7G). These results thus suggest that ROCK1 can phosphorylate HRI and control the interplay of HRI with HSP90.

Release of HSPB8 from HRI is critical to prevent formation of toxic protein aggregates (44). We thus hypothesized that the dysregulated recruitment of both HRI and HSP90 to p62 in ROCK1-deficient B cells might promote the formation of p62 aggregates and that this, in turn, could be due to the ability of HSP90 to mediate the assembly of epichaperome-like complexes. We thus assessed whether PU-H71, an HSP90 inhibitor that functions as an “epichaperome” disruptor (41), could affect the recruitment of key components to the p62 complexes. Addition of PU-H71 to CD23-*Rock1* B cells stimulated with TLR9-L ± heme markedly decreased the association of p62 with raptor, RIPK3, and TDP-43 as early as 6 hours after incubation without affecting the expression levels of these proteins (Figure 7H and Supplemental Figure 7F). Interaction of p62 with HSP90 was also decreased while its association with BiP was largely unaffected (Figure 7H and Supplemental Figure 7F). Diminished recruitment of raptor to p62 upon PU-H71 treatment, furthermore, ameliorated the aberrant phosphorylation of 4EBP1 observed in the absence of ROCK1 (Figure 7I). Taken together these studies suggest that the ability of ROCK1 to regulate the association of HSP90 with HRI can limit the assembly of distinctive p62 complexes that sequester active mTORC1, pro-inflammatory kinases, and key regulators of RNA metabolism and protein quality control.

## Discussion

**Figure 8:**
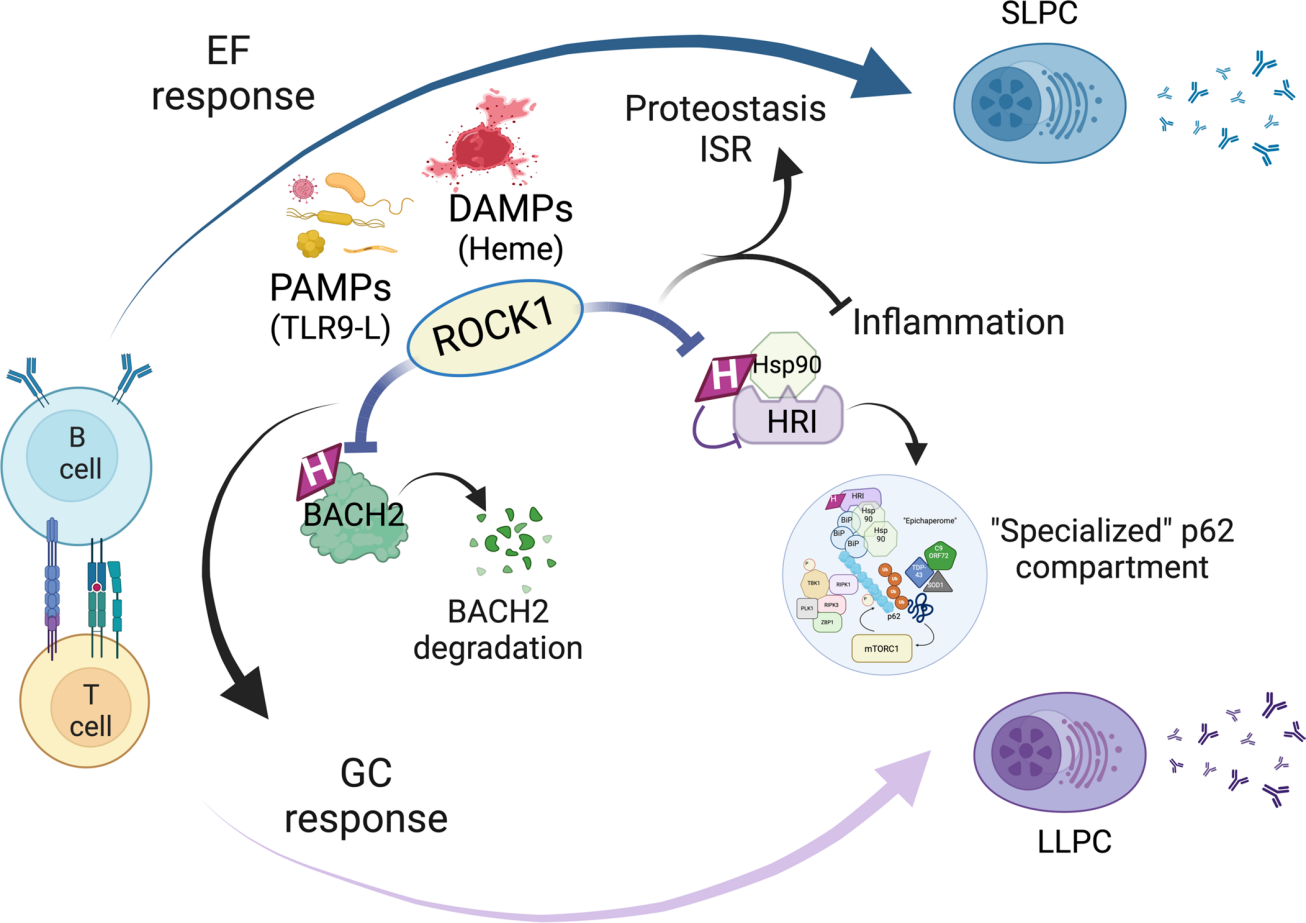
Schematic model summarizing the role of ROCK1 in B cells exposed to hostile conditions. ROCK1 can help orchestrate B cell differentiation in settings replete with PAMPs and DAMPs like heme. In the absence of ROCK1, activated B cells are impaired in their ability to undergo GC differentiation due to increased degradation of BACH2. ROCK1-deficient B cells differentiating into short-lived plasma cells or long-lived plasma cells (SLPCs or LLPCs), furthermore, cannot limit their pro-inflammatory capabilities and maintain optimal antibody production because of the inability of HRI to dissociate from HSP90, restrain the assembly of “specialized” p62 compartments, maintain proteostasis, and engage additional stress responses. Created in BioRender (l44d648; https://BioRender.com/l44d648).

Protective humoral immunity requires that B cells successfully execute their differentiation programs under a wide range of potentially damaging conditions. We show that B cell ROCK1 acts as a critical regulatory hub that enables B cells to implement molecular and biochemical programs necessary for their optimal activation and differentiation when faced with hostile conditions. In these settings, B cell ROCK1 unexpectedly restrains mTORC1 signaling and pro-inflammatory capabilities and helps coordinate stress responses. ROCK1 regulates these processes by modulating 2 heme-regulated proteins that exert key roles in B cell differentiation and stress responses, BACH2 and HRI. Engagement of HRI could be particularly important in stressful settings, since it could operate in B cells that differentiate via either an EF- or a GC-dependent route and be broadly required to help maintain adequate antibody secretion. Taken together our studies support a model whereby regulation of BACH2 and HRI by ROCK1 (Figure 8) can provide a potentially novel defense strategy to help counteract the ability of severe pathogens like *Plasmodium* to exploit hemolysis and tissue damage to avoid productive immune responses.

Together with our previous work (21, 22), the present study shows that both ROCK1 and ROCK2 can be activated in response to B cell receptor and CD40 engagement and that, in immunization models, the 2 ROCKs partially complement each other by controlling cholesterol biosynthesis and promoting GC formation. The experimental malaria model, however, demonstrates that the activity of the 2 ROCKs can be dynamically regulated by the presence of pathogen-driven signals and affect their ability to compensate for each other. During this infection, ROCK1 was the primary ROCK family member activated in B cells, and its deletion alone resulted in profound pathology. In addition to reprogramming B cell metabolism, here we show that ROCK1 can also impact B cell differentiation by phosphorylating BACH2 and regulating its activity in a complex manner. ROCK1 can indeed phosphorylate BACH2 in its heme-binding domain and prevent the heme-driven degradation of BACH2, thus helping protect BACH2 in settings marked by increased hemolysis, such as malaria and some autoimmune diseases. ROCK1 deficiency, however, also resulted in the upregulation of a subset of BACH2-repressed targets even in B cells stimulated in the absence of heme. This effect was likely linked to the ability of ROCK1 to phosphorylate BACH2 near the bZIP domain, a site that could affect its DNA-binding activity or interaction with other transcriptional activators. ROCK1 could thus differentially modulate the repressive capabilities of BACH2 depending on the signals to which B cells are exposed.

Absence of ROCK1 in B cells was also accompanied by an enhanced pro-inflammatory profile and an inability to maintain antibody production. An investigation into the mechanisms underlying these findings uncovered an unexpected role for ROCK1 in regulating HRI, a key controller of proteotoxicity and the ISR. Interestingly, exposure of CD23-*Rock1* B cells to TLR9 ligands alone was sufficient to drive the assembly of p62 complexes while defects in the upregulation of ATF4 were observed only upon exposure to both TLR9 engagement and heme, supporting the idea that these 2 functional outputs of HRI can be differentially controlled. Such multifaceted control may enable B cells to harness the beneficial effects of HRI on proteostasis under a wide range of settings but engage the ISR in only very hostile environments. Phosphorylation of HRI by ROCK1 could also be important in allowing B cells, in contrast with erythroid cells, to upregulate rather than inhibit the ISR in the presence of high levels of heme. At a mechanistic level, we found that ROCK1 controls the association of HRI with HSP90 and prevents the dysregulated recruitment of both HRI and HSP90 to p62 complexes. Consistent with the ability of HSP90 to assemble in epichaperomes in response to intense stress, the p62 complexes in CD23-*Rock1* B cells also contained BiP. Furthermore, PU-H71, an inhibitor that specifically recognizes epichaperome-bound HSP90, interfered with the recruitment of HSP90, raptor, RIPK3, and TDP-43 to the p62 complexes while only minimally affecting the association of p62 with BiP. Taken together, these findings support a model (Supplemental Figure 8) whereby, in the presence of stressors like PAMPs and DAMPs, lack of ROCK1 prevents the disassembly of HRI from HSP90 facilitating the interaction of HSP90 with p62-associated BiP, the subsequent formation of epichaperome-like complexes, and the stabilization of a distinctive subset of p62 compartments.

The dysregulated assembly of p62 complexes observed in ROCK1-deficient B cells was linked to the inability of these cells to properly regulate the activity of mTORC1, a step required for the successful execution of the terminal B cell differentiation program. Indeed, in the absence of ROCK1, both raptor and TRAF6 were aberrantly recruited to p62 resulting in the persistent activation of a localized pool of mTORC1. The range of mTORC1 substrates whose phosphorylation status was altered in ROCK1-deficient B cells was limited to a selected subset of proteins that included 4EBP1, suggesting that this pool of mTORC1 is primarily involved in the control of eIF4E activity and the translation of eIF4E-sensitive transcripts, such as AICDA (49, 50). The mTORC1 targets affected by the absence of ROCK1 also included a site in p62, whose phosphorylation resulted in the increased association of p62 with Keap1, a step known to promote the phase separation of p62 (38, 51), and addition of rapamycin was able to disrupt the p62 complexes formed in the absence of ROCK1 (Supplemental Figure 7G). These findings suggest that, once recruited to these p62 complexes, dysregulated mTORC1 activity can further fuel formation of the p62 condensates amplifying these abnormalities.

Aberrant formation of p62 complexes in ROCK1-deficient B cells was also connected to the acquisition of an enhanced pro-inflammatory profile. Consistent with the ability of p62 to interact with RIPK1, the p62 complexes formed in the absence of ROCK1 contained RIPK1 as well as RIPK3 and ZBP1, key ripoptosome components implicated in the orchestration of inflammatory responses and necroptosis in innate cells but whose role in B cells is largely unexplored. No increases in the cleavage of MLKL or cell death were observed, suggesting that, under these conditions, these ripoptosome-like complexes primarily mediate a pro-inflammatory rather than a necroptotic role. Presence of TBK1 in the complexes likely accounts for this shift given the known ability of TBK1 to suppress RIPK1-induced cell death (52, 53). While assembly of these complexes in the absence of ROCK1 was unexpected, such a response could be important against severe pathogens that disarm RhoA, the key upstream activator of ROCK1. Assembly of ripoptosome-like complexes skewed toward inflammation rather than death could indeed be advantageous in the B cell compartment, since it could avoid the potential elimination of pathogen-specific B cells while enabling B cells to function, at least for the short term, as additional effectors in response to severe infections.

Lack of ROCK1 also resulted in the recruitment of PLK1 to these p62 complexes and was accompanied by upregulation of signatures related to the G2/M phase of the cell cycle in vivo and in vitro. These findings are consistent with studies showing that PLK1 activity can be regulated by its sequestration into “mitotic ripoptosomes” (34, 35). Presence of PLK1 in these complexes could allow B cells to tightly adjust PLK1 activity in highly inflammatory and stressful settings and be poised to halt mitosis to avoid the emergence of B cell progeny with damaging chromosomal alterations. Interestingly, p62^+^ aggresomes such as those formed in the absence of HRI have been reported to be perinuclear (45, 54), and ROCK1 can localize to the nucleus (55). Given that our phospho-proteomic analysis revealed that absence of ROCK1 affects the phosphorylation of key components involved in the DNA damage response, such as 53BP1 (56), ROCK1-controlled mechanisms may enable B cells to closely coordinate cell cycle checkpoints and DNA repair to handle the increased levels of DNA damage that they may incur when differentiating under hostile conditions.

Our studies thus support a role for ROCK1 as a critical regulatory hub that enables activated B cells to integrate information provided by key B cell activation signals with cues regarding the presence of a pathogen and the extent of the surrounding damage. Loss of ROCK1 activity due to disabling of RhoA by severe pathogens (18) may be the ultimate signal to differentiating B cells of extreme stress, leading them to assemble “specialized” p62 compartments. This could allow for more efficient signaling, enhanced protective antioxidant/stress responses, and maintenance of selected functional capabilities to ensure that the high bioenergetic demands of antibody production are closely matched to available resources. If these compartments are inappropriately assembled or disassembled, however, such emergency responses could become maladaptive. Interestingly, phospho-proteomic alterations could be observed even under stimulatory conditions where only minimal, if any, formation of p62 aggregates could be detected biochemically, suggesting that these p62 aggregates also assemble under those conditions but that they are unstable and that presence of pathogen-associated stressors drives their further maturation or stabilization. Given that HRI activity is also controlled by stressors such as oxidative and mitochondrial stress (42, 43), these pathways could be engaged by B cells to endure adverse conditions other than hemolysis and be utilized by long-lived PCs and MBCs to handle sudden disturbances in their niches. Employment of ROCK1 to dynamically adapt the molecular machinery of B cells to withstand pathogen-associated and environmental stressors could thus have broad relevance for infections, vaccine development, autoimmunity, and even malignancies.

While extending our in vitro studies to in vivo settings will be critical to help extrapolate these results to human conditions, the surprising role of ROCK1 in limiting the assembly of p62 complexes and restraining mTORC1 activation and inflammatory responses highlights the challenges of therapeutically targeting these kinases, a feat that is being undertaken for several age-related disorders, including ALS (57, 58). Our findings may indeed also be relevant for other cells that have high bioenergetic demands rendering them more vulnerable to environmental insults, like neurons. This notion is indeed supported by the remarkable concentration of ALS-associated machinery in the p62 complexes formed in the absence of ROCK1, suggesting that these complexes represent a point of convergence for fundamental pathways involved in DNA damage, RNA handling, protein quality control, and oxidative stress. Understanding how relocalization of critical kinases to these “specialized” p62 compartments can affect their interrelationships and their sensitivity to inhibitors could also be critical for successful therapeutic interventions in a broad range of diseases. Thus, our studies identify a fundamental mechanistic framework where rapid changes in ROCK1 activity can help coordinate critical cellular programs to ensure organized, efficient decision-making when faced with sudden and potentially lethal pathogenic and damaging challenges.

## Methods

### Sex as a biological variable.

Female mice between 6 and 12 weeks of age were used in in vivo experiments. Only female mice were used in in vivo studies to facilitate future extrapolation of the results to autoimmune disorders, which primarily affect females. The findings should, however, be applicable to both sexes. Both males and females were used in in vitro experiments and gave similar findings.

### Mice.

All mice were on a C57BL/6 background. The generation of Rock1^fl/fl^ was previously described (59). CD23-cre mice were provided by Jayanta Chaudhuri (Memorial Sloan Kettering Cancer Center) and were previously described (21). B6.129P2(Cg)-*Ighg1^tm1(Cre)Cgn^*/J (Cγ1-Cre) mice were purchased from Jackson Laboratory. BLIMP1-YFP reporter mice were previously described (60). All mice used in the experiments were kept under specific pathogen–free conditions.

### Materials and analytical procedures.

The detailed description of analytical procedures and materials (including densitometry analysis of Western blots and representative FACS gating) can be found in Supplemental Methods.

### Statistics.

All plots show data points from independent mice pooled across multiple experiments, unless otherwise noted. *P* values were calculated with 2-tailed *t* tests or 2-way ANOVA followed by multigroup comparisons, as indicated in the figure legends. A *P* value less than 0.05 was considered statistically significant. Statistical analysis was performed with GraphPad Prism 8.

### Study approval.

All animal experiments were approved by the Institutional Animal Care and Use Committee of the Hospital for Special Surgery and Weill Cornell Medicine/Memorial Sloan Kettering Cancer Center, and the experiments were carried out following these established guidelines.

### Data availability.

The public dataset that supports the RNA-Seq findings of this study is available in the NCBI Gene Expression Omnibus under accession code GSE237201. The TMT proteomics data have been deposited to the ProteomeXchange Consortium via the PRIDE partner repository with the dataset identifier PXD059659. Values for all data points in graphs are reported in the Supporting Data Values file. The datasets generated during or analyzed during the studies reported herein do not make use of custom code or mathematical algorithms central to the conclusions.

## Author contributions

JRC designed and performed the experiments, interpreted the experiments, and wrote the manuscript; ER, SG, DFC, DJ, SPP, and SV performed the experiments; TP assisted with the histological analyses; YBK provided the *Rock1^fl/fl^* mice; MMM and ZL performed and analyzed all the mass spectrometry and proteomics; EG analyzed the RNA-Seq experiments; YC helped analyze the RNA-Seq data; NZ and LC helped write the manuscript; and ABP designed and supervised the study, interpreted the experiments, and wrote the manuscript.

## Supplementary Material

Supplemental data

Unedited blot and gel images

Supporting data values

## Version 1 02/04/2025

In-Press Preview

## Version 2 03/10/2025

Electronic publication
