# Supplementary material for: ROCK1 promotes B cell differentiation and proteostasis under stress through the heme-regulated proteins, BACH2 and HRI
Source: JCI Insight. 2025 Feb 4;10(5):e180507. doi: 10.1172/jci.insight.180507 (PMC11949073; doi:10.1172/jci.insight.180507)

SUPPLEMENTAL MATERIALS

METHODS

Antibodies and flow cytometry. The following monoclonal antibodies were used for flow cytometry: B220-PB or B220-APC/ Cy7 (RA3-6B2; 1:400), CD3-PE (145-2C11; 1:800), CD4-APC (RM4-5; 1:400), CD8-A700 (53-6.7; 1:200), CD11b-PE/Cy7 or CD11b-FITC (M1/70; 1:400), CD11c-APC/Cy7 or CD11c-APC (N418; 1:400), CD19-PB or CD19-PE (HIB19; 1:400), CD21-APC (7E9; 1:200), CD23-PE or CD23-PerCP/Cy5.5 (B3B4; 1:200), CD44-PCP or CD44-A700 (IM7; 1:200), IFNγ-A488 (XMG1.2, 1:400), and Tbet-PE (4B10; 1:800) were obtained from BioLegend. Streptavidin-conjugated antibodies were also obtained from BioLegend. Antibodies to CD138-APC (281-2; 1:1200), CXCR5-Biotin (2G8; 1:200), Fas-Biotin (Jo2; 1:200),and GL7-FITC (1:600) were obtained from BD. Antibodies to Foxp3-APC (FJK-16s; 1:100), IgD-FITC (11-26; 1:500), IgM-PE/Cy7 (II/41; 1:1000), and PD1-FITC (J43; 1:200) were obtained from eBioscience. For intracellular staining, cells were fixed after surface staining at 4 °C with the Transcription Factor Staining Kit (eBioscience; #00-5523-00). For intracellular cytokine staining, splenocytes were stimulated with 50μg/mL PMA and 1μM Ionomycin for 4 hr. Cells were incubated with Brefeldin A for the final 3 h of stimulation. After stimulation, cells were fixed and permeabilized with a Transcription Factor Staining Kit (eBioscience; #00-5523-00) and stained using anti-IFNγ-APC (BioLegend; XMG1.2; 1:200) and recombinant mouse IL21R Fc Chimera (R&D; Cat # 596-MR-100; 1:600) followed by PE-labeled affinity-purified F(ab')2 fragment of goat anti-human Fcγ (Cat # 109-116-170, Jackson ImmunoResearch). All data were acquired on a BD FACS Canto and analyzed with FlowJo (TreeStar) software.

Immunizations and experimental malaria. Mice were immunized intraperitoneally (ip) with 100 μg NP30–40-CGG in alum 0 to 28 days before analysis. JH4 sequencing was performed as described (1). To start blood-stage infections (BSL1), mice were injected ip with 1 × 10^6 infected RBCs per mouse of the nonlethal strain *Plasmodium yoelii* 17XNL resuspended in RPMI 1640 medium as described (2). Specifically, LD-column (Miltenyi) purified *P. yoelii*-infected lysates (10^6 infected erythrocytes/mL) were prepared by freeze-thawing 10 times. Mice were euthanized at the indicated days by CO_2 asphyxiation and a secondary method as recommended by the Panel on Euthanasia of the American Veterinary Medical Association. To evaluate parasitemia, thin blood smears were made by bleeding mice from a nick in the tail. Smears were stained with KaryoMAX Giemsa (Life Technologies, Norwalk, CT), and a minimum of 500 RBCs per smear were counted. To evaluate anemia and other hematologic parameters, blood samples were submitted to the Laboratory of Comparative Medicine at WCM/MSKCC.

Cell sorting. For cell sorting, single-cell suspensions from pooled spleens were pre-enriched for B cells with biotinylated anti-B220 and streptavidin microbeads and B cells stained with B220 (Biolegend; RA3-6B2), CD23 (Biolegend; B3B4), CD38 (Biolegend; 90), and GL7 (Biolegend; GL7) for the immunization experiments or whole splenocytes stained with B220 (Biolegend; RA3-6B2), CD19 (Biolegend; 6D5), CD138 (BD Bioscience, 281-2), and IgD (eBioscience, 11-26c) for the malarial infection experiments. In the case of the TD immunizations spleens were pooled for sorting while B cell populations from individual spleens were sorted for the experimental malaria experiments. Samples were sorted on either a BD FACS Aria II or a BD Influx.

ELISAs and ELISPOTs. For the total Ig ELISA, plates were coated with 10 µg/mL goat anti–mouse Ig at 4°C overnight and blocked in 1% BSA in PBS at RT for 1 hour. For the NP-specific Ig ELISA, plates were coated with 50 µg/mL NP-BSA conjugated at the appropriate ratio at 4°C overnight and blocked in 2% BSA in PBS at RT for 1 hour. Sera were diluted at various ratios and incubated on coated plates at 25°C for 2 hours. Plates were then incubated with either alkaline phosphate–labeled or HRP-labeled goat anti–mouse IgM (Southern Biotech; Cat#1020-04), IgG1 (Southern Biotech; Cat#1070-04), IgG2c (Southern Biotech; Cat#1079-04), or IgA (Southern Biotech; Cat#1040-04), Fc antibody for 1 hour before development. For anti-malaria antibody ELISAs, NUNC Immuno Microwell 96-wellplates (Thermo) were coated with 1:400 LD-column (Miltenyi) purified *P. yoelii*-infected lysates at 10^6 parasites/ µL at 37°C overnight and blocked in 2% BSA in PBS at room temperature for 2 h. The mean OD at 450 nm from replicate wells was compared with the same dilution of a reference positive serum to calculate relative units (RU) as described (2). For anti-cardiolipin and anti-phosphatidylserine ELISA, Immulon 2HB plates (Thermo) were coated with 75µg/mL of cardiolipin or with 30 µg/mL phosphatidylserine dissolved in 100% ethanol overnight. Sera were diluted 1:200 and incubated on coated plates at 25 °C for 2 hrs. Plates were then incubated with HRP-labeled goat anti-mouse IgM antibody for 1 h before development. OD450 was measured on a microplate reader. For ELISPOT assays, plates were coated overnight at 4°C with 100 µg/mL goat anti–mouse Ig for detection of total Ig ASCs or 50 µg/mL NP-BSA conjugated at the appropriate ratio for detection of NP-specific Ig ASCs. Nonspecific binding was blocked with 3% BSA and 5% FBS in PBS, and samples were incubated at 37°C for 2 hours. Antibodies conjugated to biotin (goat anti–mouse IgG or goat anti–mouse IgM) were added and incubated overnight at 37°C followed by streptavidin-alkaline-phosphate and detection using 5-bromo-4- chloro-3-indolyl phosphate (BCIP).

Histology. Tissue specimens were fixed in 10% neutral buffered formalin and embedded in paraffin. Tissue sections were stained with periodic acid-Schiff (PAS) or with hematoxylin and eosin (H&E) and analyzed by light microscopy. The histological scoring system was adapted from published studies (3, 4) on malaria-associated pathology in patients infected with *Plasmodium*

falciparum. Specimens were captured by Q capture software on a Nikon Eclipse microscope and quantifications were calculated using ImageJ software.

RT-qPCR and DNA constructs. Total RNA was isolated using the RNeasy Plus Mini Kit (QIAGEN). cDNAs were prepared using the iScript cDNA synthesis kit. Real-time PCR was performed using the iTaq Universal SYBR Green Supermix. Gene expression was calculated using the ΔΔCt method and normalized to Cyclophilin A (murine *Ppia* Forward: 5′-TTGCCATTCCTGGACCCAAA-3′, murine *Ppia* Reverse: 5′-ATGGCACTGGCGGCAGGTCC-3′). RT-qPCR primers for *Rock1*, *Rock2*, *Prdm1* (BLIMP1) and *Bach2* were obtained from Qiagen. FLAG-tagged mouse Bach2 expression construct in pcDNA expression vector was a kind gift from Ari Melnick (WCM, NY). Point mutants of FLAG-Bach2 were generated by PCR and confirmed by DNA sequencing. FLAG-tagged mouse HRI expression construct in pMXs Retroviral expression vector was obtained from Addgene (Plasmid # 101791). HA-tagged mouse HRI expression construct in pCMV3-C-HA expression vector was obtained from Sino Biologicals (Cat# MG50852-CY). Point mutants of HA-HRI were generated by PCR and confirmed by DNA sequencing. Additional primers used in RT-qPCR are listed in Suppl. Table 5.

RNA sequencing*.* The quality of all RNA and library preparations was evaluated with BioAnalyzer 2100 (Agilent Technologies). Sequencing libraries were sequenced by the Epigenomics Core Facility at Weill Cornell Medicine using a HiSeq 2500, 50-bp paired-end reads at a depth of approximately 22 to 30 million reads per sample. Read quality was assessed and adaptors trimmed using FASTP (58). Reads were then mapped to the mouse genome (mm10) and reads in exons were counted against Gencode v27 with STAR2.6 Aligner (59). Differential gene expression analysis was performed in R using edgeR 3.24.3. Genes with low expression levels (<2 counts per million in at least 1 group) were filtered from all downstream analyses. Replica-associated batch correction was performed by directly incorporating a batch-specific term into a linear model. Differential expression was estimated using a quasi-likelihood framework. The Benhamini-Hochberg FDR procedure was used to correct for multiple testing. Genes with an unadjusted *P* value of less than 0.01 were considered differentially expressed. Downstream analyses were performed in R using a visualization platform build with Shiny developed by bioinformaticians at the David Z. Rosensweig Genomics Research Center at the HSS. GSEA was performed using GSEA software (Broad Institute)[59]. Genes were ranked (Signal2Noise metric) by the difference of log-transformed counts per million for contrasted conditions. The Molecular Signatures Data-Base, version 62 (Broad Institute) was used as a source of gene sets with defined functional relevance. Gene sets ranging between 15 and 1000 genes were included in the analysis. Nominal *P* values were FDR corrected, and gene sets with an FDR $\leq$0.1 were used to create GSEA enrichment plots.

Cell cultures and transfections. $CD23^+$ B cells were purified from single cell suspensions of splenocytes with biotinylated anti-CD23 (BD Bioscience; Cat#553137) and streptavidin microbeads (Miltenyi Biotec; Cat#130-048-101) as described (5). Cells were cultured for 3 d in RPMI 1640 medium (Corning) supplemented with 10% FBS, 100 U/mL Penicillin, 100μg/mL Streptomycin, non-essential amino acids (Corning), 2 mM L-Glutamine (Corning), 25 mM HEPES (pH 7.2–7.6), and 50 μM β-Mercaptoethanol and stimulated with 5 μg/mL F(ab')2 anti-mouse IgM (Jackson ImmunoResearch, Cat# 715-006-020); 5 μg/mL purified anti-mouse CD40 (BioXcell; BP0016-2), a TLR9 ligand (TLR9-L, CpG ODN 1668, 1mg/ml) (Invivogen, Cat#tlrl-1668-1) and hemin (heme, 60mM; Sigma-Aldrich, Cat#H9039) in various combinations. In selected experiments LPS was added at 25mg/mL for three days (Invivogen, Cat#tlrl-3pelps). For autophagy assays BafilomycinA1 (50-100 nM, Sigma-Aldrich, Cat#B1793) was added for the last 4 hrs of culture. Cycloheximide (100 mg/ml, Sigma-Aldrich, Cat# C4859) was added for the last 3-6 hrs of culture. PU-H71 (1mM) was added for the last 6-24 hrs of culture. Rapamycin (20 nM, Cell Signaling Technology, Cat # 9904) was added for the last 20 hrs of a 3 d culture. 293T cells (ATCC, CRL-3216) were grown in DMEM with 10% FBS, 100 U/mL penicillin, 100μg/mL Streptomycin and transfected using the Mirus Transfection Kit with expression constructs for Flag-Bach2, or Flag-Bach2 mutants, or FLAG-HRI, or HA-HRI or HA-HRI mutants.

Immunoblot analysis, kinase activity assays, and immunoprecipitations. Nuclear and cytoplasmic extracts were prepared with NE-PER Nuclear and Cytoplasmic Extraction Reagents (Pierce). Whole cell extracts were prepared as previously described (1). Extracts were immunoprecipitated with anti-p62 (Cell Signaling Technology, Cat #39749) antibody. Anti-Flag monoclonal antibody M2 conjugated with horseradish peroxidase (HRP) was obtained from Sigma-Aldrich, (Cat#A8592). For ROCK kinase activity assays, ROCK1 or ROCK2 was immunoprecipitated from whole cell extracts using anti-ROCK1 (Cell Signaling, Cat#4035) or anti-ROCK2 (Cell Signaling, Cat#9029) antibodies as described previously (1) and quantifications were calculated using ImageJ software. ROCK1-mediated phosphorylation of BACH2 and HRI was assessed by *in vitro* ROCK1 kinase assays using active recombinant ROCK1 protein (Abcam, Cat# ab51415) and immunoprecipitated Flag-tagged BACH2 protein or FLAG-tagged HRI protein or HA-tagged HRI protein. Briefly, immunoprecipitated BACH2 or HRI was incubated with 400 ng purified active ROCK1 in kinase buffer (25 mM Tris, pH 7.5, 10 mM $MgCl_2$, 5 mM β-glycerolphosphate, 0.1 mM Na_3VO_4, and 2 mM DTT) containing 0.2mM ATP for 60 minutes at 30°C. The kinase reactions were terminated by washing the beads with 25 mM Tris, pH7.5 and then heating in SDS-PAGE sample buffer. The reactions products were resolved on a 8% SDS-PAGE gel followed by detection of phosphorylated Bach2 products using a Phospho-Ser/Thr (PKA Substrate) Ab (Cell Signaling Technology, Cat # 9621), which recognizes a consensus site

similar to that of ROCK1. ROCK1-mediated phosphorylation of FLAG-HRI or HA-HRI was detected by mobility shift by immunoblotting with anti-FLAG mAb (M2)-HRPO (Sigma, Cat#A8952) or anti-HA mAb (Roche, Cat # 12013819001)-HRPO, respectively. Noncontiguous lanes run on the same gel are separated by black lines in the figures. Densitometric analysis was performed using ImageJ processing software. Additional antibodies used in Western blotting and immunoprecipitations are listed in Suppl. Table 6.

LC-MS/MS and proteomic data analysis.

Primary B cells phospho-proteomics: Cell pellets were lysed with buffer containing 8 M urea and 200 mM EPPS (pH at 8.5) with protease inhibitor (Roche) and phosphatase inhibitor cocktails 2 and 3 (Sigma). Benzonase (Millipore) was added to a concentration of 50u/mL and incubated (RT, 15 min) followed by water bath sonication. Samples were centrifuged at 4°C, 14,000 g's for 10 min and supernatant extracted. The Pierce bicinchoninic acid (BCA) protein concentration assay was used for determining protein concentration. Protein disulfide bonds were reduced with 5 mM tris (2-carboxyethyl) phosphine (room temperature, 15 min), then alkylated with 10 mM iodoacetamide (RT, 30 min, dark). The reaction was quenched with 10 mM dithiothreitol (RT, 15 min). Aliquots of 100 ug were taken for each sample and diluted to approximately 100 µL with lysis buffer. Samples were subject to chloroform/methanol precipitation as previously described[1]. Pellets were reconstituted in 200mM EPPS buffer and digested with Lys-C (1:50 enzyme-to-protein ratio) and trypsin (1:50 enzyme-to-protein ratio) at 37°C overnight. Peptides were TMT-labeled as described (6). Briefly, peptides were TMT-tagged by addition of anhydrous ACN and TMTPro reagents (16plex) for each respective sample and incubated for 1 hr (RT). A ratio check was performed by taking a 1 µL aliquot from each sample and desalted by StageTip method (7). TMT-tags were then quenched with hydroxylamine to a final concentration of 0.3% for 15 min (RT). Samples were pooled 1:1 based on the ratio check and vacuum-centrifuged to dryness. Dried peptides were reconstituted in 1mL of 3% ACN/1% TFA, desalted using a 100mg tC18 SepPak (Waters), and vacuum-centrifuged overnight. Phosphopeptides were enriched using the Thermo High-Select Fe-NTA Phosphopeptide Enrichment Kit (Cat. No.: A32992). The phosphopeptide elute was vacuum centrifuged to dryness and reconstituted in 100 µL of 1% ACN/25mM ammonium bicarbonate (ABC). A StageTip was constructed by placing two plugs with a narrow bore syringe of a C18 disk (3M Empore Solid Phase Extraction Disk, #2315) into a 200 µL tip (VWR, Cat. No.: 89079-458). StageTips were conditioned with 100 µL of 100% ACN, 70% ACN/25mM ABC, then 1% ACN/25mM ABC. Phospho-enriched sample was loaded onto the StageTip and eluted into 6 fractions of 3, 5, 8, 10, 12, and 70% ACN/25mM ABC with 100 µL each. Fractions were immediately dried down by vacuum-centrifugation and reconstituted in 0.1% formic acid (FA) for LC-MS/MS.

Phospho-depleted peptides were centrifuged to dryness and reconstituted in 1 mL of 1% ACN/25mM ABC. Peptides were fractionated into 48 fractions. Briefly, an Ultimate 3000 HPLC (Dionex) coupled to an Ultimate 3000 Fraction Collector using a Waters XBridge BEH130 C18 column (3.5 um 4.6 x 250 mm) was operated at 1 mL/min. Buffer A, B, and C consisted of 100% water, 100% ACN, and 25mM ABC, respectively. The fractionation gradient operated as follows: 1% B to 5% B in 1 min, 5% B to 35% B in 61 min, 35% B to 60% B in 5 min, 60% B to 70% B in 3 min, 70% B to 1% B in 10min, with 10% C the entire gradient to maintain pH. The 48 fractions were then concatenated to 12 fractions, (i.e. fractions 1, 13, 25, 37 were pooled, followed by fractions 2, 14, 26, 38, etc.) so that every 12th fraction was used to pool. Pooled fractions were vacuum-centrifuged then reconstituted in 1% ACN/0.1% FA for LC-MS/MS.

Phosphopeptide-enriched and phospho-depleted peptide fractions were analyzed by LC-MS/MS using a Thermo Easy-nLC 1200 (Thermo Fisher Scientific) with a 50 cm (inner diameter 75µm) EASY-Spray Column (PepMap RSLC, C18, 2µm, 100Å) heated to 60°C coupled to a Orbitrap Fusion Lumos Tribrid Mass Spectrometer (Thermo Fisher Scientific). Peptides were separated at a flow rate of 300nL/min using a linear gradient of 1 to 35% acetonitrile (0.1% FA) in water (0.1% FA) over 4 hours and analyzed by SPS-MS3. MS1 scans were acquired over a range of m/z 375-1500, 120 K resolution, AGC target (standard), and maximum IT of 50 ms. MS2 scans were acquired on MS1 scans of charge 2-7 using an isolation of 0.7 m/z, collision induced dissociation with activation of 32%, turbo scan and max IT of 50 ms. MS3 scans were acquired using specific precursor selection (SPS) of 10 isolation notches, m/z range 100-1000, 50K resolution, AGC target (custom, 200%), HCD activation of 45%, and max IT of 150ms. The dynamic exclusion was set at 60s.

In vitro BACH2 and HRI phosphorylation: Recombinant BACH2: Immunoprecipitated BACH2 was incubated with 400 ng purified active ROCK1 in kinase buffer (25 mM Tris, pH 7.5, 10 mM MgCl2, 5 mM β-glycerolphosphate, 0.1 mM Na3VO4, and 2 mM DTT) containing 0.2mM ATP for 60 minutes at 30°C. The kinase reactions were terminated by washing the beads with 25 mM Tris, pH7.5 and then with 50mM EPPS (pH 8.5). Supernatant was removed and trypsin/Lys-C in 50mM EPPS was added (1:100 ratio) and digested overnight at 37°C. An additional equal amount of trypsin/LysC was added and digested for 4 hours at 37°C. Samples were spun down, transferred to fresh Eppendorf tubes, and anhydrous acetonitrile (ACN) was added to each. Samples were TMT-labeled as described(6). Briefly, samples were TMT-tagged by adding 4µL (28ug/µL) TMTPro reagents for each respective sample and incubated for 1hr (RT). TMT-tags were then quenched with hydroxylamine to a final concentration of 0.3% for 15 min (RT). Samples were pooled in their entirety then vacuum-centrifuged to dryness. Dried sample was reconstituted in 300µL 0.1% TFA and pH confirmed (adjusted when needed to acidic condition). The Pierce™

High pH Reversed-Phase Peptide Fractionation Kit (Cat. No.: 84868) was used to fractionate the pooled TMT sample into 8 fractions following manufacturer's instructions. The 8 fractions were concatenated to 4 fractions (i.e. fractions 1 and 5 pooled, 2 and 6, etc.) and vacuum-centrifuged to dryness. Fractions were reconstituted in 0.1% formic acid (FA) for LC-MS. Recombinant HRI: Immunoprecipitated HRI was incubated with 400 ng purified active ROCK1 in kinase buffer (25 mM Tris, pH 7.5, 10 mM MgCl2, 5 mM β-glycerolphosphate, 0.1 mM Na3VO4, and 2 mM DTT) containing 0.2mM ATP for 60 minutes at 30°C. The kinase reactions were terminated by washing the beads with 25 mM Tris, pH 7.5 and then heating in SDS-PAGE sample buffer. The reactions products were resolved on a 8% SDS-PAGE gel. In-gel digestion was performed for each sample, followed by peptide extraction from the gels. Phosphoenrichment was performed using TiO2 on the peptide extracts for phosphosite identification. Mass spec data were searched against Uniprot mouse database with a parameter setting phosphorylation as dynamic modifications.

Suppl. Fig. 1

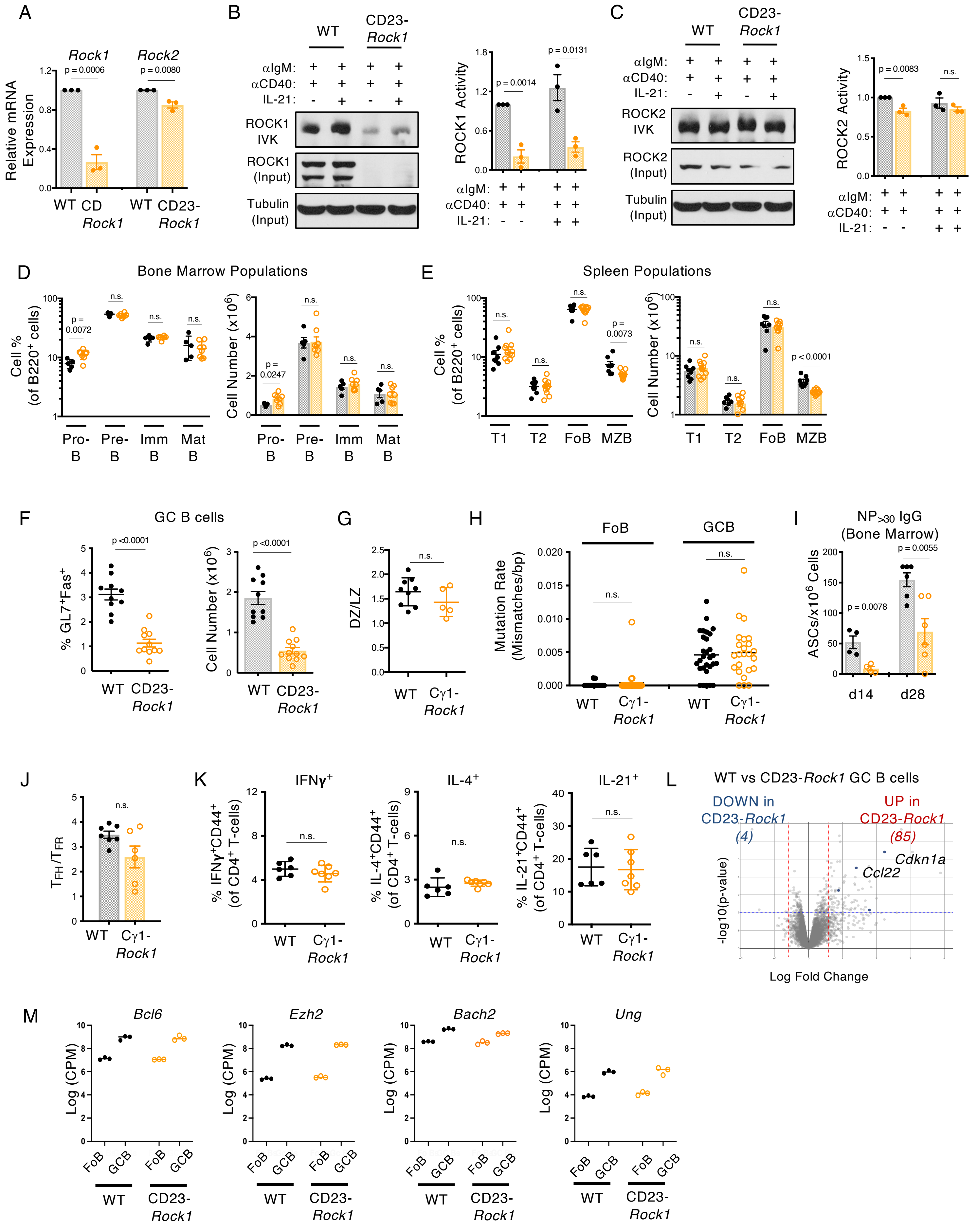

A
Rock1
Rock2
Relative mRNA Expression
p = 0.0006
p = 0.0080
WT CD Rock1
WT CD23-Rock1
B
WT
CD23-Rock1
αIgM:
αCD40:
IL-21:
ROCK1 IVK
ROCK1 (Input)
Tubulin (Input)
ROCK1 Activity
p = 0.0014
p = 0.0131
C
WT
CD23-Rock1
ROCK2 IVK
ROCK2 (Input)
Tubulin (Input)
ROCK2 Activity
p = 0.0083
n.s.
D
Bone Marrow Populations
Cell % (of B220+ cells)
Cell Number (x10^6)
p = 0.0072
p = 0.0247
n.s.
Pro-B
Pre-B
Imm B
Mat B
E
Spleen Populations
p = 0.0073
p < 0.0001
T1
T2
FoB
MZB
F
GC B cells
% GL7+Fas+
Cell Number (x10^6)
p <0.0001
WT
CD23-Rock1
G
DZ/LZ
Cγ1-Rock1
H
FoB
GCB
Mutation Rate (Mismatches/bp)
I
NP>30 IgG (Bone Marrow)
ASCs/x10^6 Cells
p = 0.0078
p = 0.0055
d14
d28
J
TFH/TFR
K
IFNγ+
IL-4+
IL-21+
% IFNγ+CD44+ (of CD4+ T-cells)
% IL-4+CD44+ (of CD4+ T-cells)
% IL-21+CD44+ (of CD4+ T-cells)
L
WT vs CD23-Rock1 GC B cells
DOWN in CD23-Rock1 (4)
UP in CD23-Rock1 (85)
Cdkn1a
Ccl22
-log10(p-value)
Log Fold Change
M
Bcl6
Ezh2
Bach2
Ung
Log (CPM)
FoB
GCB

Supplemental Figure 1. B cell-ROCK1 regulates humoral responses during TD immunization. (A) RT-qPCR data showing the expression of *Rock1* and *Rock2* in purified $CD23^+$ B cells from WT or CD23-*Rock1* mice. Data pooled from 3 independent experiments and show mean +/- SEM; p-value by unpaired two-tailed t-tests. **(B-C)** $CD23^+$ B cells from WT *(black)* and CD23-*Rock1 (orange)* mice were cultured with combinations of αIgM (5μg/mL), αCD40 (5μg/mL), and IL-21 (50ng/mL) for 3d. ROCK1 (B) and ROCK2 (C) *in vitro* kinase activity assays (IVKs) were performed on extracts obtained from the B cell cultures. Quantifications show densitometry ratio of pMYPT1 to ROCK input levels. Data representative of and/or pooled from 3 independent experiments and show mean +/- SEM; p-value by unpaired two-tailed t-tests. **(D-E)** Quantifications of pro-B cells (*pro-B;* $B220^+IgM^-CD43^+$), pre-B cells (*pre-B;* $B220^+IgM^-CD43^-$), immature B cells (*Imm B;* $B220^+IgM^{lo}$), and mature B cells (*Mat B;* $B220^+IgM^{hi}$) from the bone marrow (D) and of transitional T1 B cells (*T1;* $B220^+CD23^-CD21^{lo}IgM^{hi}$), transitional T2 B cells (*T2;* $B220^+CD23^+CD21^+IgM^{hi}$), follicular B cells (*FoB;* $B220^+CD23^+CD21^{mid/lo}IgM^{mid/lo}$), and marginal zone B cells (*MZB;* $B220^+CD23^-CD21^{hi}IgM^{hi}$) from the spleens (E) of the indicated mice. Data pooled from 5 WT and 9 CD23-*Rock1* mice (D) or 8 WT and 11 CD23-*Rock1* mice (E) across 2 (D) or 4 (E) independent experiments and show mean +/- SEM; p-value by unpaired two-tailed t-tests. **(F)** WT *(black)* and CD23-*Rock1 (orange)* mice were immunized ip with 100μg NP-CGG and were assessed for spleen germinal center (GC) B cells (F; $B220^+GL7^+$ Fas^+) by flow cytometry at day 7. Data pooled from 10 WT and 11 CD23-*Rock1* mice across 5 independent experiments and show mean +/- SEM; p-value by unpaired two-tailed t-tests. **(G)** Ratio of dark ($CXCR4^{hi}$ $CD86^{lo}$)/light ($CXCR4^{lo}$ $CD86^{hi}$) (DZ/LZ) zone GC B cells from WT and Cγ1-*Rock1* immunized mice at day 7. Data pooled from 9 WT and 5 Cγ1-*Rock1* mice across 4 independent experiments and show mean +/- SEM; p-value by unpaired two-tailed t-test. **(H)** Plot showing the mutation frequency of the 470-bp JH4 region in sorted FoBs ($B220^+GL7^-CD38^{hi}CD23^+$) as control and GC B cells ($B220^+GL7^+CD38^{lo}$) on day 14 after immunization. *n*>36 clones from 4 mice per genotype. Data pooled from at least 23 clones per cell type per genotype across 2 independent experiments and show mean +/- SEM; p-value by Mann-Whitney test. **(I)** Quantifications of ELISPOTs performed on suspensions from bone marrow from WT and Cγ1-*Rock1* mice at d14 or d28 after immunization as indicated. Data pooled from 4 mice per genotype at d14 and from 6 mice per genotype at d28 and show mean +/- SEM; p-value by unpaired two-tailed t-tests. **(J-K)** Ratio of T-follicular helper cells/T-regulatory cells (T_{fh}/T_{fr}) and frequencies of cytokine producing T-cells (IFN-γ, IL-4 and IL-21) from WT and Cγ1-*Rock1* immunized mice at day 10. Data pooled from 7 WT and 6 Cγ1-*Rock1* mice (J) or from 6 WT and 7 Cγ1-*Rock1* mice (K) across 2 independent experiments and show mean +/- SEM; p-value by unpaired two-tailed t-tests. **(L-M)** WT or CD23-*Rock1* mice were immunized ip with 100 μg NP-CGG and at day 7 GC B cells ($B220^+GL7^+CD38^{lo}$) were sorted for bulk RNA-Seq analysis. Data shown are from 3 independent experiments. (L) Volcano plot shows differentially expressed genes ($Log_2FC>0.58$, unadjusted $p<0.01$) between WT and CD23-*Rock1* GC B cells. (M) Plots show the normalized log-transformed counts per millions for the indicated genes from the RNA-seq analysis.

Suppl. Fig. 2

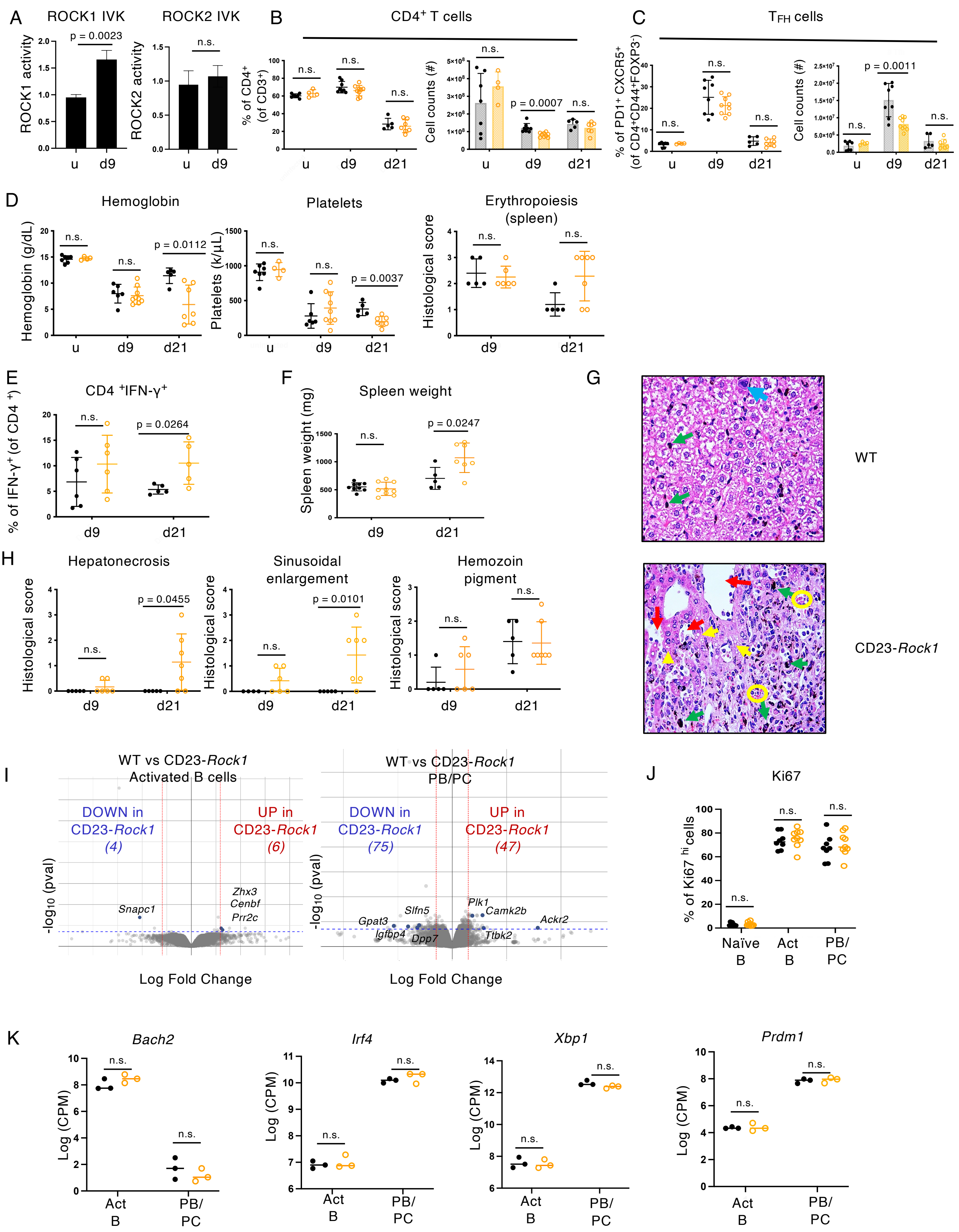

A
ROCK1 IVK
p = 0.0023
ROCK1 activity
u
d9
ROCK2 IVK
n.s.
ROCK2 activity
B
CD4+ T cells
% of CD4+ (of CD3+)
Cell counts (#)
n.s.
p = 0.0007
u
d9
d21
C
TFH cells
% of PD1+ CXCR5+ (of CD4+CD44+FOXP3-)
Cell counts (#)
p = 0.0011
n.s.
D
Hemoglobin
Hemoglobin (g/dL)
p = 0.0112
Platelets
Platelets (k/µL)
p = 0.0037
Erythropoiesis (spleen)
Histological score
E
CD4 +IFN-γ+
% of IFN-γ+ (of CD4 +)
p = 0.0264
F
Spleen weight
Spleen weight (mg)
p = 0.0247
G
WT
CD23-Rock1
H
Hepatonecrosis
p = 0.0455
Sinusoidal enlargement
p = 0.0101
Hemozoin pigment
Histological score
I
WT vs CD23-Rock1
Activated B cells
DOWN in CD23-Rock1 (4)
UP in CD23-Rock1 (6)
Snapc1
Zhx3
Cenbf
Prr2c
-log10 (pval)
Log Fold Change
WT vs CD23-Rock1
PB/PC
DOWN in CD23-Rock1 (75)
UP in CD23-Rock1 (47)
Gpat3
Slfn5
Igfbp4
Dpp7
Plk1
Camk2b
Ttbk2
Ackr2
J
Ki67
% of Ki67 hi cells
Naïve B
Act B
PB/PC
K
Bach2
Irf4
Xbp1
Prdm1
Log (CPM)
Act B
PB/PC

Supplemental Figure 2. ROCK1 controls humoral and pathological responses to *Plasmodium* infection. (A) Quantifications of p-MYPT1 to input ROCK1 (left panel) or ROCK2 (right panel) protein expression for Fig. 2A. Data show mean +/- SEM; p-value by unpaired two-tailed t-tests from 3 independent experiments. **(B-C)** WT (black) or CD23-*Rock1* mice (orange) were infected with 10^6 *Plasmodium yoelii* 17XNL-infected erythrocytes. Quantifications of splenic total $CD4^+$ T-cells ($CD3^+CD4^+$) (B) and T_{FH} cells ($CD3^+CD4^+CD44^+CXCR5^+PD1^+FOXP3^-$) (C) from uninfected or infected mice at day 9 (d9) or day 21 (d21) pi. Data (for all panels) from at least 5 mice per day and per genotype across 3 independent experiments and show mean +/- SEM; p-value by unpaired two-tailed t-tests. **(D)** Plots showing blood hemoglobin levels, platelet counts, and splenic red pulp erythropoiesis (as determined by H&E stain). Data from at least 5 mice per day and per genotype across 3 independent experiments show mean +/- SEM; p-value by unpaired two-tailed t-tests (hemoglobin and platelets) and non-parametric Mann-Whitney test (red pulp erythropoiesis) between the two genotypes for each day of infection. **(E)** Frequencies of $CD4^+$ $IFN\gamma^+$ T-cells. **(F)** Spleen weight of WT and CD23-*Rock1* mice at d9 or d21 pi. **(G)** Representative histological images of liver as determined by H&E stain. Scale bars: 50 μm. *n* = at least 4 mice per genotype from 3 independent experiments. WT: Green arrows show few small deposits of hemozoin. Blue arrows show mildly damaged hepatocytes and Kupfer cells with occasional cell death. CD23-*Rock1* mice: red arrows show sinusoid congestion; green arrows show haemozoin deposition in Kupfer cells; yellow arrows show hepatocyte fat changes and necrosis; circles show parasitized red cells. **(H)** Scores of histological images of liver at d9 or d21 pi. Data from at least 5 mice per day and per genotype across 3 independent experiments show mean +/- SEM; p-value by non-parametric Mann-Whitney test between the two genotypes for each day of infection. **(I)** Splenic B cell populations were sorted from WT or CD23-*Rock1* mice at d9 pi with 10^6 *Plasmodium yoelii* 17XNL-infected erythrocytes and subjected to bulk RNA-Seq analysis. Data shown are from 3 independent experiments. Volcano plot shows the genes differentially expressed ($Log_2FC>0.58$, unadjusted $p<0.01$) between WT and CD23-*Rock1* activated B cells, and PB/PCs. **(J)** Plots showing quantification of the percentage of $Ki67^{hi}$ cells as assessed by flow cytometry in WT (black) and CD23-Rock1 (orange) mice d9 pi. Data from at least 5 mice per day and per genotype across 3 independent experiments show mean +/- SEM; p-value by unpaired two-tailed t-tests. **(K)** Plots showing the normalized log-transformed counts per millions for the indicated genes from the RNA-seq analysis. Data show mean +/- SEM; p-value by unpaired two-tailed t-tests.

Suppl. Fig. 3

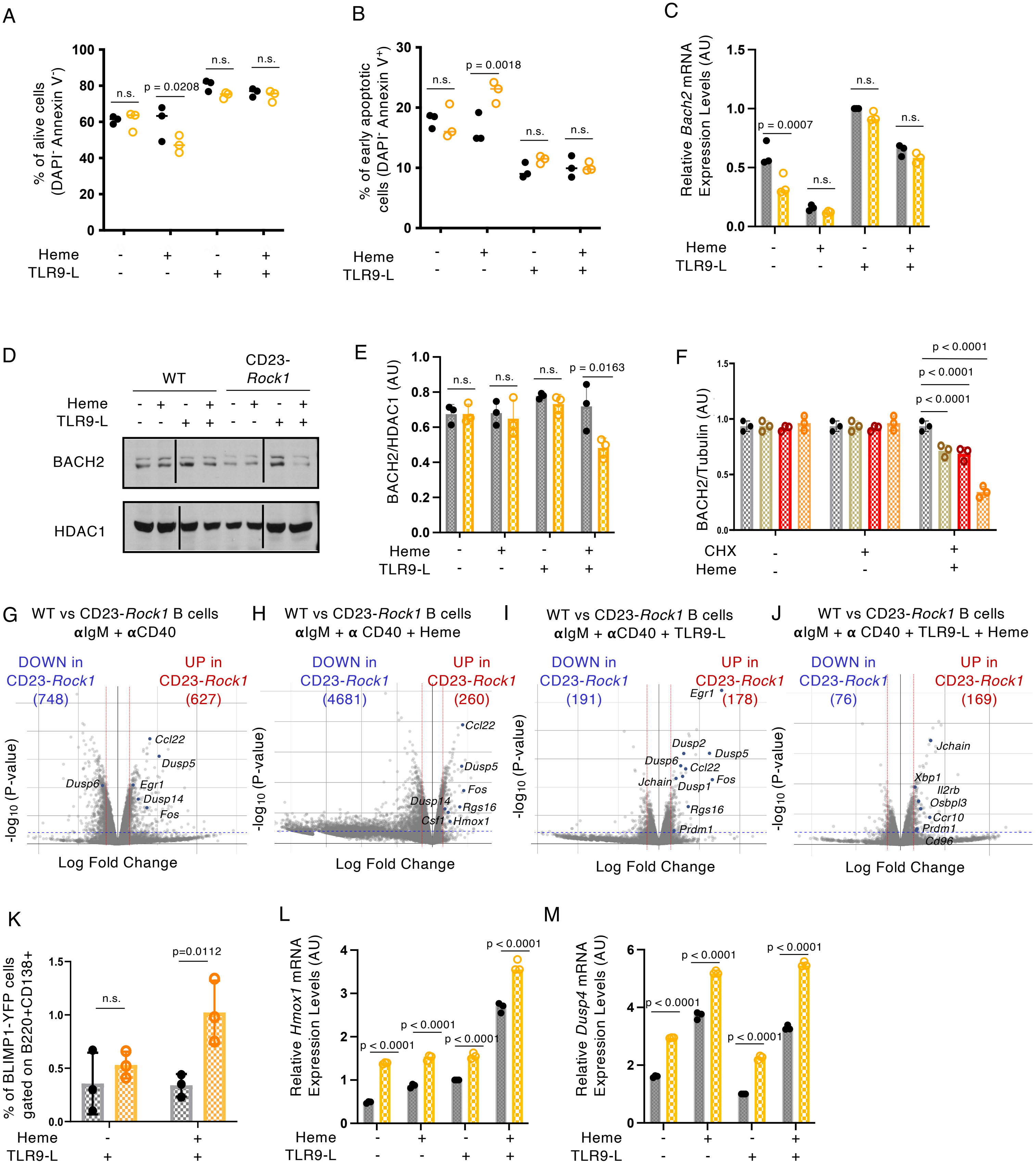
A
% of alive cells (DAPI- Annexin V-)
n.s.
p = 0.0208
n.s.
n.s.
Heme - + - +
TLR9-L - - + +
B
% of early apoptotic cells (DAPI- Annexin V+)
n.s.
p = 0.0018
n.s.
n.s.
Heme - + - +
TLR9-L - - + +
C
Relative Bach2 mRNA Expression Levels (AU)
p = 0.0007
n.s.
n.s.
n.s.
Heme - + - +
TLR9-L - - + +
D
WT
CD23-Rock1
Heme - + - + - + - +
TLR9-L - - + + - - + +
BACH2
HDAC1
E
BACH2/HDAC1 (AU)
n.s.
n.s.
n.s.
p = 0.0163
Heme - + - +
TLR9-L - - + +
F
BACH2/Tubulin (AU)
p < 0.0001
p < 0.0001
p < 0.0001
CHX - + +
Heme - - +
G WT vs CD23-Rock1 B cells αIgM + αCD40
DOWN in CD23-Rock1 (748)
UP in CD23-Rock1 (627)
Ccl22
Dusp5
Dusp6
Egr1
Dusp14
Fos
-log10 (P-value)
Log Fold Change
H WT vs CD23-Rock1 B cells αIgM + α CD40 + Heme
DOWN in CD23-Rock1 (4681)
UP in CD23-Rock1 (260)
Ccl22
Dusp5
Fos
Dusp14
Rgs16
Csf1
Hmox1
-log10 (P-value)
Log Fold Change
I WT vs CD23-Rock1 B cells αIgM + αCD40 + TLR9-L
DOWN in CD23-Rock1 (191)
UP in CD23-Rock1 (178)
Egr1
Dusp2
Dusp5
Dusp6
Ccl22
Jchain
Dusp1
Fos
Rgs16
Prdm1
-log10 (P-value)
Log Fold Change
J WT vs CD23-Rock1 B cells αIgM + α CD40 + TLR9-L + Heme
DOWN in CD23-Rock1 (76)
UP in CD23-Rock1 (169)
Jchain
Xbp1
Il2rb
Osbpl3
Ccr10
Prdm1
Cd96
-log10 (P-value)
Log Fold Change
K
% of BLIMP1-YFP cells gated on B220+CD138+
n.s.
p=0.0112
Heme - +
TLR9-L + +
L
Relative Hmox1 mRNA Expression Levels (AU)
p < 0.0001
p < 0.0001
p < 0.0001
p < 0.0001
Heme - + - +
TLR9-L - - + +
M
Relative Dusp4 mRNA Expression Levels (AU)
p < 0.0001
p < 0.0001
p < 0.0001
p < 0.0001
Heme - + - +
TLR9-L - - + +

Supplemental Figure 3. ROCK1 phosphorylates BACH2 and controls its stability. Purified $CD23^+$ B cells from WT (black) and CD23-*Rock1* (orange) mice were cultured with αIgM (5μg/mL) + αCD40 (5μg/mL), +/- combinations of a TLR9-L (1μg/ml) and heme (60μM) as indicated for 3d.**(A-B)** Flow cytometry analysis of viable (Annexin V^- PI^-) (A) or apoptotic (Annexin V^+ PI^-) cells (B). Data are from 3 independent experiments and show mean +/- SEM; p-value by 2-way ANOVA followed by Sidak's test for multiple comparisons. **(C)** RT-qPCR showing *Bach2* expression under the indicated conditions relative to WT TLR9-L treatment, whose value was set at one. Data are from 3 independent experiments and show mean +/- SEM; p-value by 2-way ANOVA followed by Sidak's test for multiple comparisons. **(D-E)** Representative immunoblot of BACH2 protein levels under the indicated conditions (D). Quantification shows densitometry ratio of BACH2 to HDAC1 levels (E). Data pooled from 3 independent experiments and show mean +/- SEM; p-value by 2-way ANOVA followed by Sidak's test for multiple comparisons. **(F)** Quantification shows densitometry ratio of BACH2 to tubulin levels for Fig. 3F. FLAG-tagged wildtype BACH2 (WT) is shown in dark gray, BACH2A376 in light gray, BACH2A718 in red, and BACH2 A376A718 in orange. Data from 3 independent experiments and show mean +/- SEM; p-value by 2-way ANOVA followed by Sidak's test for multiple comparisons. **(G-J)** Volcano plots show the genes differentially expressed ($Log_2FC>0.58$, unadjusted $p<0.01$) between WT and CD23-*Rock1* in the αIgM+αCD40 (G), + heme (H), + TLR9-L (I), or + TLR9-L + heme (J) conditions. Data are from 3 independent experiments. **(K)** Frequencies of $B220^+CD138^+YFP^+$ cells from B cells purified from WT and CD23-*Rock1-Blimp1YFP* B cells and stimulated for 4 days as indicated. Data are from 3 independent experiments and show mean +/- SEM; p-value by 2-way ANOVA followed by Sidak's test for multiple comparisons. **(L-M)** RT-qPCR showing *Hmox1* (L) and *Dusp4* (M) expression under the indicated conditions relative to WT TLR9-L treatment, whose value was set at one. Data are from 3 independent experiments and show mean +/- SEM; p-value by 2-way ANOVA followed by Sidak's test for multiple comparisons.

Suppl. Fig. 4

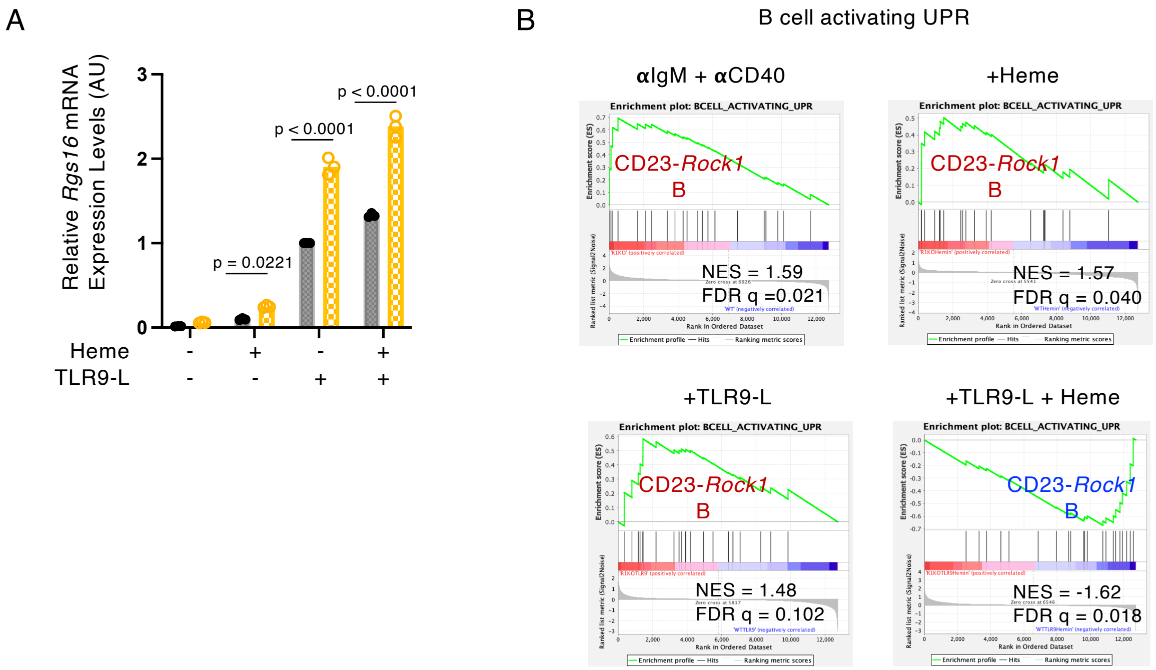

Supplemental Figure 4. In vitro activated ROCK1-deficient B cells exhibit increased proinflammatory and mTORC1-related transcriptional signatures. Purified CD23^{+} B cells from WT and CD23-*Rock1* mice were cultured with αIgM (5μg/mL) + αCD40 (5μg/mL), +/- combinations of a TLR9-L (1μg/ml) and heme (60μM) as indicated for 3d. **(A)** RT-qPCR showing *Rgs16* mRNA expression under the indicated conditions relative to WT TLR9-L treatment, whose value was set at one. Data are from 3 independent experiments and show mean +/- SEM; p-value by 2-way ANOVA followed by Sidak's test for multiple comparisons. **(B)** Gene-set enrichment analysis (GSEA) plots show the enrichment of the B-cell activating UPR gene set (8) in CD23-*Rock1* B cells stimulated as indicated. Significant upregulated enrichment (FDR≤0.1) is depicted in red, downregulated enrichment in blue.

Suppl. Fig. 5

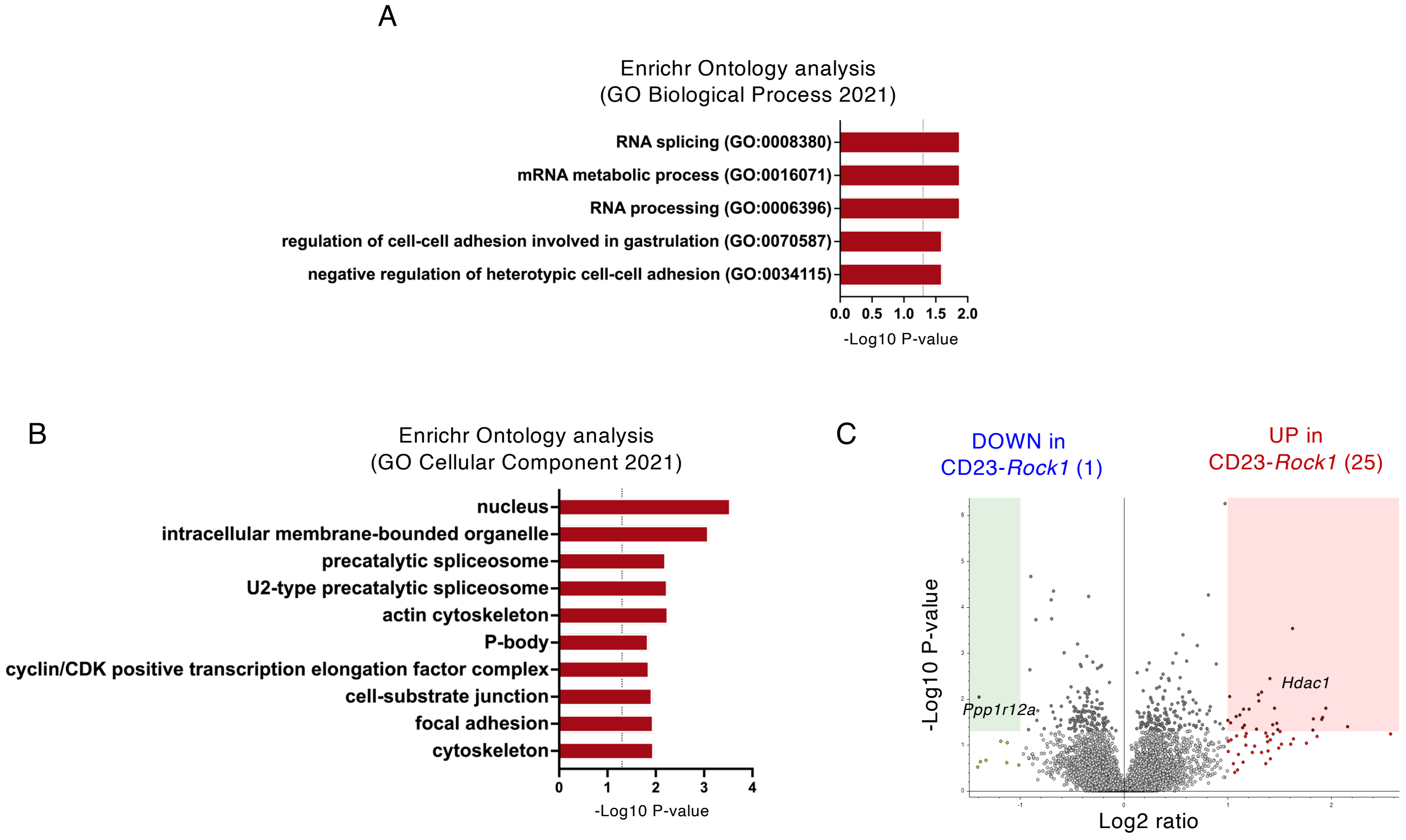

Supplemental Figure 5. B-cell ROCK1 controls a unique phosphoproteomic profile. **(A-B)** Top pathways obtained from the Enrichr Ontology analysis of the differentially enriched phosphoproteins from CD23-*Rock1* versus WT B cells stimulated with αIgM+αCD40 using either GO Biological Process 2021 (A) or GO Cellular Component (B) databases ($p<0.05$). **(C)** Volcano plot shows differentially enriched phosphoproteins in WT (green square) and CD23-*Rock1* (red square) B cells stimulated with αIgM+αCD40+TLR9-L (1μg/ml) for 3d.

Suppl. Figure 6

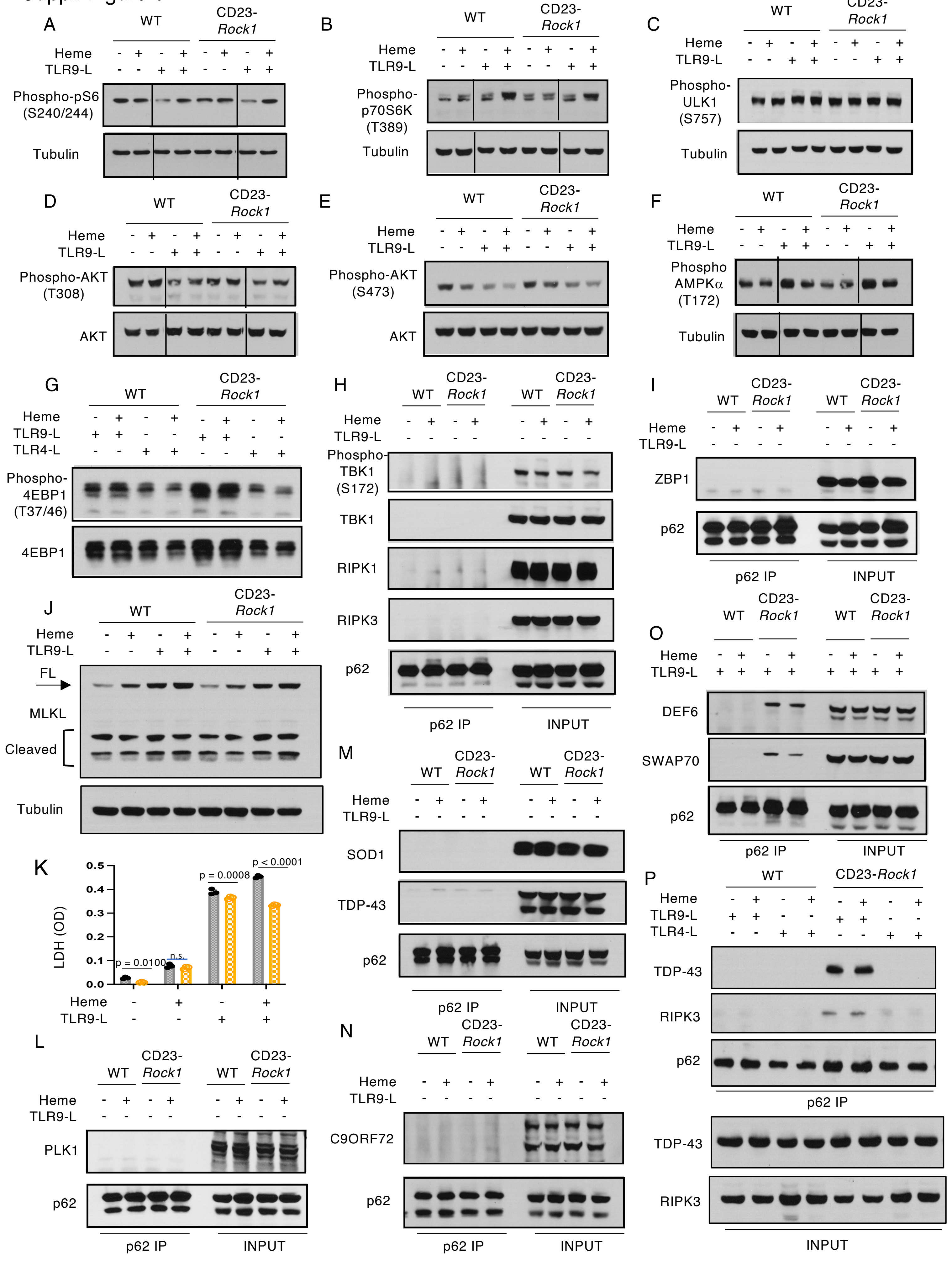

Supplemental Fig. 6. ROCK1 limits the assembly of p62 complexes enriched in mTORC1, ripoptosome components, and ALS-linked molecules. Purified CD23$^+$ B cells from WT and CD23-*Rock1* mice were cultured with αIgM (5μg/mL) + αCD40 (5μg/mL), +/- combinations of a TLR9-L (1μg/ml) and heme (60μM) as indicated for 3d. **(A-F)** Western blotting analysis of the levels of phospho-S6 (A), phospho-70S6K (B), phospho-ULK1 (C), phospho-AKT-T308 (D), phospho-AKT-S473 (E), phospho-AMPKα-T172 (F) in cytoplasmic extracts from WT and CD23-*Rock1* B cells stimulated as indicated. Results are representative of 3 independent experiments. **(G)** Western blotting analysis of the levels of phospho-4EBP1 in cytoplasmic extracts from WT and CD23-*Rock1* B cells stimulated with combinations of a TLR9-L (CpG, 1μg/ml) or a TLR4-L (LPS, 25μg/mL) +/- heme (60μM) as indicated for 3d. Results are representative of 3 independent experiments. **(H-I)** p62 was immunoprecipitated from cytoplasmic extracts of WT or CD23-*Rock1* B cells stimulated as indicated. The immunoprecipitates were probed by Western blotting for the presence of phospho-TBK1, TBK1, RIPK1, and RIPK3 (H); ZBP1 (I) Results are representative of 3 independent experiments. **(J)** Western blotting analysis of FL and cleaved MLKL in cytoplasmic extracts from WT and CD23-*Rock1* B cells stimulated as indicated. Results are representative of 3 independent experiments. **(K)** LDH levels in the supernatants of WT and CD23-*Rock1* B cells stimulated as indicated as assessed by ELISA. Data pooled from 3 independent experiments and show mean +/- SEM; p-value by 2-way ANOVA followed by Sidak's test for multiple comparisons. **(L-P)** p62 was immunoprecipitated from cytoplasmic extracts of WT or CD23-*Rock1* B cells stimulated as indicated. The precipitates were probed by Western blotting for the presence of PLK1 (L); SOD1 (M), TDP-43 (M, P), C9ORF72 (N), DEF6 (O), SWAP-70 (O), and RIPK3 (P). Results are representative of 3 independent experiments.

Suppl. Fig. 7

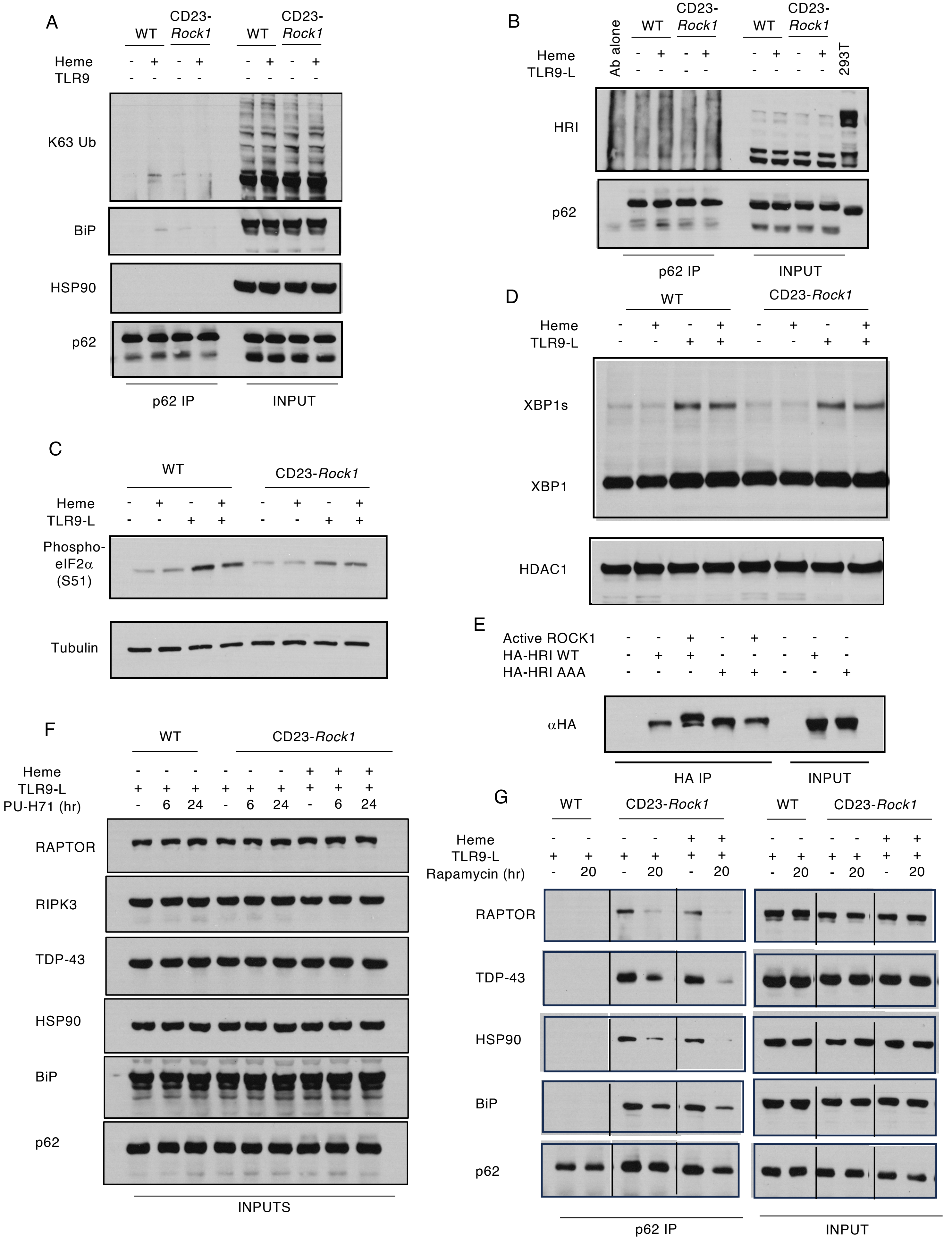
A
WT
CD23-*Rock1*
Heme
TLR9
K63 Ub
BiP
HSP90
p62
p62 IP
INPUT
B
Ab alone
293T
Heme
TLR9-L
HRI
p62
p62 IP
INPUT
C
Phospho-eIF2α (S51)
Tubulin
D
XBP1s
XBP1
HDAC1
E
Active ROCK1
HA-HRI WT
HA-HRI AAA
αHA
HA IP
INPUT
F
PU-H71 (hr)
RAPTOR
RIPK3
TDP-43
HSP90
BiP
p62
INPUTS
G
Rapamycin (hr)
RAPTOR
TDP-43
HSP90
BiP
p62
p62 IP
INPUT

Supplemental Fig. 7. ROCK1 regulates HRI. Purified CD23$^+$ B cells from WT and CD23-*Rock1* mice were cultured with αIgM (5μg/mL) + αCD40 (5μg/mL), +/- combinations of a TLR9-L (1μg/ml) and heme (60μM) as indicated for 3d. **(A-B)** p62 was immunoprecipitated from cytoplasmic extracts of WT or CD23-*Rock1* B cells stimulated as indicated. The precipitates were probed by Western blotting for the presence of K63-ubiquitinated proteins, BiP, HSP90 (A), and HRI (B). Results are representative of 3 independent experiments. **(C)** Western blotting analysis of the levels of phospho-eIF2α in extracts from WT and CD23-*Rock1* B cells stimulated as indicated. Results are representative of 3 independent experiments. **(D)** Western blotting analysis of the levels of total XBP1 and spliced XBP1 (XBP1s) in nuclear extracts from WT and CD23-*Rock1* B cells stimulated as indicated. Results are representative of 3 independent experiments. **(E)** 293T cells were transfected with constructs expressing either HA-tagged wildtype HRI (WT) or an HA-tagged triple mutant of HRI (HRI-AAA where S5, S144, and S293 are mutated to alanine). HA-tagged HRI WT or HA-tagged HRIAAA was then immunoprecipitated and incubated in the presence/absence of CA-ROCK1 followed by immunoblotting with an anti-HA antibody. Results are representative of 3 independent experiments. **(F)** Purified CD23$^+$ B cells from WT and CD23-*Rock1* mice were cultured as indicated for 3d. DMSO or PU-H71 (1μM) were added for the last 6 or 24 hrs of culture as indicated. Protein levels of raptor, RIPK3, TDP-43, HSP90, BiP, and p62 were assessed by Western blotting. Results are representative of 3 independent experiments. **(G)** Purified CD23$^+$ B cells from WT and CD23-*Rock1* mice were cultured as indicated for 3d. DMSO or Rapamycin (20 nM) were added for the last 20 hrs of culture as indicated. Protein levels of raptor, TDP-43, HSP90, BiP, and p62 were assessed by Western blotting. Results are representative of 3 independent experiments.

Suppl. Fig. 8

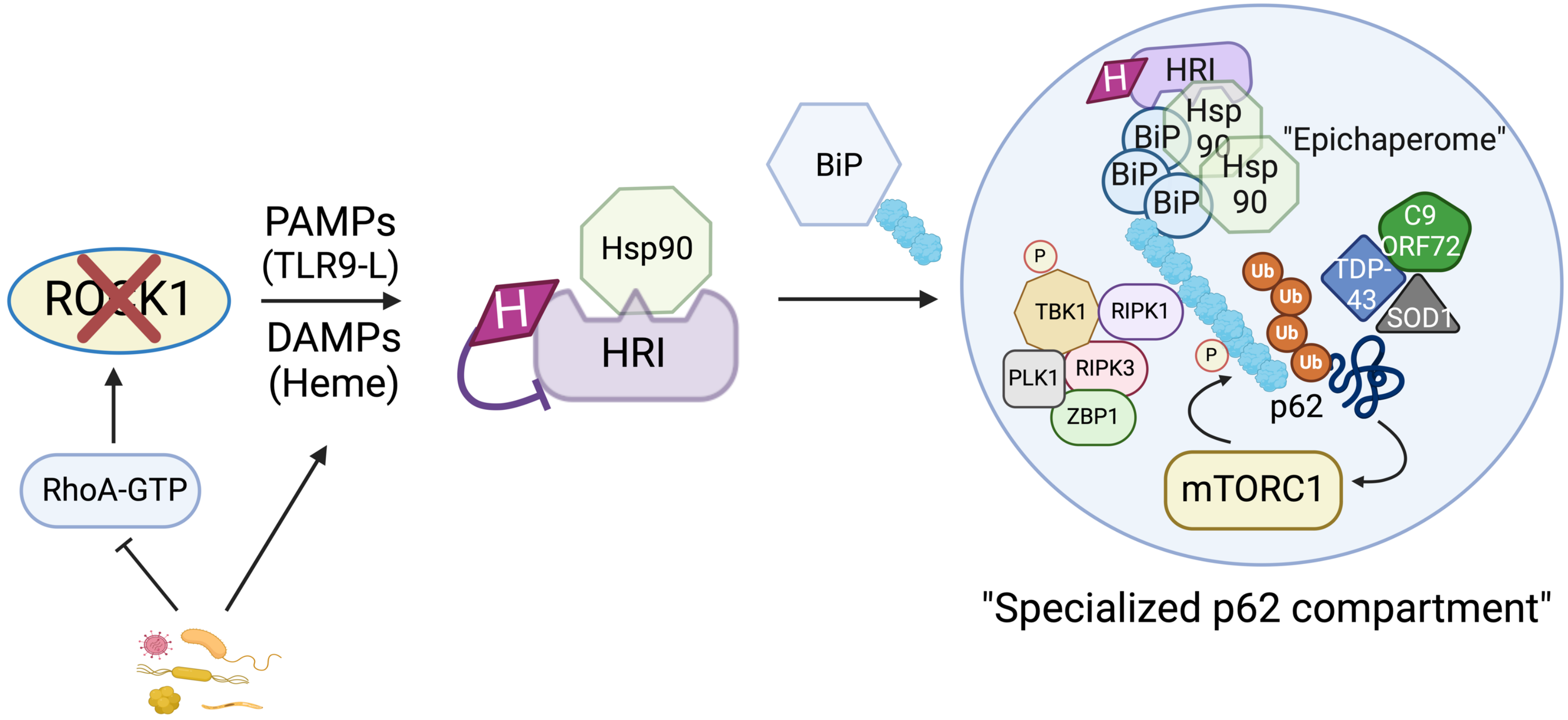

Supplemental Fig. 8. Model summarizing the putative mechanism by which ROCK1 regulates p62 complex assembly. Hypothetical model summarizing the mechanism by which the absence of ROCK1 can lead to the assembly of unique p62 compartments. In the absence of ROCK1, HRI and HSP90 assemble in a complex that, in the presence of stressors like PAMPs and DAMPs, facilitates the interaction of HSP90 with p62-associated BiP, leading to the subsequent formation of epichaperome-like complexes, and the stabilization of a distinctive subset of p62 aggregates that contain key kinases like mTORC1 and critical regulators of RNA metabolism, translation, proteostasis, and inflammation. Assembly of such compartments could represent an “emergency response” to enable more efficient signaling, tightly regulate the activity of major kinases, couple the high demands of antibody secretion with available resources, and engage additional effector capabilities. Created in BioRender. Pernis, A. (2025) https://BioRender.com/l44d648.

Supplementary Table 1. Key residues whose phosphorylation was significantly downregulated ($Log_2FC>1$, $p<0.05$) in CD23-*Rock1* as compared to WT B cells stimulated with αIgM+αCD40.

Symbol	Site	Fold change	p-value
Alpl	S110	-1.38	0.006277
Akap13	S1503, S856	-1.04	0.029862
Ppp1r12a	S861, S870	-1.02	5.34E-05
Ppp1r12c	S411	-1.01	0.000971

Supplementary Table 2. Key residues whose phosphorylation was significantly upregulated ($Log_2FC>1$, $p<0.05$) in CD23-*Rock1* as compared to WT B cells stimulated with αIgM+αCD40.

Symbol	Site(s)	Fold change	p-value
Ubap2l	S467, S477	1.01	0.011351
Trp53bp1	S298 and S303, S1115	1.01,2.11	8.28E-06, 0.016504
Rassf4	S169, S170	1.02	0.006518
Med19	S226	1.03	0.038293
Baz1b	S345	1.08	0.003771
Son	S319, T320	1.11	0.042263
Usp9x	S590	1.12	0.002624
Sqstm1	S334	1.16	0.015204
Selplg	?	1.18	0.040502
Aimp2	T82	1.19	0.00858
Ubxn7	S395	1.2	0.010171
Numa1	T633	1.23	0.002771
Rplp2	S105	1.23	0.013314
Trim28	Y458	1.24	0.026169
Wnk1	?	1.24	0.003187
Npm1	S70	1.24	0.022411
Scaf11	S752, S755	1.24	0.023313
Cdk12	?	1.24	0.03181
Eif4ebp1	?	1.24	0.03453
Edc4	S773	1.25	0.043652
Hexim1	S103	1.26	0.015491
Map4	S475	1.44	0.036135
Marcksl1	S132, S135	1.47	0.046669
Hdac1	S393	1.51	0.025136
Borcs6	S130	1.52	0.022699
Bcl7a	S157	1.52	0.00537
Acin1	S400	1.55	0.003694
Crtc2	S461, T463	1.57	0.005177
Tfeb	T330	1.77	0.010759
Zyx	S144	1.77	0.004352
Mbp	S112	1.83	0.009694

Fubp1	S629	1.89	0.00997
Srrm2	S1315	1.94	0.009465
Son	S256	2.05	0.012638
Tnks1bp1	S1063	2.18	0.002799
Cast	S290	2.45	0.007393

Supplementary Table 3. Key residues whose phosphorylation was significantly upregulated ($Log_2FC>1$, $p<0.05$) in CD23-*Rock1* as compared to WT B cells stimulated with αIgM+αCD40 only in the presence of a TLR9 ligand.

Symbol	Site	Fold change	p-value
Xrn1	S1668	1	0.02881467
Srsf11	S499	1.02	0.00880385
Cbl	T613	1.08	0.02368171
Sp100	S190	1.28	0.04449481
Sf3b2	S761	1.48	0.04601716
Cad	T1770	1.91	0.02757363

Supplementary Table 4. Selected proteins whose phosphorylation was upregulated in stimulated CD23-*Rock1* compared to WT B cells at sites that are also potential targets of mTOR, RIPK3, TBK1, and PLK1 based on annotated references from PhosphositePlus.

Associated kinase	Phosphoprotein
mTORC1, RIPK3, TBK1, PLK1	Hdac1, Npm1
mTORC1, RIPK3, TBK1	Sqstm1, Trp53bp1
mTORC1, RIPK3, PLK1	Map4
mTORC1, RIPK3	Akap13, Hexim1, Ubxn7
mTORC1, TBK1	Med19
mTORC1, PLK1	Marcksl1
RIPK3	Crtc2, Son

Supplementary Table 5. Murine RT-qPCR Primers

Target	Primer	Sequence (5' -> 3')	Source
Dusp4	Forward	5’-CGTGCGCTGCAATACCATC-3’	PrimerBank; www.pga.mgh.harvard.edu/primerbank
	Reverse	5’-CTCATAGCCACCTTTAAGCAGG-3’	PrimerBank; www.pga.mgh.harvard.edu/primerbank
Egr1	Forward	5’-ACGACAGCAGTCCCATCTACTCGG-3’	PMID: **29440259**
	Reverse	5’-GGACTCGACAGGGCAAGCATATGG-3’	PMID: **29440259**
Fos	Forward	5’-CGGGTTTCAACGCCGACTA-3’	PrimerBank; www.pga.mgh.harvard.edu/primerbank
	Reverse	5’-TTGGCACTAGAGACGGACAGA-3’	PrimerBank; www.pga.mgh.harvard.edu/primerbank
Hmox1	Forward	5’-AAGCCGAGAATGCTGAGTTCA-3’	PrimerBank; www.pga.mgh.harvard.edu/primerbank
	Reverse	5’-GCCGTGTAGATATGGTACAAGGA-3’	PrimerBank; www.pga.mgh.harvard.edu/primerbank
Rgs16	Forward	5’-CCATGCCTTCCTAAAGACGGA-3’	PrimerBank; www.pga.mgh.harvard.edu/primerbank
	Reverse	5’-GTACTCGTCAAAGATGTGGTGAG-3’	PrimerBank; www.pga.mgh.harvard.edu/primerbank

Supplementary Table 6. Antibodies for Western blotting and Immunoprecipitation

Target	Antibody Type	Assay	Dilution	Source, Catalog #
AKT	Rabbit, Polyclonal	Western blotting	1 : 1000	Cell Signaling, #9272
ATF4	Rabbit, Monoclonal	Western blotting	1 : 1000	Cell Signaling, #11815
BACH2	Rabbit, Polyclonal	Western blotting	1 : 1000	Rockland, # 600-401-H35
BiP	Rabbit, Polyclonal	Western blotting	1 : 1000	Proteintech, #11587-1-AP
b-Tubulin	Mouse, Monoclonal	Western blotting	1 : 2000	Sigma, #T0198
C9ORF72	Rabbit, Polyclonal	Western blotting	1 : 1000	Proteintech, #22637-1-AP
DEF6	Rabbit, Polyclonal	Western blotting	1 : 1000	PMID: **12651066**
FLAG	Mouse, Monoclonal(M2), HRP-conjugated	Western blotting	1 : 2000	Sigma, A8952
FLAG	Mouse, Monoclonal(M2), Agarose-conjugated	Immunoprecipitation	1 : 10	Sigma, A2220
4E-BP1	Rabbit, Monoclonal	Western blotting	1 : 1000	Cell Signaling, #9644

HA	Rat, Monoclonal(3F10), HRP-conjugated	Western blotting	1 : 2000	Roche, 12013819001
HA	Rat, Monoclonal(3F10), Agarose-conjugated	Immunoprecipitation	1 : 10	Roche, 11815016001
HDAC1	Rabbit, Polyclonal	Western blotting	1 : 1000	Cell Signaling, #2062
HRI	Rabbit, Polyclonal	Western blotting	1 : 1000	Proteintech, #20499-1-AP
HSP90	Mouse, Monoclonal	Western blotting	1 : 2000	Santa Cruz, sc-13119 (F-8)
HSP90	Rabbit, Monoclonal	Western blotting	1 : 2000	Cell Signaling, #4877
IRF4	Rabbit, Monoclonal	Western blotting	1 : 3000	Cell Signaling, #15106
Keap1	Rabbit, Monoclonal	Western blotting	1 : 1000	Cell Signaling, #8047
K63-Linked PolyUb	Rabbit, Monoclonal	Western blotting	1 : 1000	Cell Signaling, #5621
LC3A/B	Rabbit, Monoclonal	Western blotting	1 : 1000	Cell Signaling, #12741
MLKL	Rabbit, Polyclonal	Western blotting	1 : 1000	Abcepta, #AP14272B

PLK1	Mouse, Monoclonal	Western blotting	1 : 1000	Santa Cruz, sc-17783
p62	Rabbit, Monoclonal	Immunoprecipitation	1 : 200	Cell Signaling, #39749
p62	Rabbit, Polyclonal	Western blotting	1 : 1000	Cell Signaling, #5114
Phospho-p62(S349)	Rabbit, Monoclonal	Western blotting	1 : 1000	Cell Signaling, #16177
Phospho-AKT(T308)	Rabbit, Monoclonal	Western blotting	1 : 1000	Cell Signaling, #13038
Phospho-AKT(S473)	Rabbit, Polyclonal	Western blotting	1 : 1000	Cell Signaling, #9271
Phospho-AMPKa(T172)	Rabbit, Monoclonal	Western blotting	1 : 1000	Cell Signaling, #2535
Phospho-eIF2a(S51)	Rabbit, Polyclonal	Western blotting	1 : 2000	Cell Signaling, #9721
Phospho-4EBP1(T37/46)	Rabbit, Monoclonal	Western blotting	1 : 2000	Cell Signaling, #2855
Phospho-p70S6K(T389)	Rabbit, Monoclonal	Western blotting	1 : 1000	Cell Signaling, #9234
Phospho-S6(S240/244)	Rabbit, Monoclonal	Western blotting	1 : 3000	Cell Signaling, #5364

Phospho-(Ser/Thr) PKA Substrate	Rabbit, Polyclonal	Western blotting	1 : 1000	Cell Signaling, #9621
Phospho-TBK1(S172)	Rabbit, Monoclonal	Western blotting	1 : 1000	Cell Signaling, #5483
Phospho-ULK1(S757)	Rabbit, Monoclonal	Western blotting	1 : 1000	Cell Signaling, #14202
Raptor	Rabbit, Monoclonal	Western blotting	1 : 1000	Cell Signaling, #2280
RIPK1	Rabbit, Monoclonal	Western blotting	1 : 1000	Cell Signaling, #3493
RIPK3	Rabbit, Monoclonal	Western blotting	1 : 1000	Cell Signaling, #15828
ROCK1	Rabbit, Monoclonal	Western blotting<br>Immunoprecipitation	1 : 1000<br>1 : 100	Cell Signaling, #4035
ROCK2	Rabbit, Monoclonal	Western blotting<br>Immunoprecipitation	1 : 1000<br>1 : 100	Cell Signaling, #9029
SOD1	Rabbit, Monoclonal	Western blotting	1 : 1000	Cell Signaling, #37385
SWAP-70	Mouse, Monoclonal	Western blotting	1 : 2000	Santa Cruz, sc-81991 (Q-28)
TBK1	Rabbit, Monoclonal	Western blotting	1 : 1000	Cell Signaling, #38066
TDP43	Rabbit, Monoclonal	Western blotting	1 : 1000	Cell Signaling, #89789

TRAF6	Rabbit, Monoclonal	Western blotting	1 : 1000	Cell Signaling, #67591
XBP1	Mouse, Monoclonal	Western blotting	1 : 1000	BioLegend, #647501<br><br>(143F)
ZBP1	Mouse, Monoclonal	Western blotting	1 : 1000	AdipoGen,<br><br>#AG-20B-0010-C100

REFERENCES

1. Ricker E, Chinenov Y, Pannellini T, Flores-Castro D, Ye C, Gupta S, et al. Serine-threonine kinase ROCK2 regulates germinal center B cell positioning and cholesterol biosynthesis. *J Clin Invest.* 2020;130(7):3654-70.
2. Rivera-Correa J, Guthmiller JJ, Vijay R, Fernandez-Arias C, Pardo-Ruge MA, Gonzalez S, et al. Plasmodium DNA-mediated TLR9 activation of T-bet(+) B cells contributes to autoimmune anaemia during malaria. *Nat Commun.* 2017;8(1):1282.
3. Klinkhamhom A, Glaharn S, Srisook C, Ampawong S, Krudsood S, Ward SA, et al. M1 macrophage features in severe Plasmodium falciparum malaria patients with pulmonary oedema. *Malar J.* 2020;19(1):182.
4. Viriyavejakul P, Khachonsaksumet V, and Punsawad C. Liver changes in severe Plasmodium falciparum malaria: histopathology, apoptosis and nuclear factor kappa B expression. *Malar J.* 2014;13:106.
5. Manni M, Gupta S, Ricker E, Chinenov Y, Park SH, Shi M, et al. Regulation of age-associated B cells by IRF5 in systemic autoimmunity. *Nat Immunol.* 2018;19(4):407-19.
6. Navarrete-Perea J, Yu Q, Gygi SP, and Paulo JA. Streamlined Tandem Mass Tag (SL-TMT) Protocol: An Efficient Strategy for Quantitative (Phospho)proteome Profiling Using Tandem Mass Tag-Synchronous Precursor Selection-MS3. *J Proteome Res.* 2018;17(6):2226-36.
7. Rappsilber J, Mann M, and Ishihama Y. Protocol for micro-purification, enrichment, pre-fractionation and storage of peptides for proteomics using StageTips. *Nat Protoc.* 2007;2(8):1896-906.
8. Gaudette BT, Jones DD, Bortnick A, Argon Y, and Allman D. mTORC1 coordinates an immediate unfolded protein response-related transcriptome in activated B cells preceding antibody secretion. *Nat Commun.* 2020;11(1):723.

Densitometric analysis of immunoblots shown in the indicated Figures. Values are mean +/- SEM of at least three independent experiments. p-values were assessed by 2-way ANOVA followed by Sidak's test for multiple comparisons. WT B cells are shown in gray and CD23-Rock1 B cells in orange.

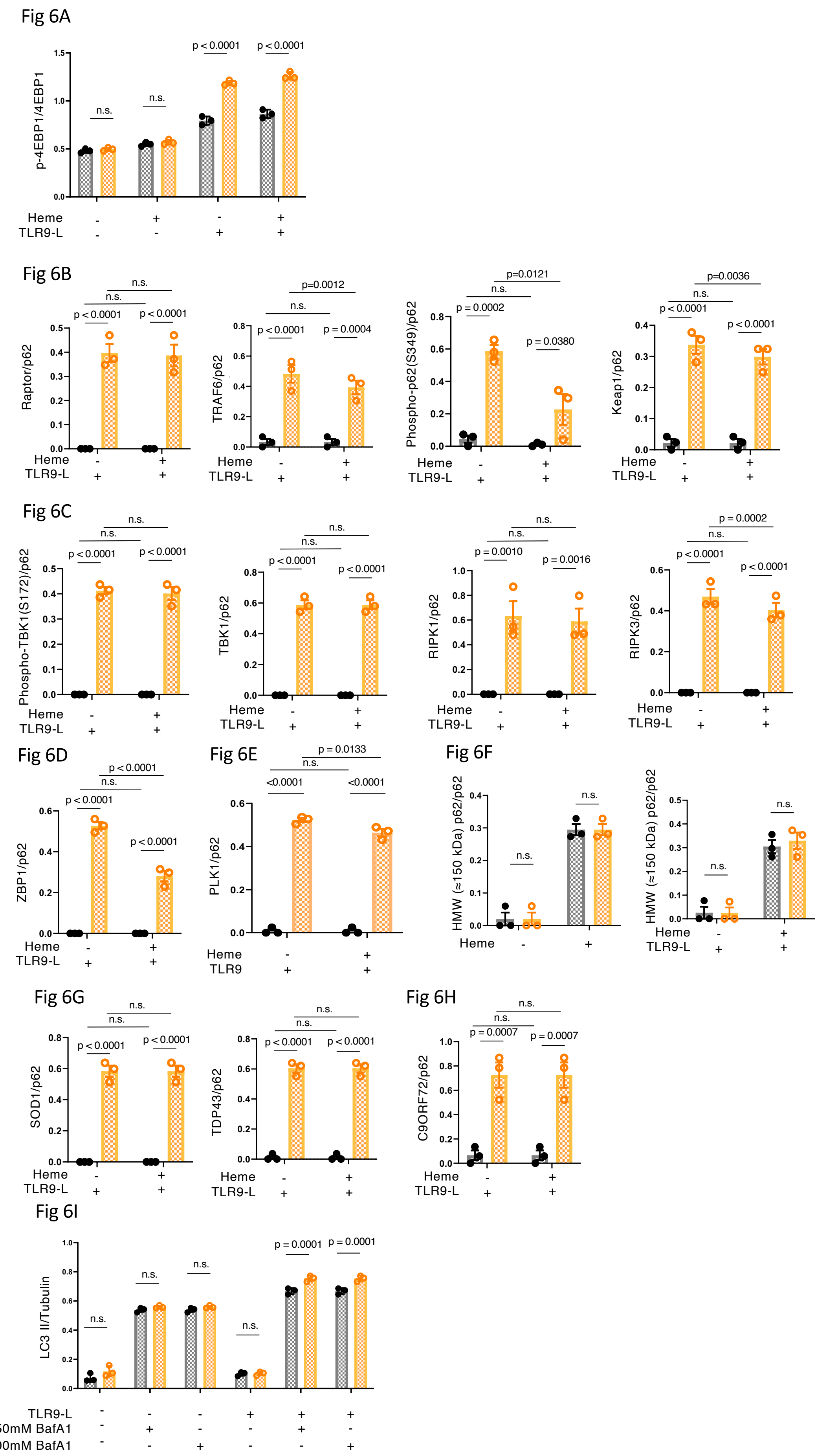

Densitometric analysis of immunoblots shown in the indicated Figures. Values are mean +/- SEM of at least three independent experiments. p-values were assessed by 2-way ANOVA followed by Sidak's test for multiple comparisons. WT B cells are shown in gray and CD23-Rock1 B cells in orange.

Suppl. Fig 6A

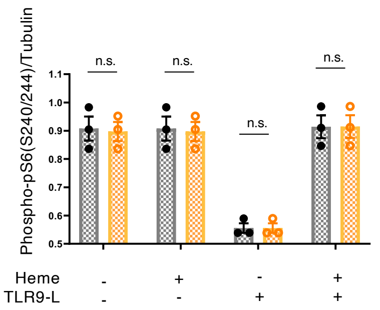

Suppl. Fig 6B

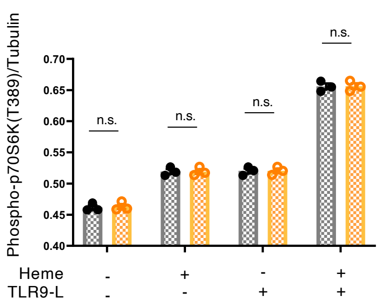

Suppl. Fig 6C

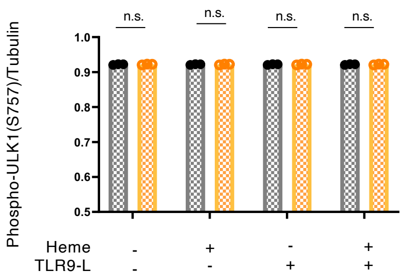

Suppl. Fig 6D

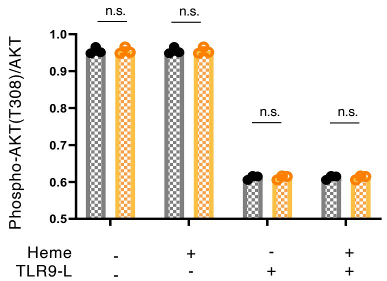

Suppl. Fig 6E

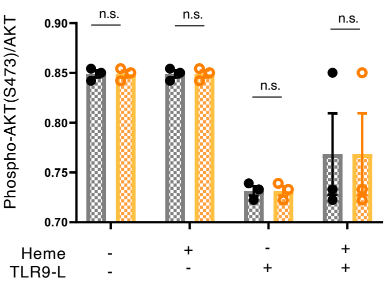

Suppl. Fig 6F

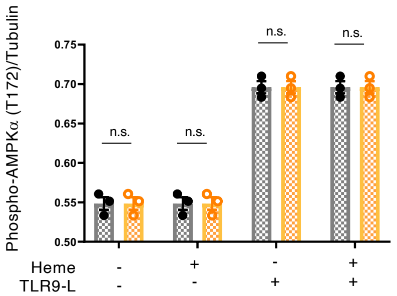

Suppl. Fig. 6G

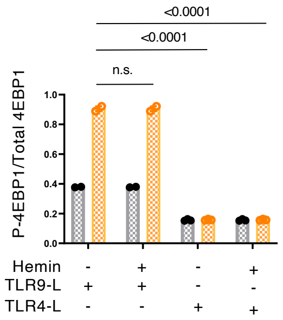

Suppl. Fig 6J

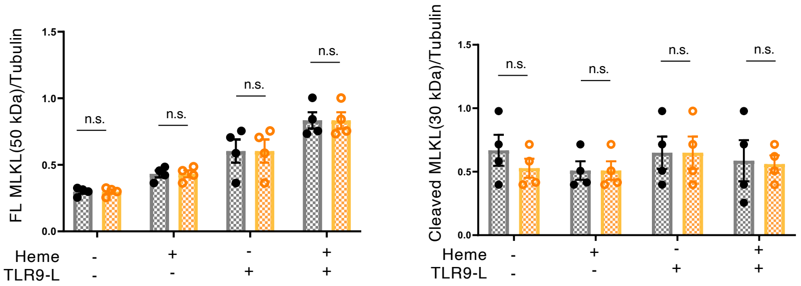

Suppl. Fig 6O

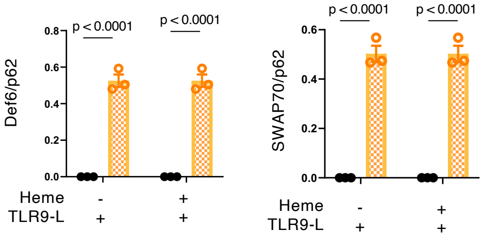

Densitometric analysis of immunoblots shown in the indicated Figures. Values are mean +/- SEM of at least three independent experiments. p-values were assessed by 2-way ANOVA followed by Sidak's test for multiple comparisons except for Fig. 7H where p-values were assessed by one-way ANOVA followed by Sidak's test for multiple comparisons. WT B cells are shown in gray and CD23-Rock1 B cells in orange.

Fig 7A

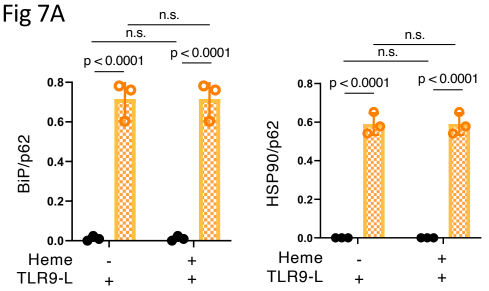

Fig 7B

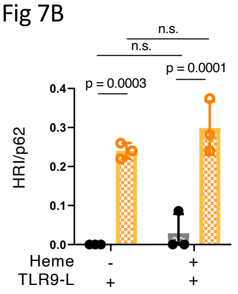

Fig. 7C

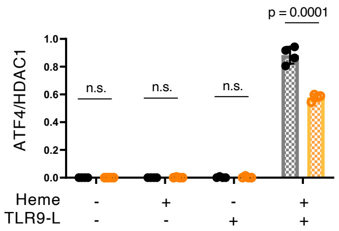

Fig 7H

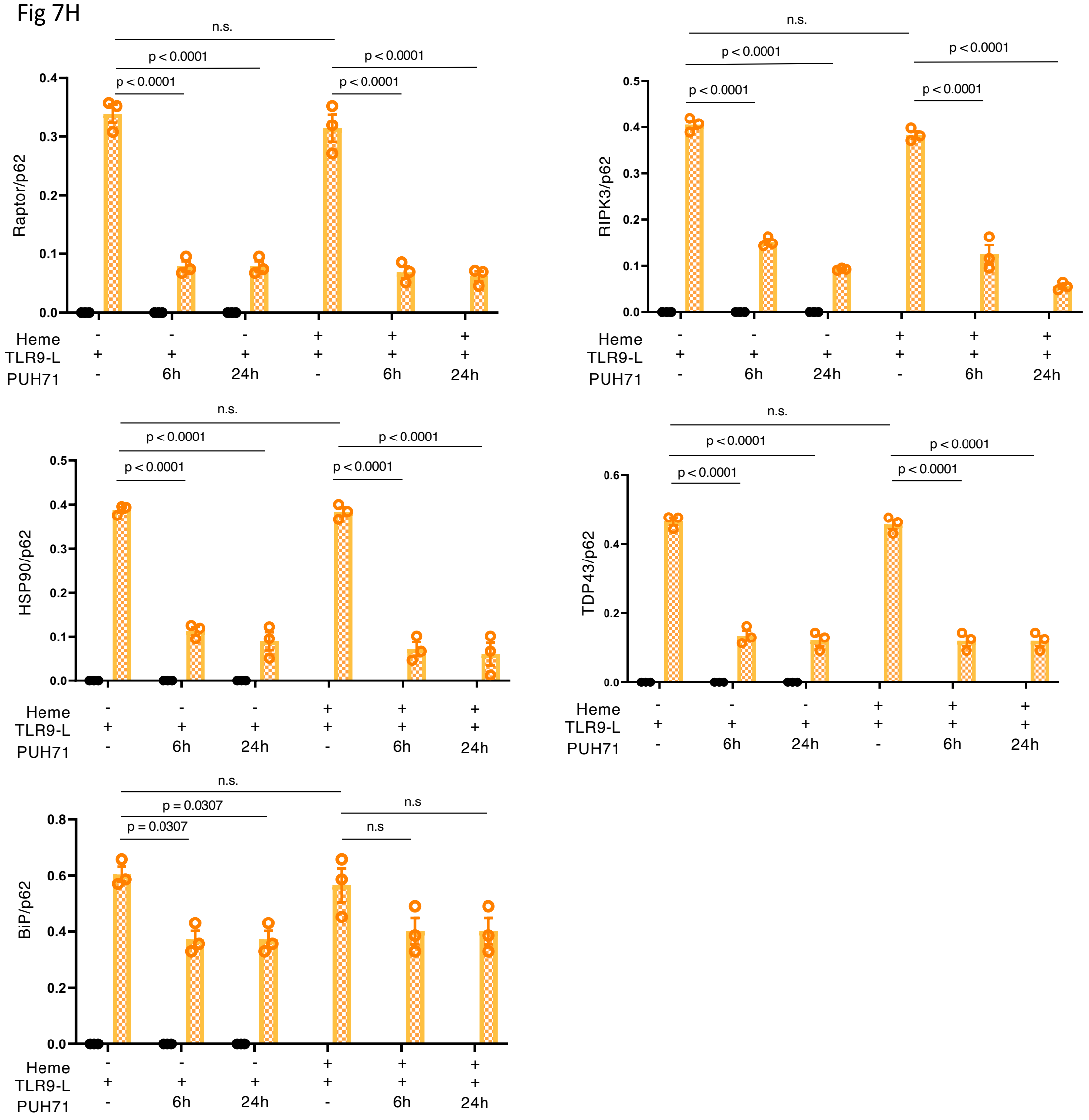

Fig 7I

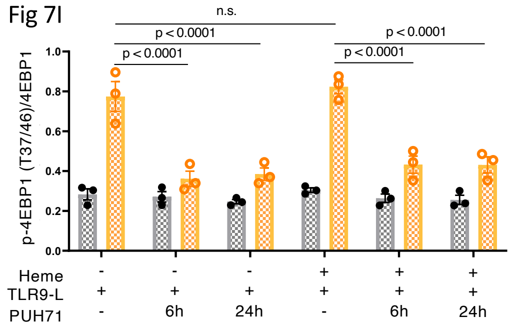

Densitometric analysis of immunoblots shown in the indicated Figures. Values are mean +/- SEM of at least three independent experiments. p-values were assessed by 2-way ANOVA followed by Sidak's test for multiple comparisons except for Fig. 7H where p-values were assessed by one-way ANOVA followed by Sidak's test for multiple comparisons. WT B cells are shown in gray and CD23-Rock1 B cells in orange.

Suppl. Fig 7C

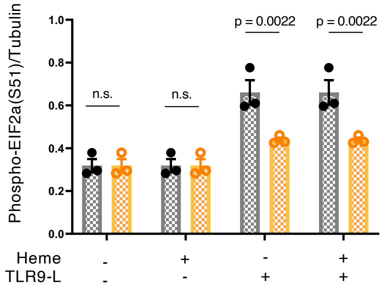

Suppl. Fig 7D

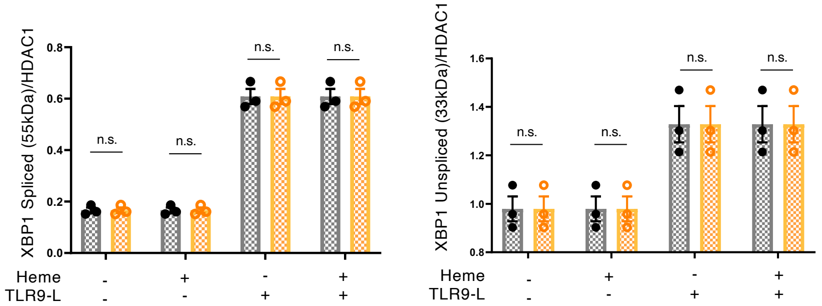

Suppl. Fig 7G

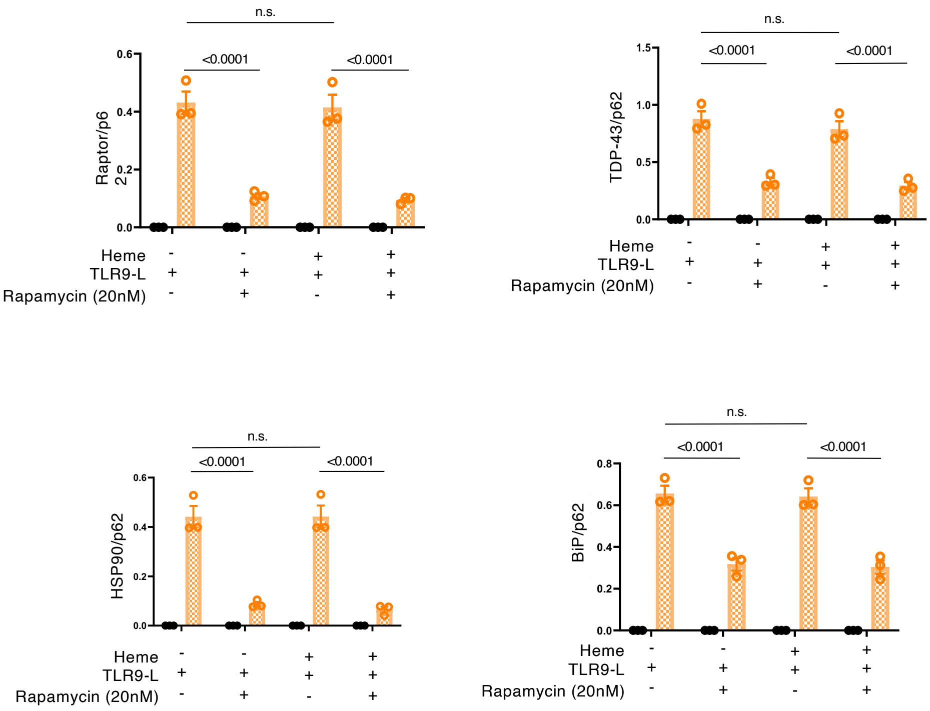

Representative plots and/or gating strategies for the indicated Figures

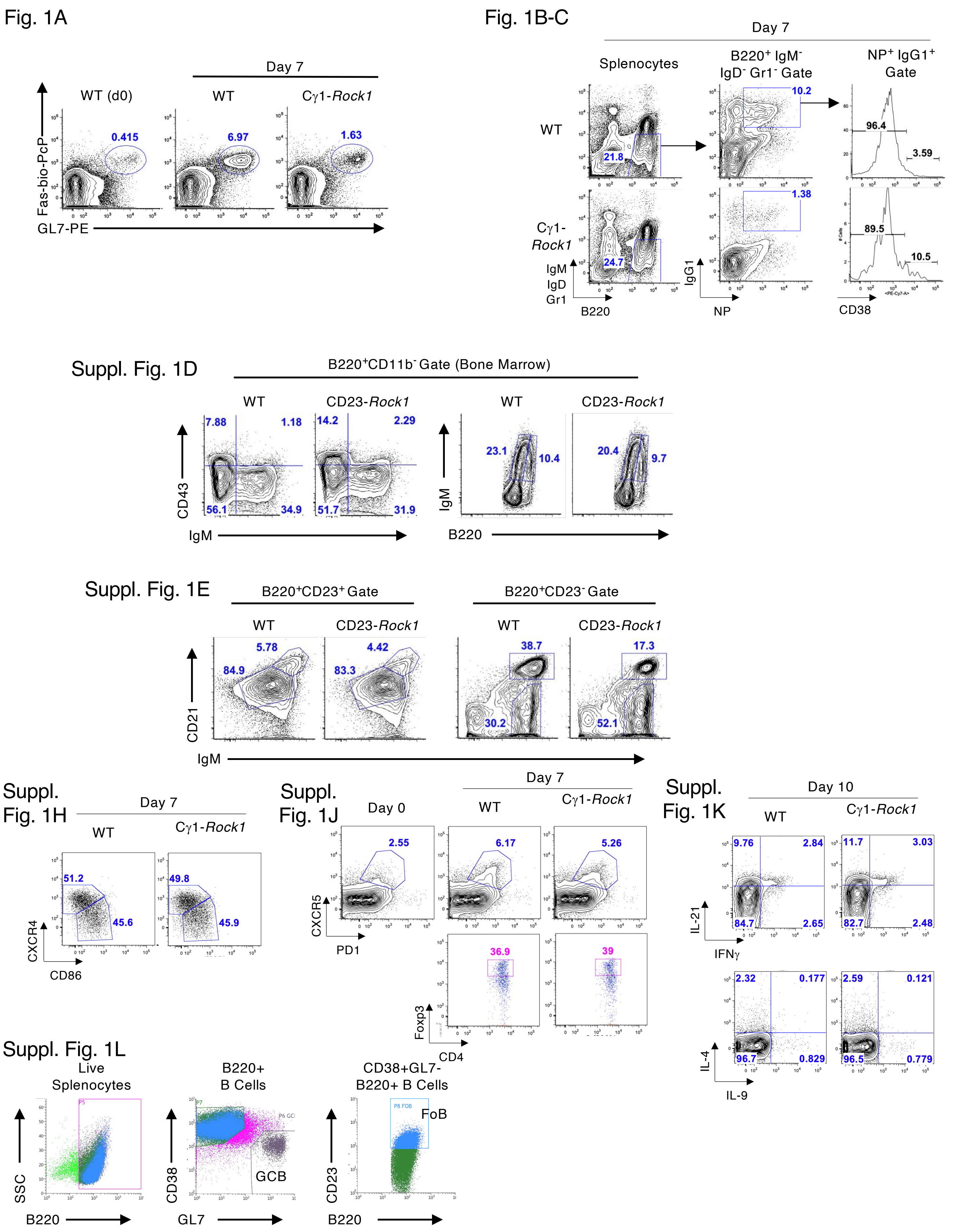

Fig. 2D

B-cells (CD19+)

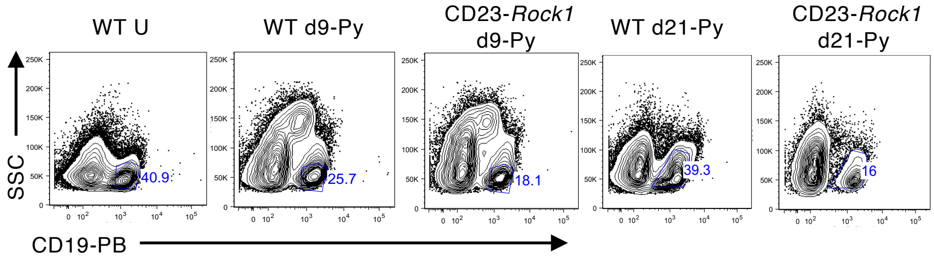

Fig. 2E

GC B-cells (CD19+ Fas+GL7+)

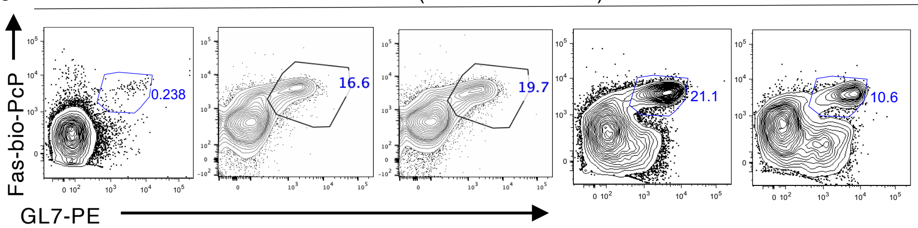

Fig. 2F

Atypical B-cells (CD19+ T-bet+CD11c+)

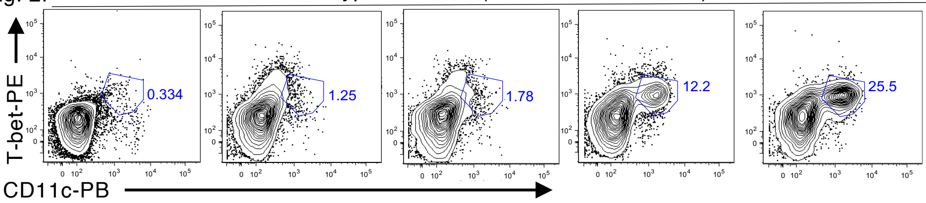

Fig. 2H

Plasmablasts/ Plasma cells (CD138+ B220int)

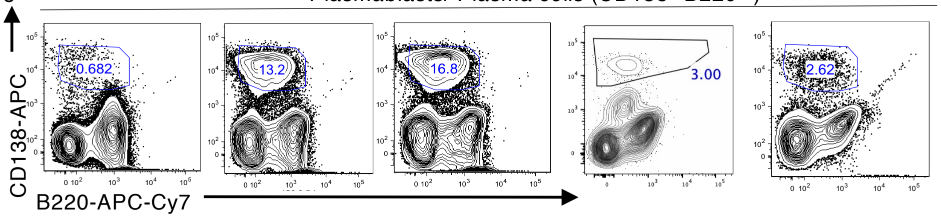

Suppl. Fig. 2B

CD4+ T-cells (CD3+CD4+)

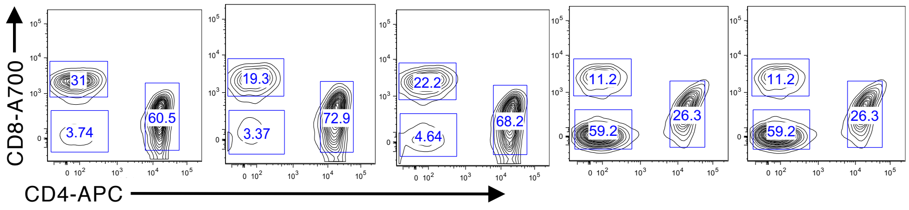

Suppl. Fig. 2C

Follicular T-cells (CD3+CD4+CD44+CXCR5+PD1+FOXP3-)

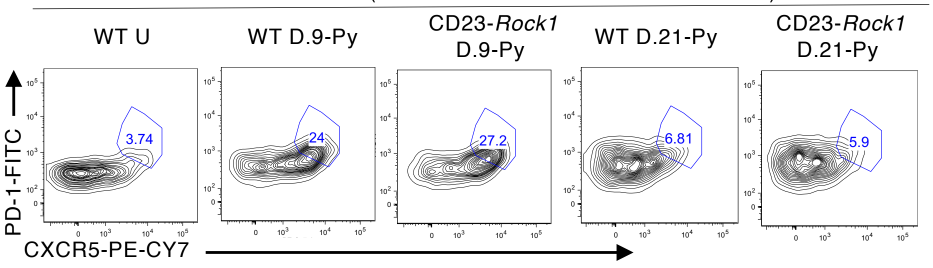

Suppl. Fig. 2E

IFN-γ+ CD4+ T-cells (CD3+CD4+IFN-γ+)

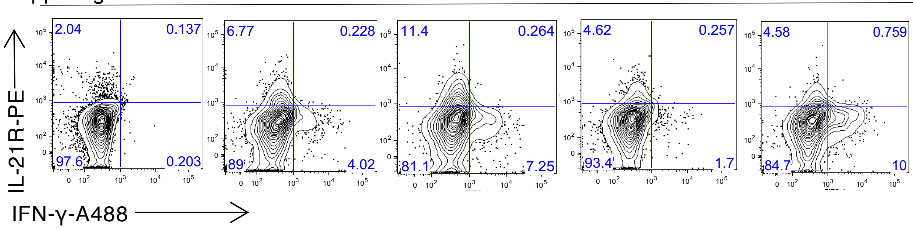

Suppl. Fig. 2I

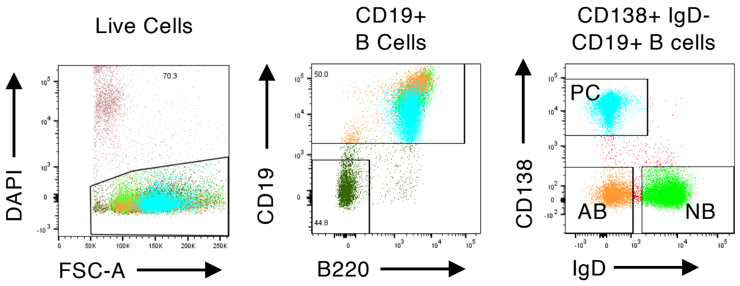

Suppl. Fig. 3K

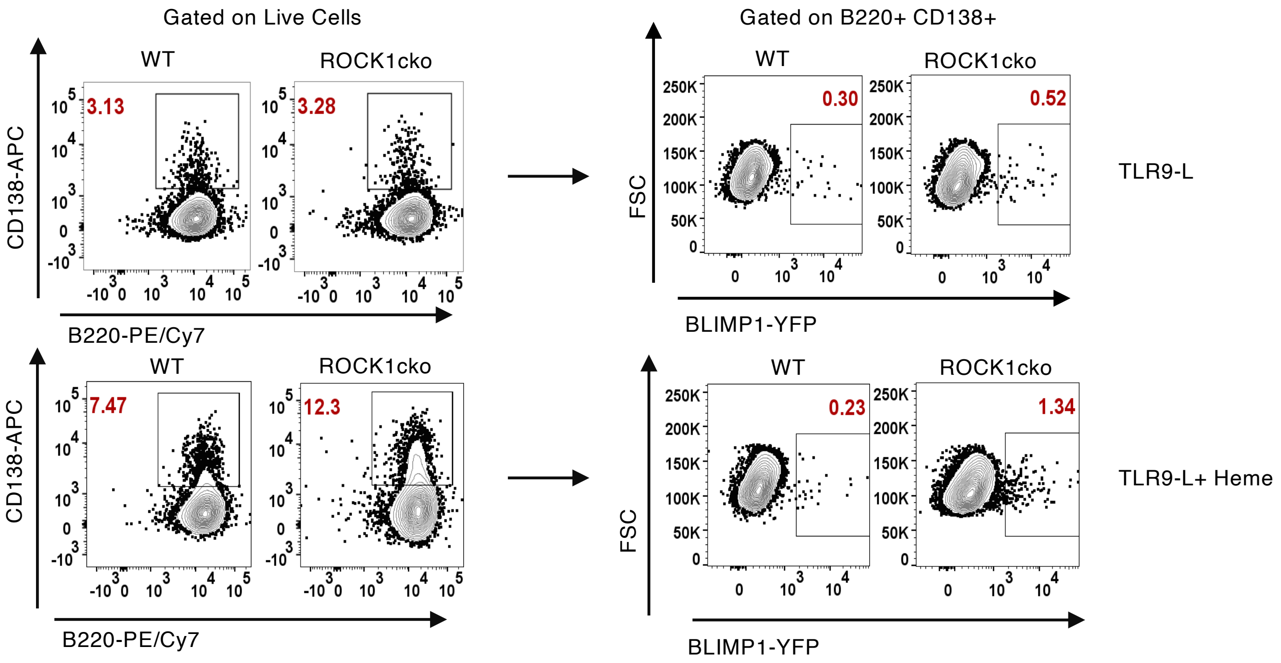

Supplement: Supplemental data [file jciinsight-10-180507-s039.pdf]
