# Supplementary material for: ROCK1 promotes B cell differentiation and proteostasis under stress through the heme-regulated proteins, BACH2 and HRI
Source: JCI Insight. 2025 Feb 4;10(5):e180507. doi: 10.1172/jci.insight.180507 (PMC11949073; doi:10.1172/jci.insight.180507)

FULL UNEDITED BLOT/GEL FOR SUPPL. FIG. 1B

ROCK1 IVK

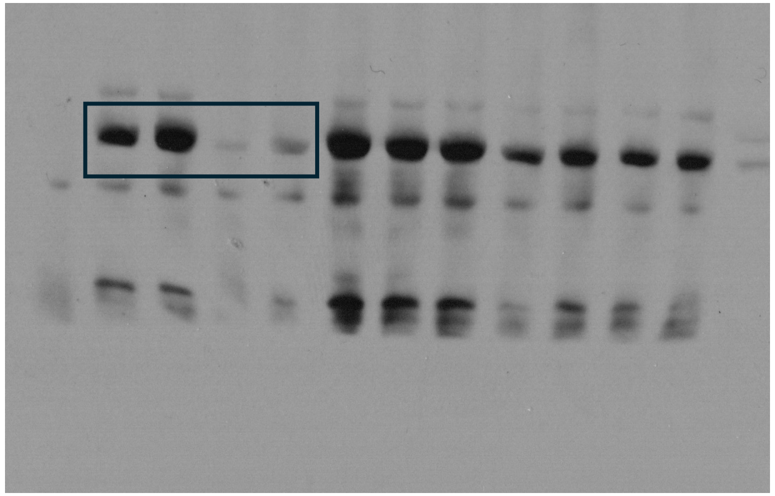

ROCK1 (INPUT)

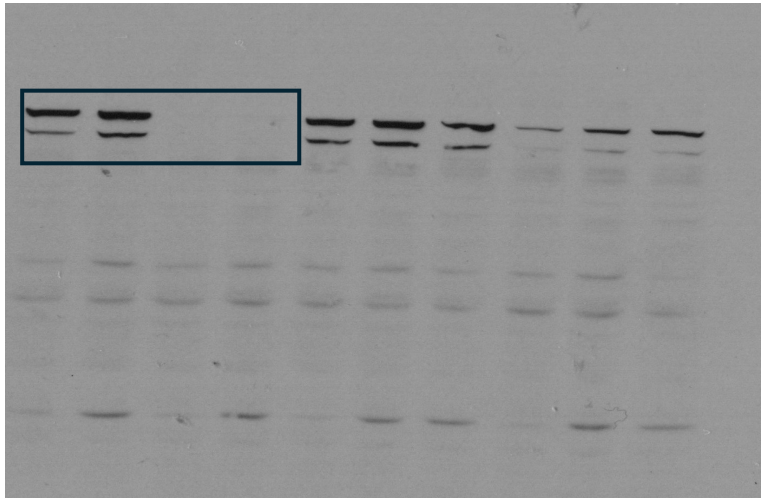

TUBULIN (INPUT)

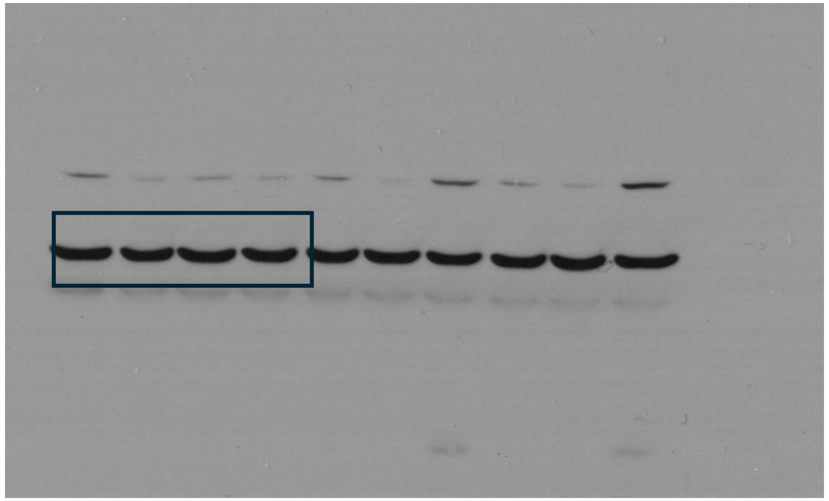

FULL UNEDITED BLOT/GEL FOR SUPPL. FIG. 1C

ROCK2 IVK

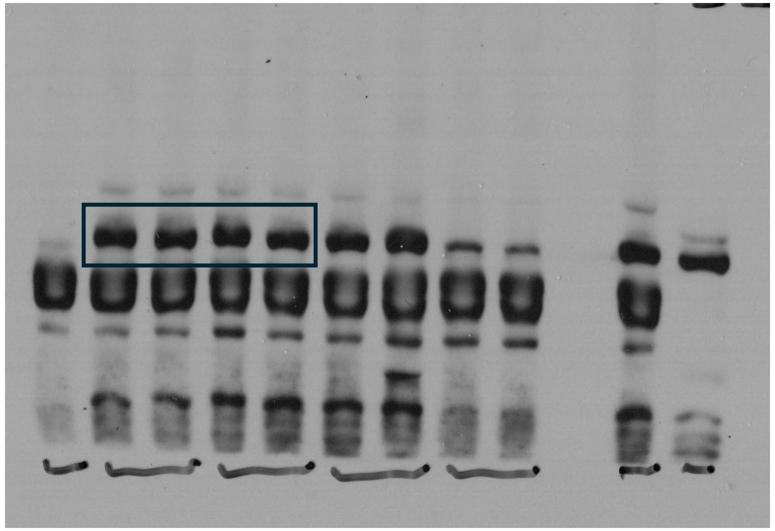

ROCK2 (INPUT)

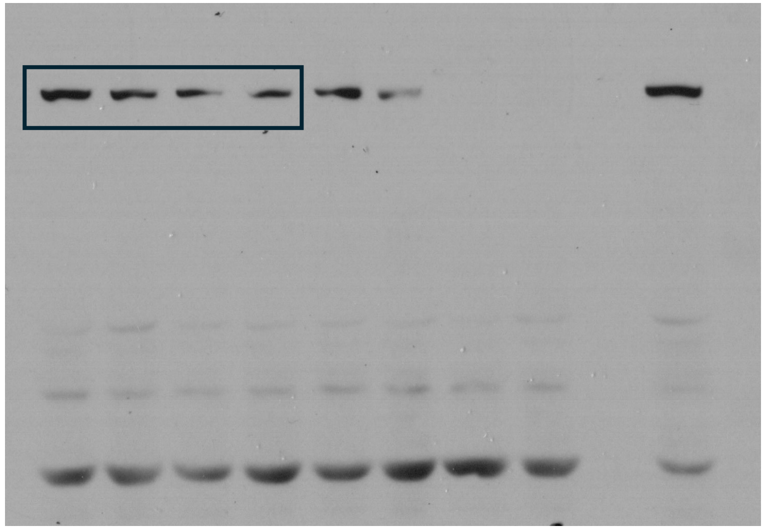

TUBULIN (INPUT)

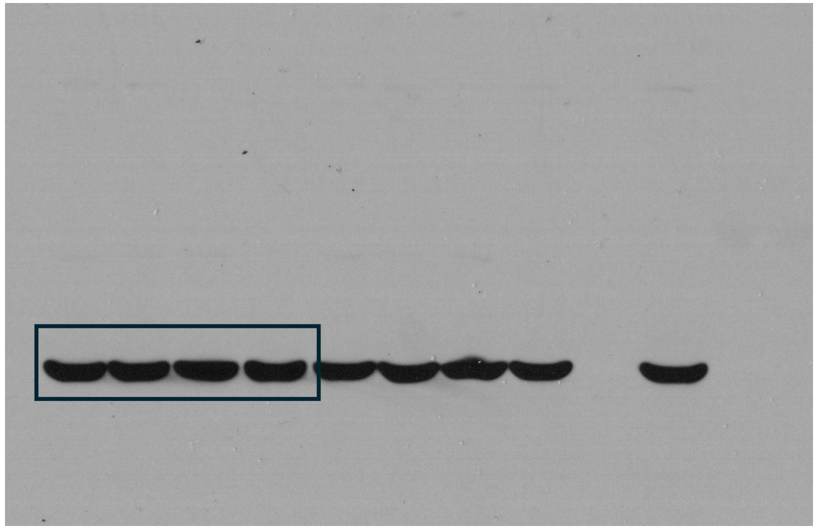

FULL UNEDITED BLOT/GEL FOR FIG. 2A

ROCK1 IVK ROCK2 IVK

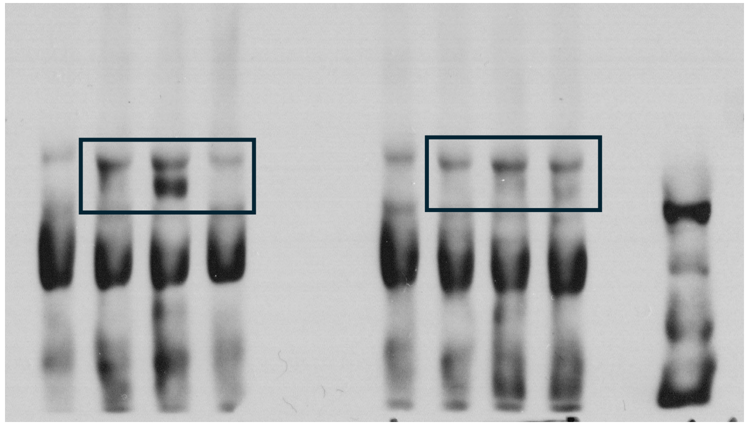

ROCK1 (INPUT)

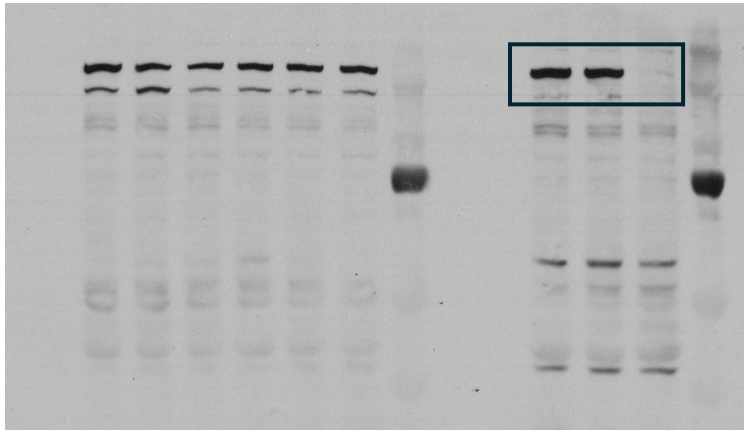

ROCK2 (INPUT)

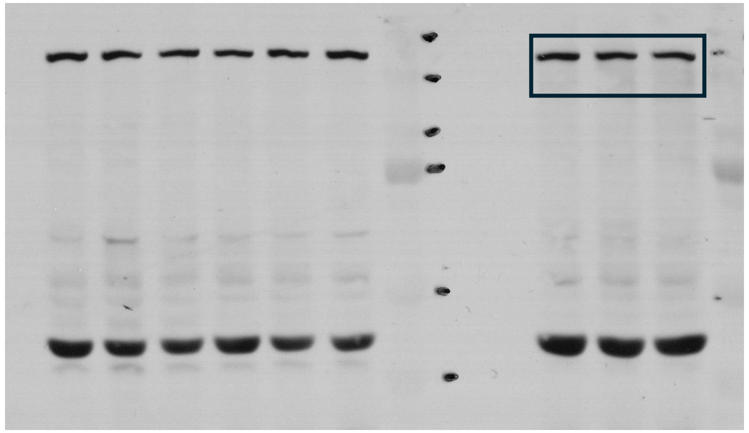

FULL UNEDITED BLOT/GEL FOR FIG. 3B

BACH2

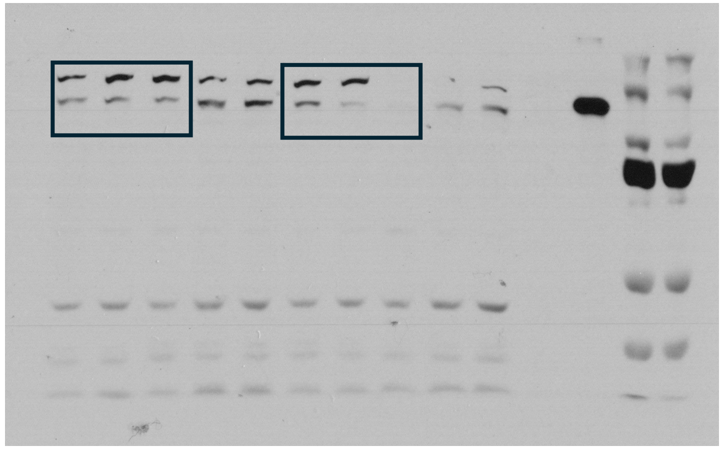

IRF4

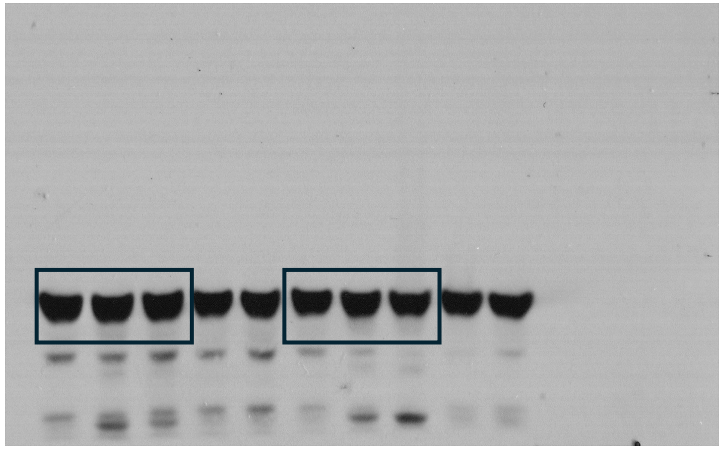

TUBULIN

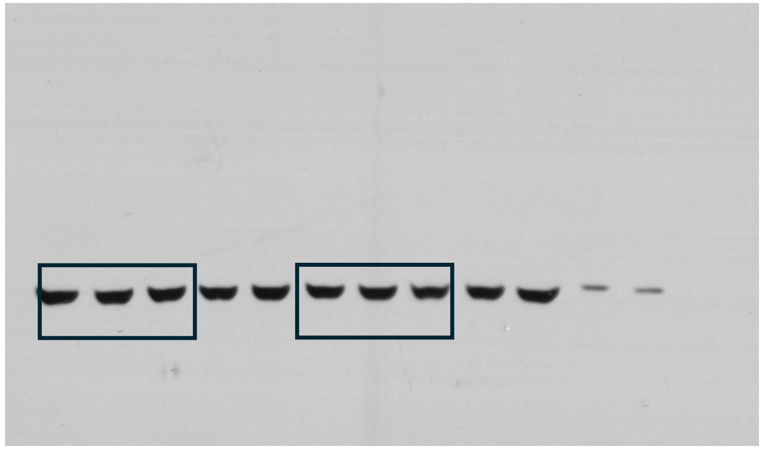

FULL UNEDITED BLOT/GEL FOR FIG. 3D

Phospho-S/T

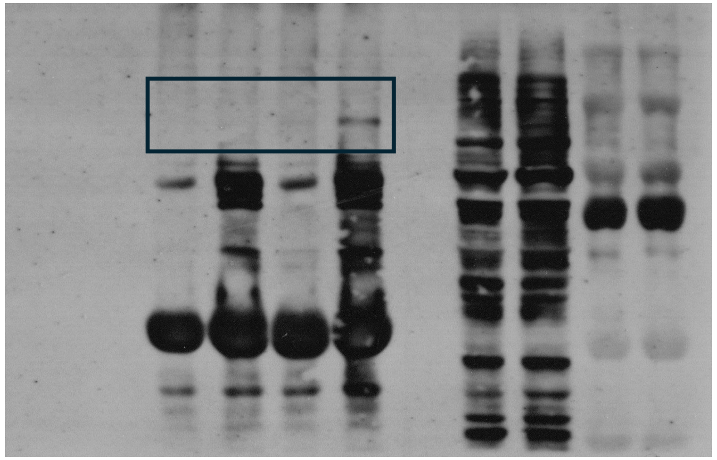

BACH2

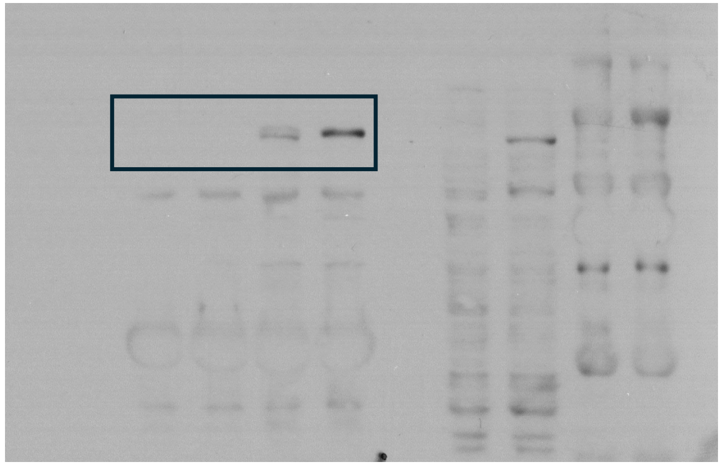

FULL UNEDITED BLOT/GEL FOR FIG. 3F

FLAG

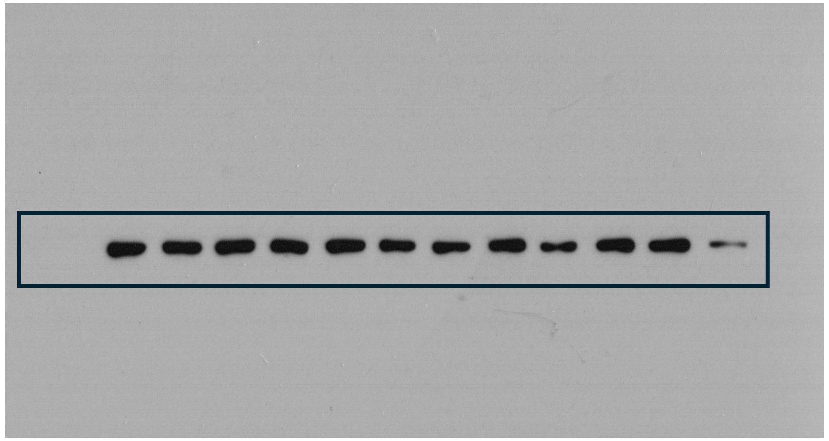

TUBULIN

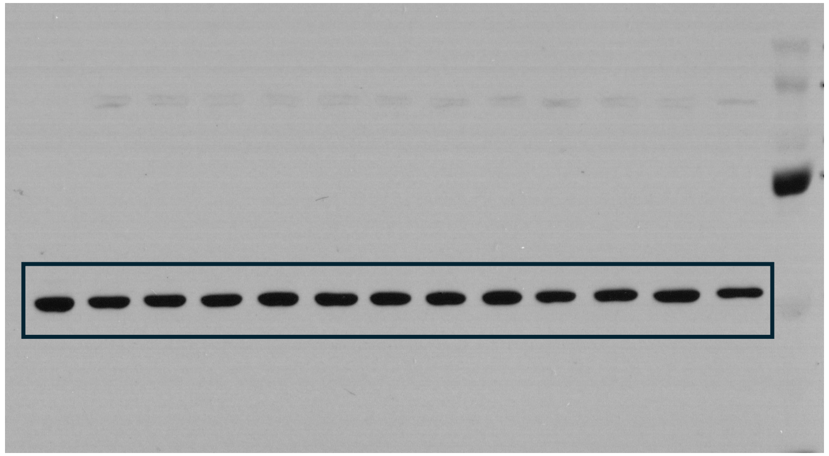

FULL UNEDITED BLOT/GEL FOR SUPPL. FIG. 3D

BACH2

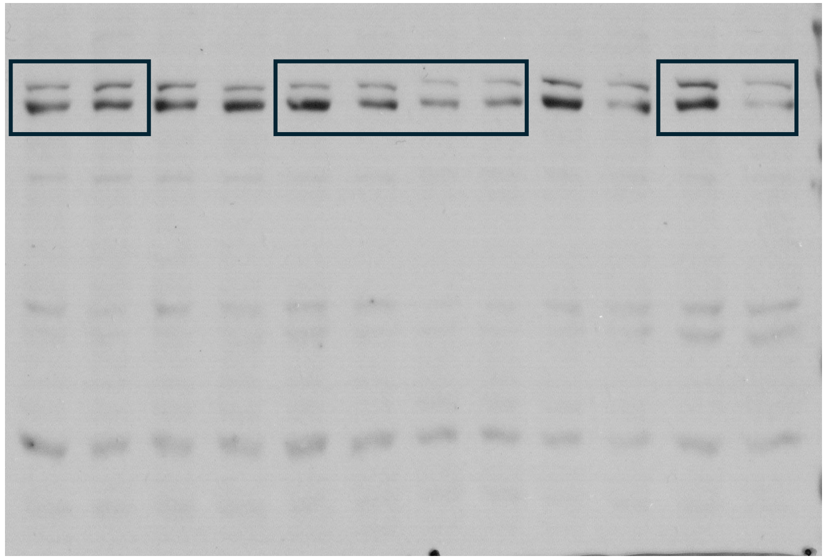

HDAC1

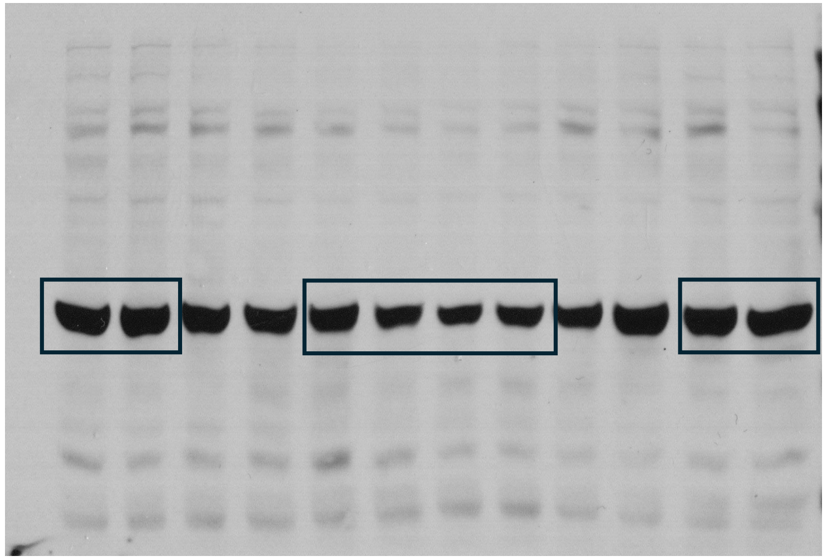

HDAC1

FULL UNEDITED BLOT/GEL FOR FIG. 6A

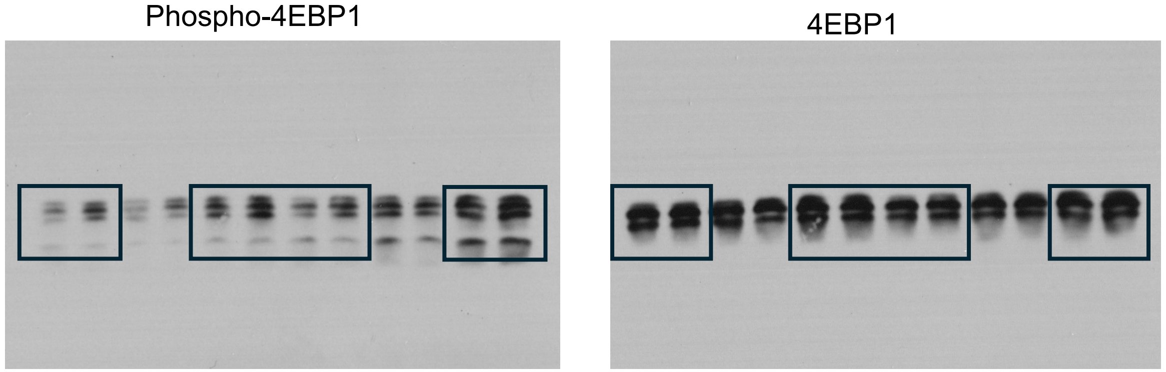

FULL UNEDITED BLOT/GEL FOR FIG. 6B

RAPTOR (LEFT PANEL)

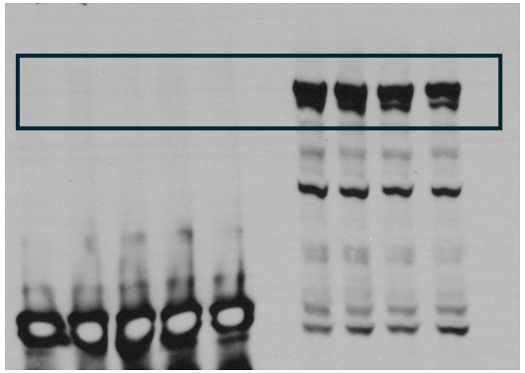

RAPTOR (RIGHT PANEL)

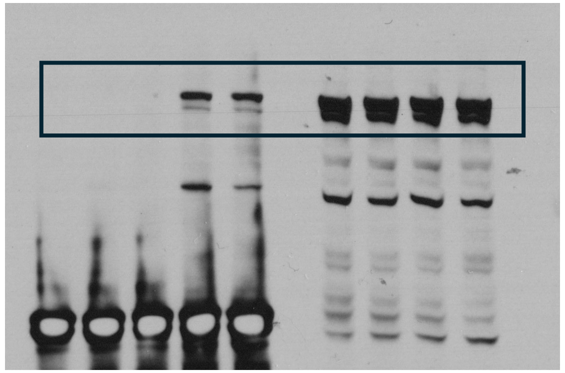

TRAF6 (LEFT PANEL)

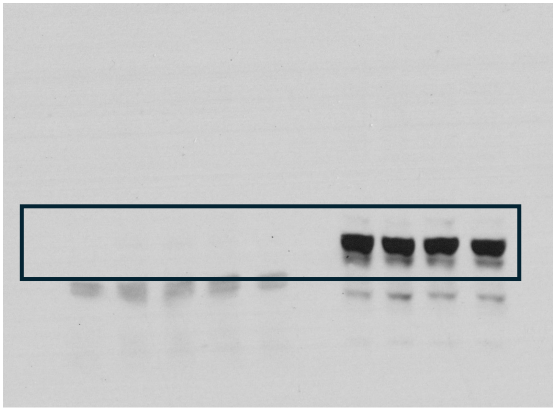

TRAF6 (RIGHT PANEL)

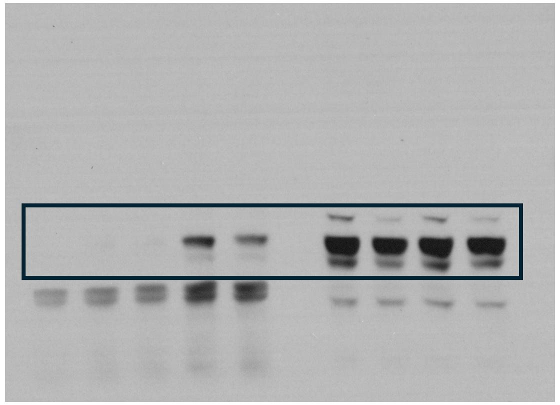

FULL UNEDITED BLOT/GEL FOR FIG. 6B CONT'D

Phospho-p62 (S349) (LEFT PANEL)

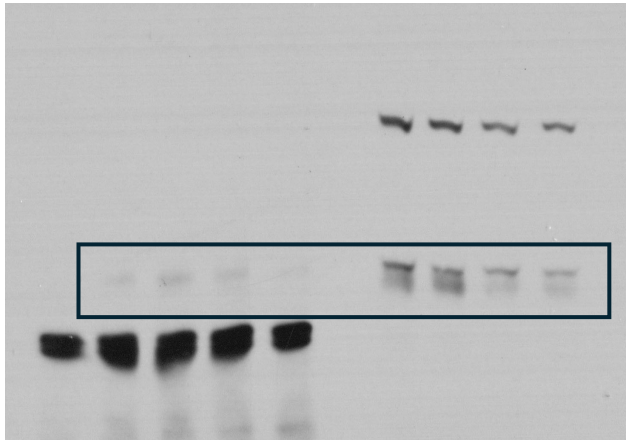

Phospho-p62 (S349) (RIGHT PANEL)

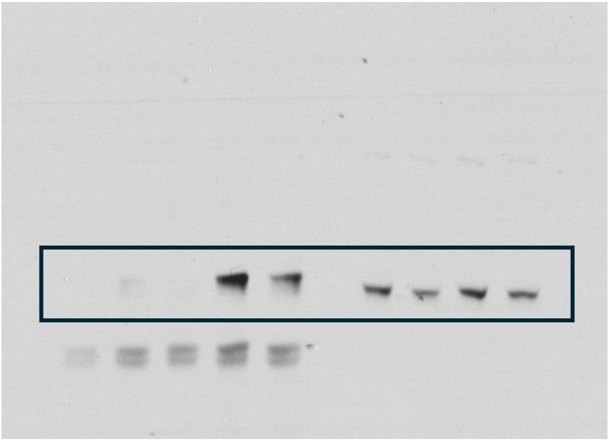

p62 (LEFT PANEL)

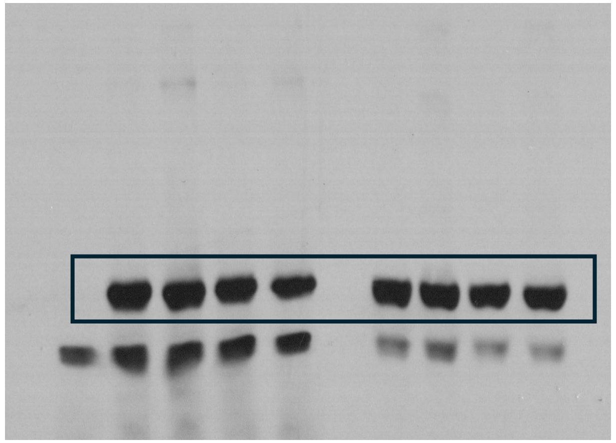

p62 (RIGHT PANEL)

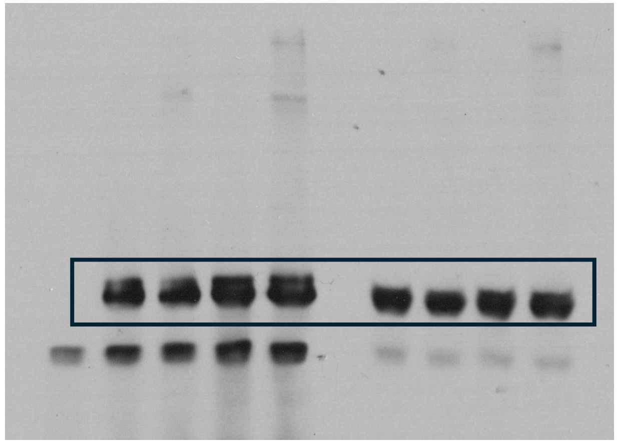

KEAP1 (LEFT PANEL)

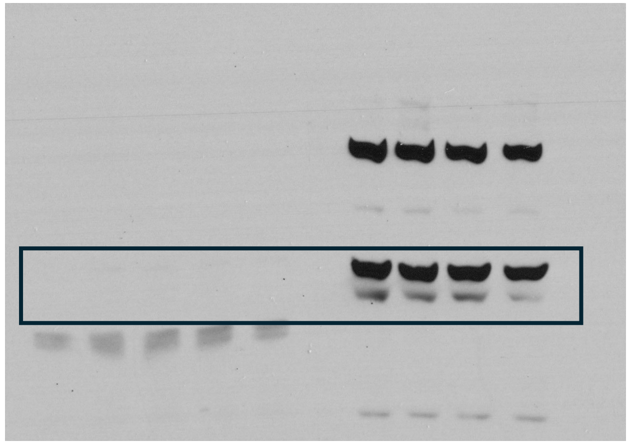

KEAP1 (RIGHT PANEL)

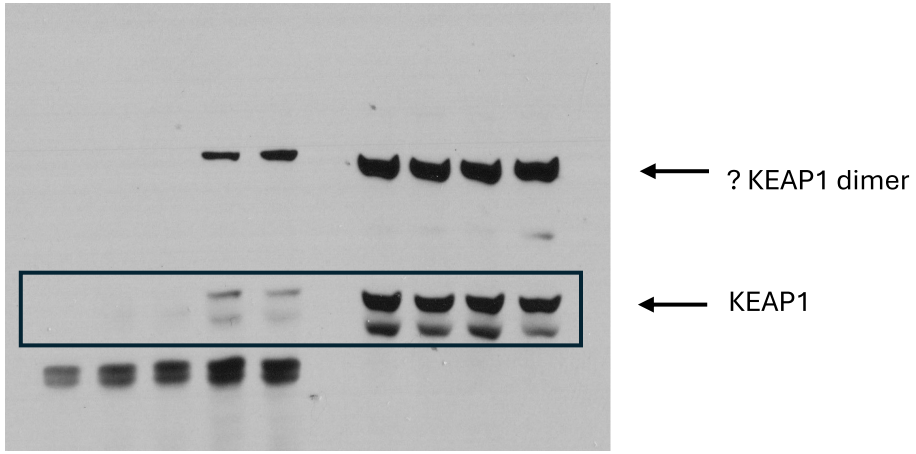

FULL UNEDITED BLOT/GEL FOR FIG. 6C

PHOSPHO-TBK1 (S172)

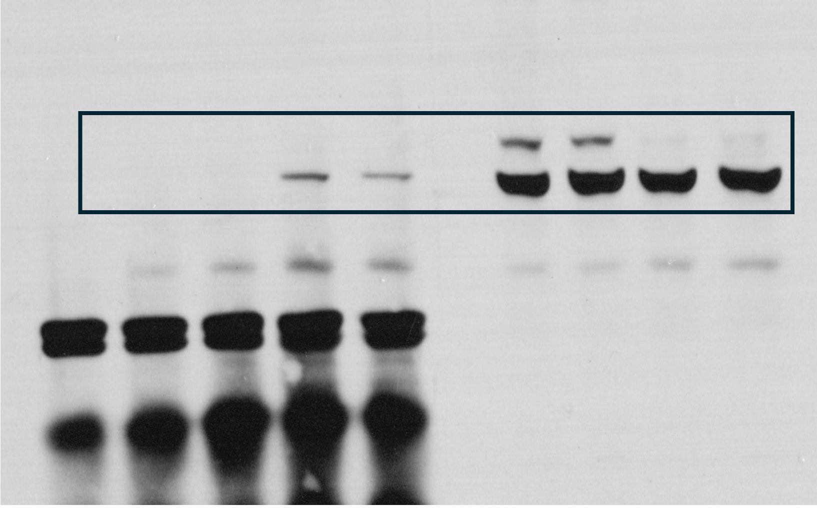

TBK1

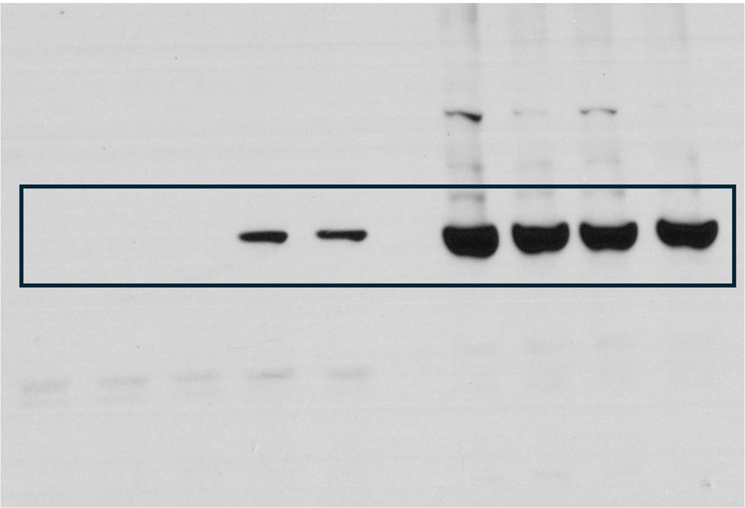

RIPK1

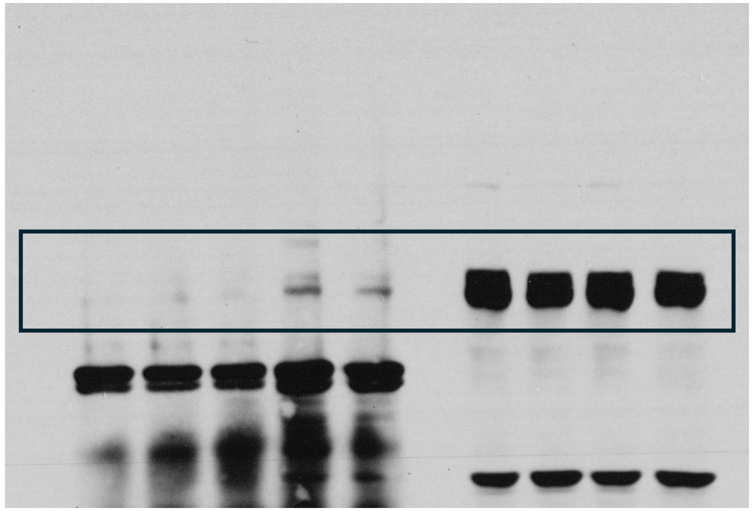

RIPK3

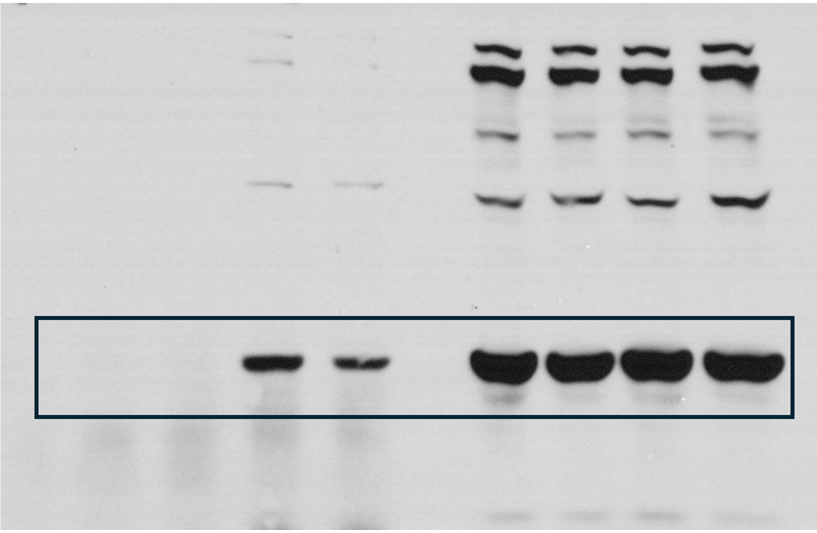

p62

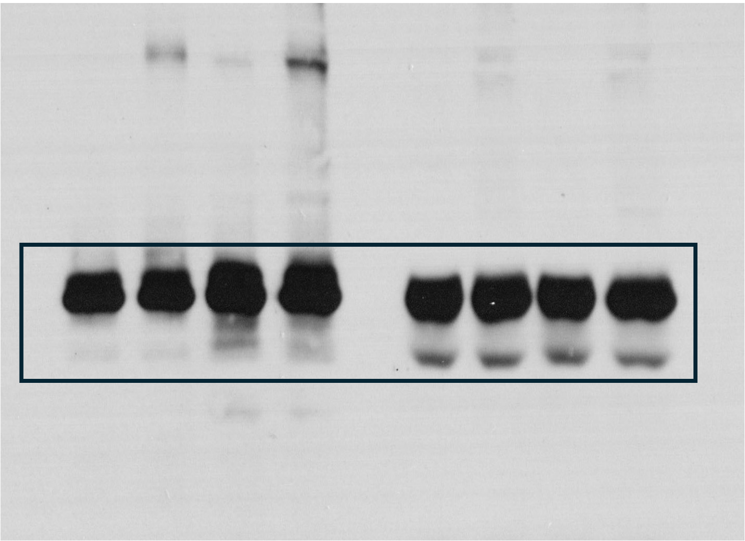

FULL UNEDITED BLOT/GEL FOR FIG. 6D

ZBP1

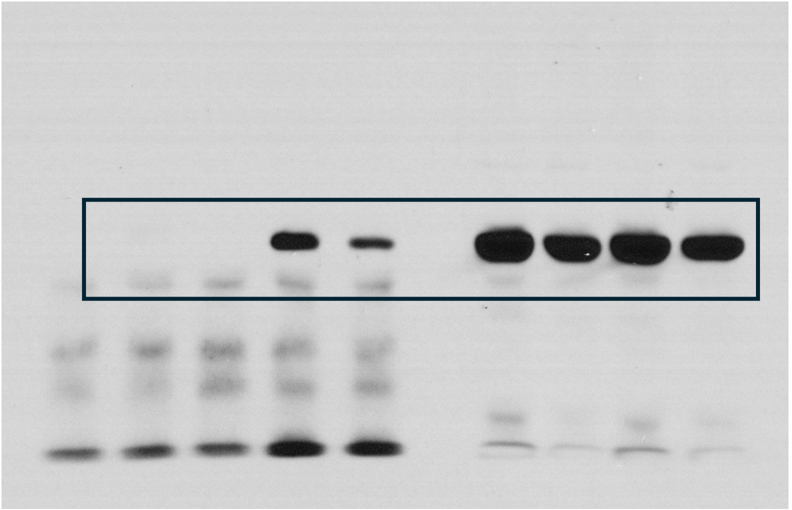

p62

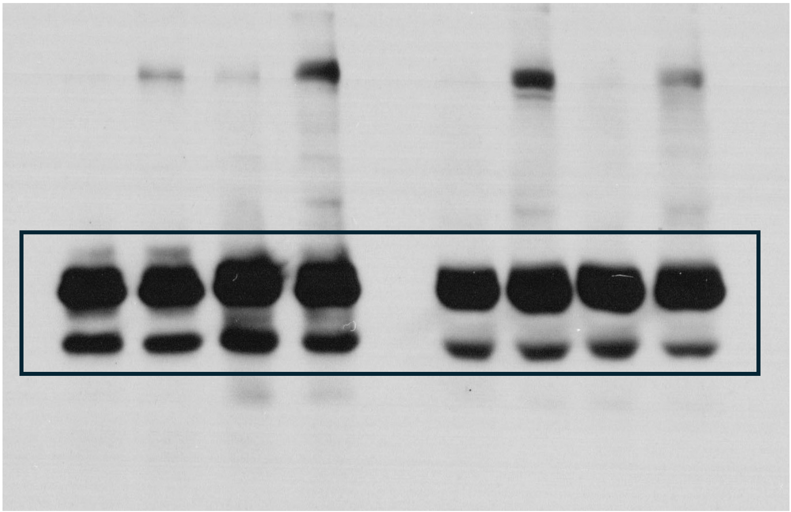

FULL UNEDITED BLOT/GEL FOR FIG. 6E

PLK1

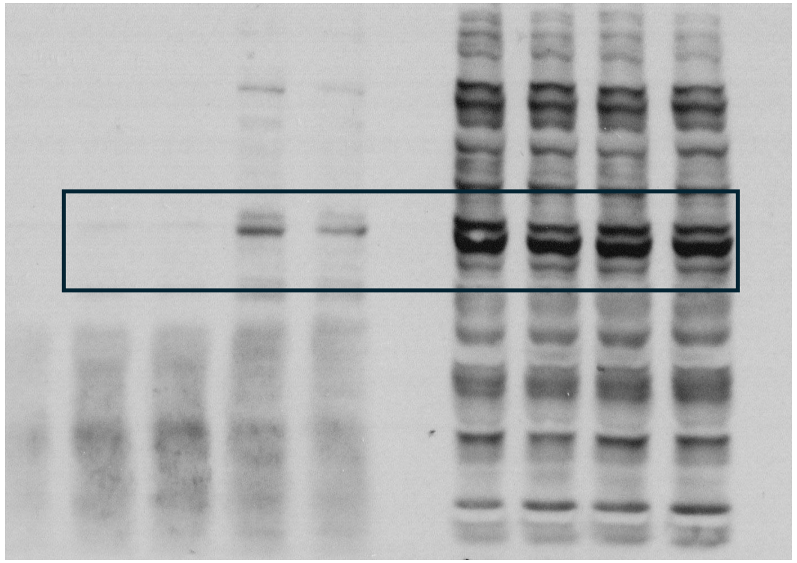

p62

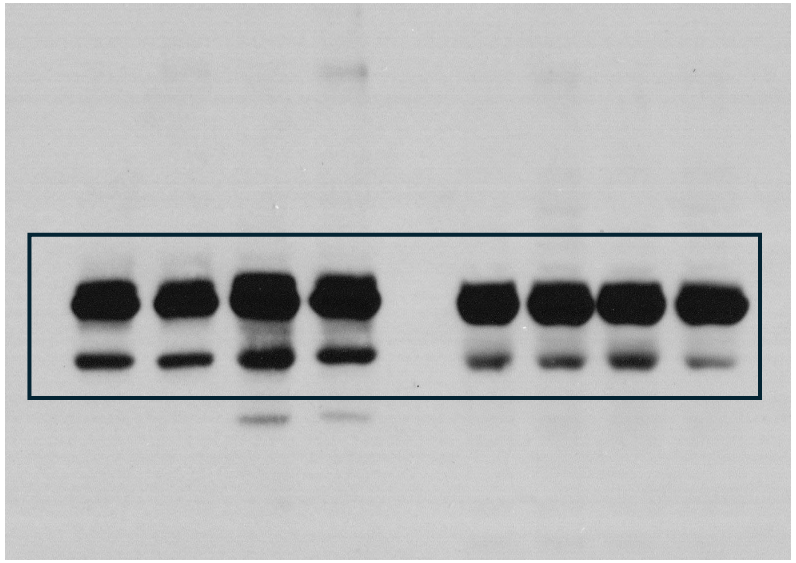

FULL UNEDITED BLOT/GEL FOR FIG. 6F

p62 (LEFT PANEL)

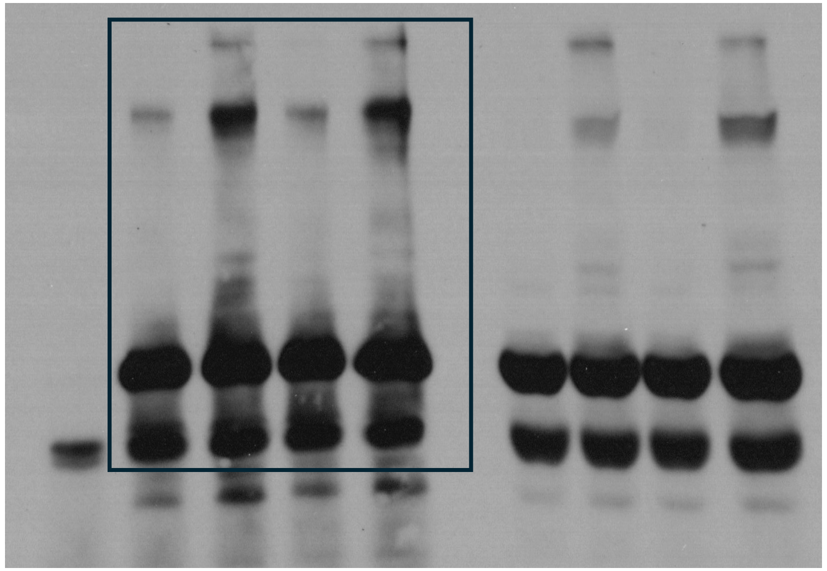

p62 (RIGHT PANEL)

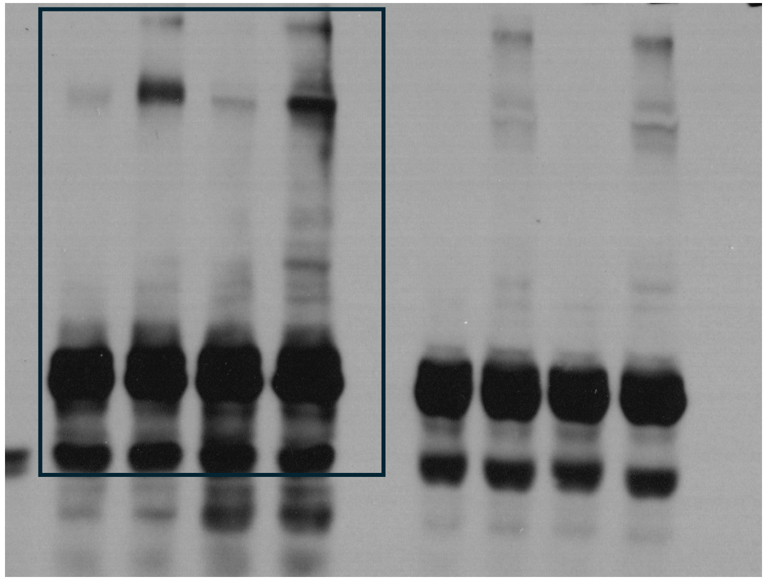

FULL UNEDITED BLOT/GEL FOR FIG. 6G

SOD1

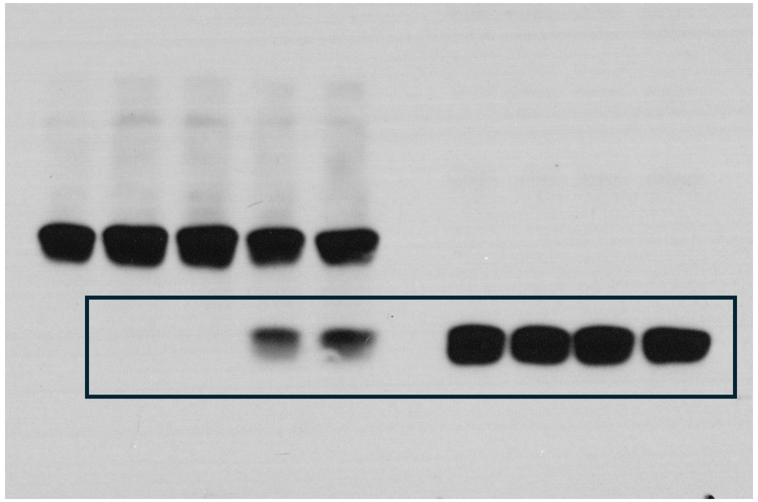

TDP-43

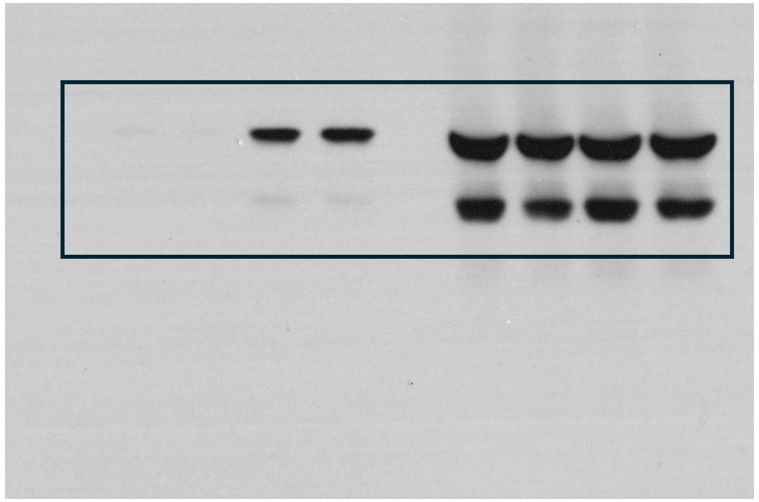

p62

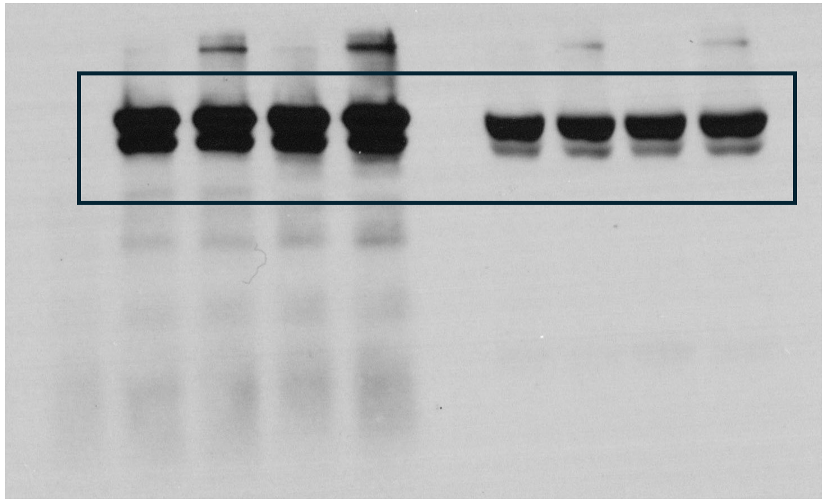

FULL UNEDITED BLOT/GEL FOR FIG. 6H

C9ORF72

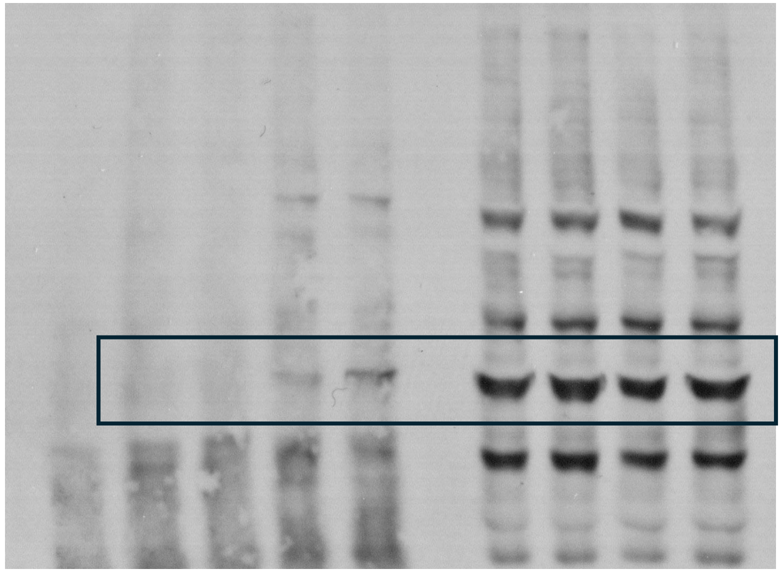

p62

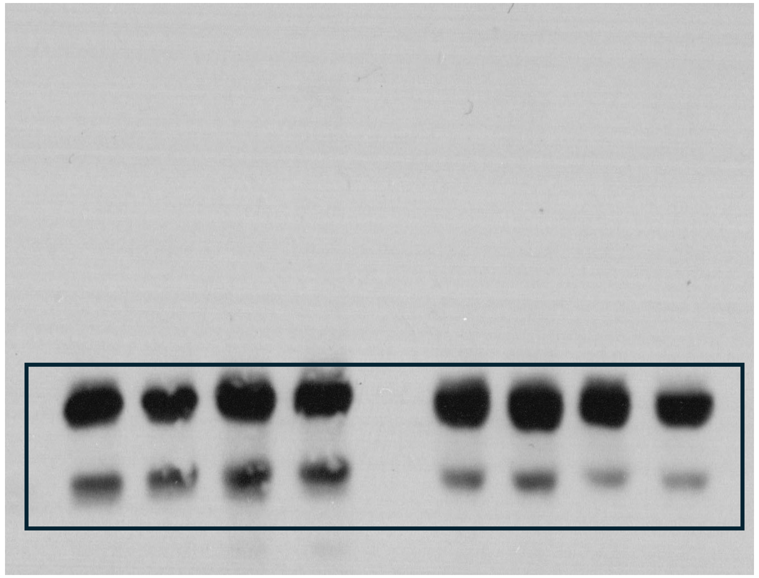

FULL UNEDITED BLOT/GEL FOR FIG. 6I

LC3A/B

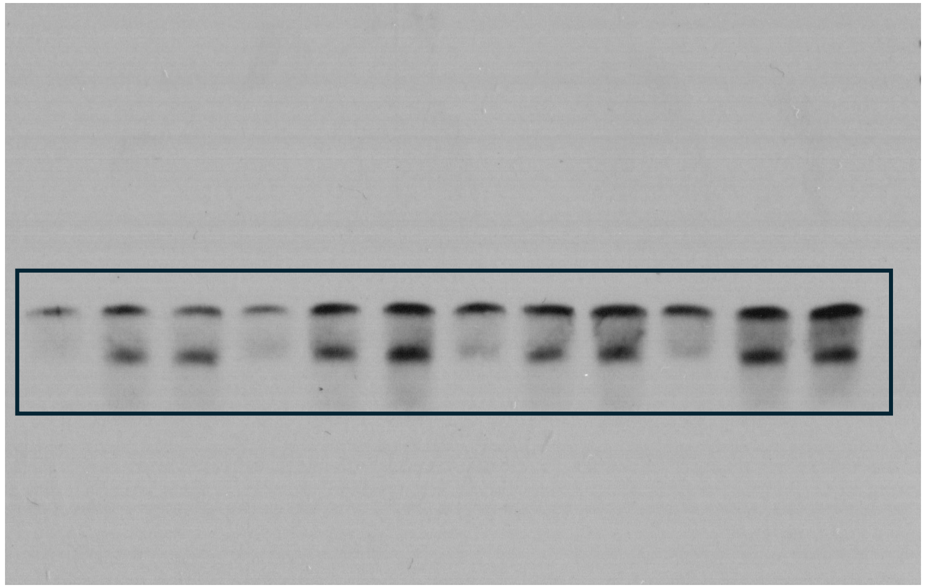

Tubulin

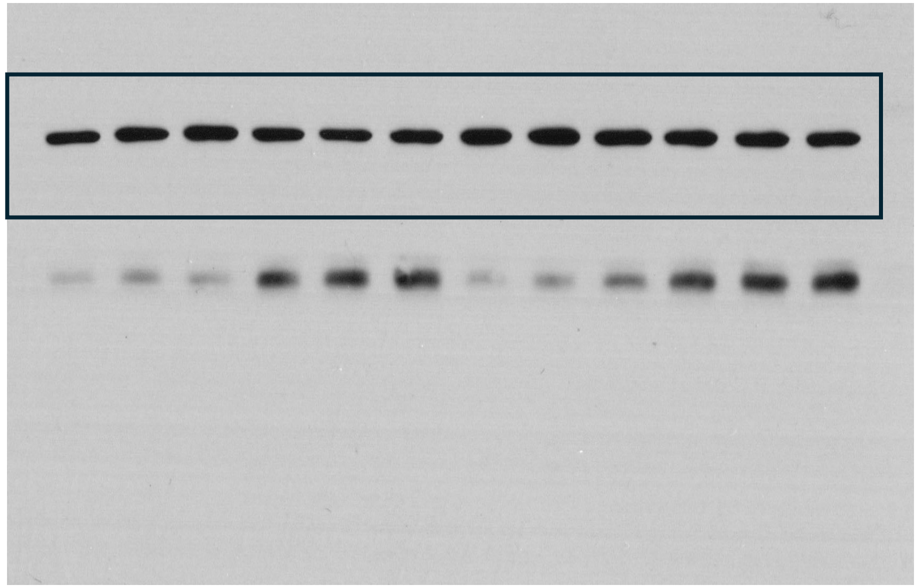

FULL UNEDITED BLOT/GEL FOR SUPPL. FIG. 6A

phospho-pS6 (S240/244)

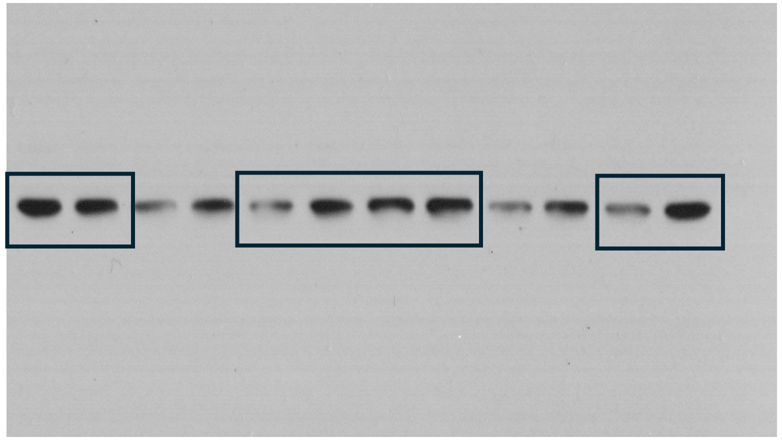

TUBULIN

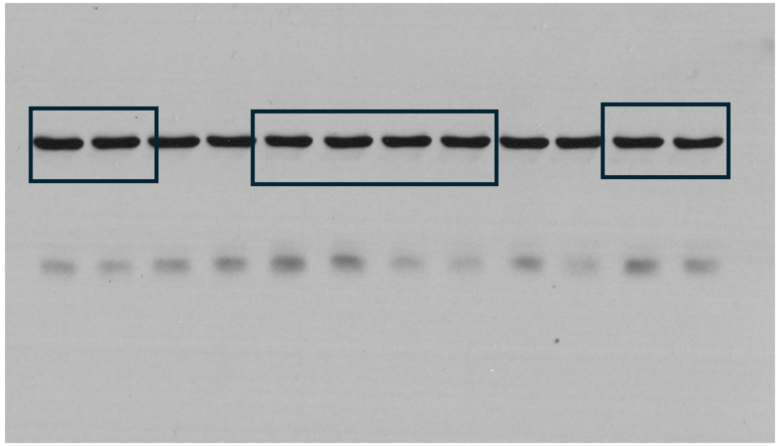

FULL UNEDITED BLOT/GEL FOR SUPPL. FIG. 6B

phospho-p70S6K (T389)

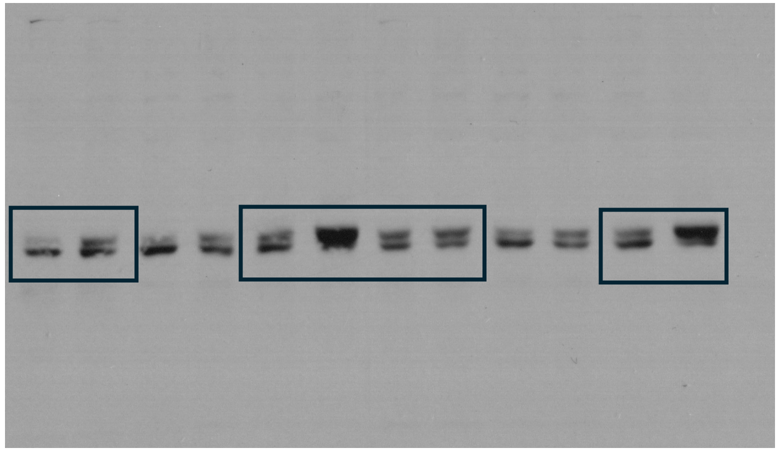

TUBULIN

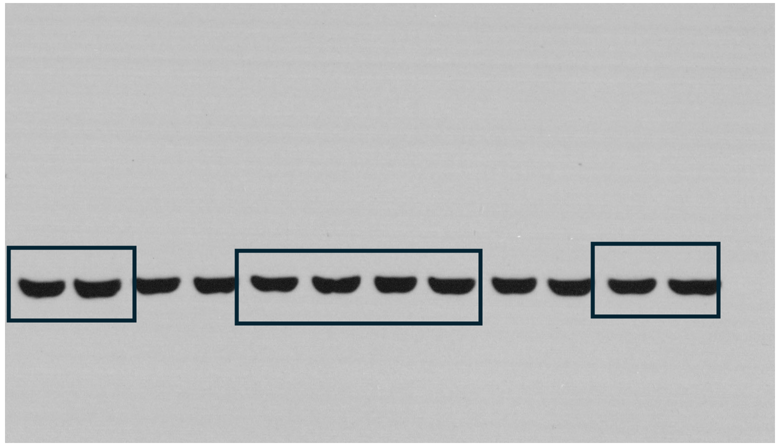

FULL UNEDITED BLOT/GEL FOR SUPPL. FIG. 6C

phospho-ULK1 (S757)

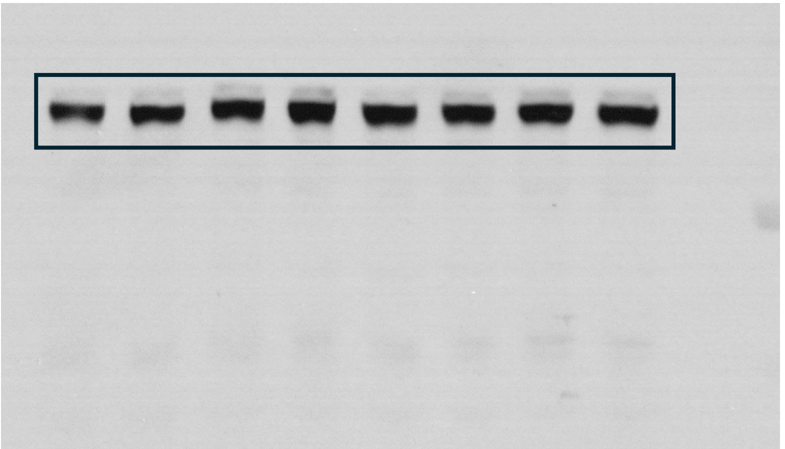

TUBULIN

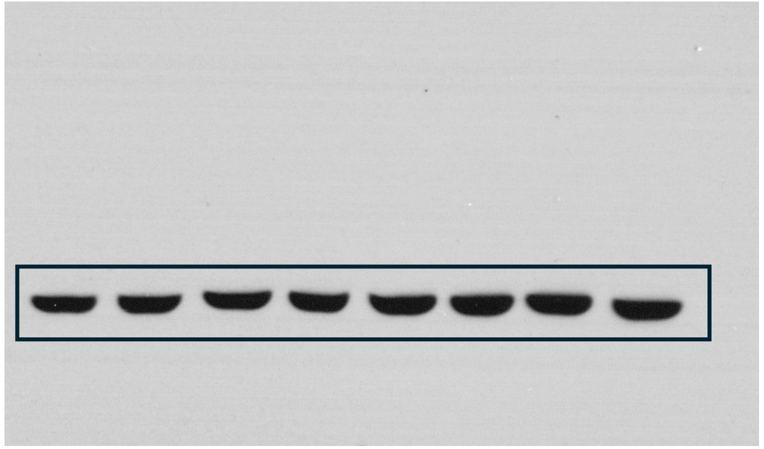

FULL UNEDITED BLOT/GEL FOR SUPPL. FIG. 6D

phospho-AKT (T308)

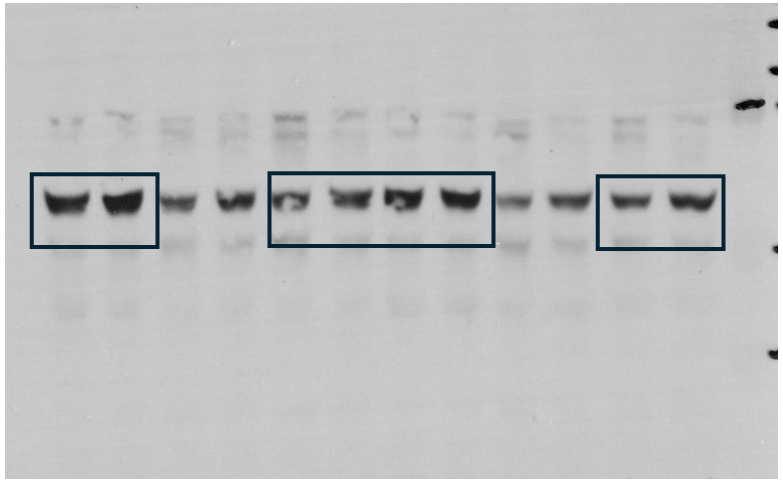

AKT

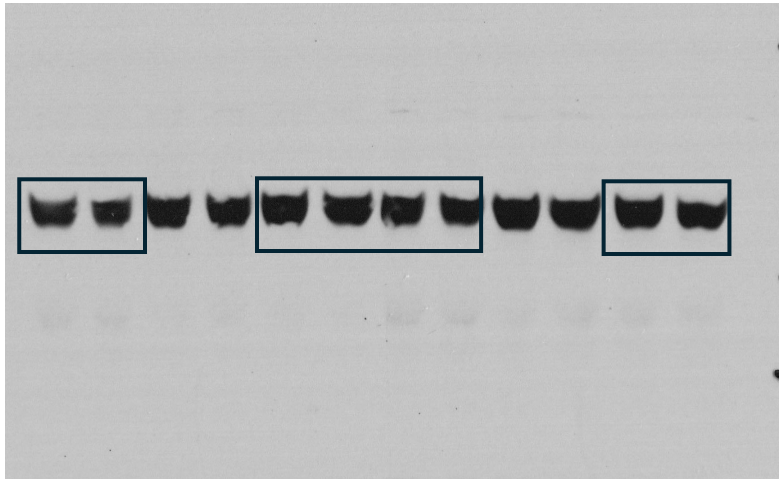

FULL UNEDITED BLOT/GEL FOR SUPPL. FIG. 6E

phospho-AKT (S473)

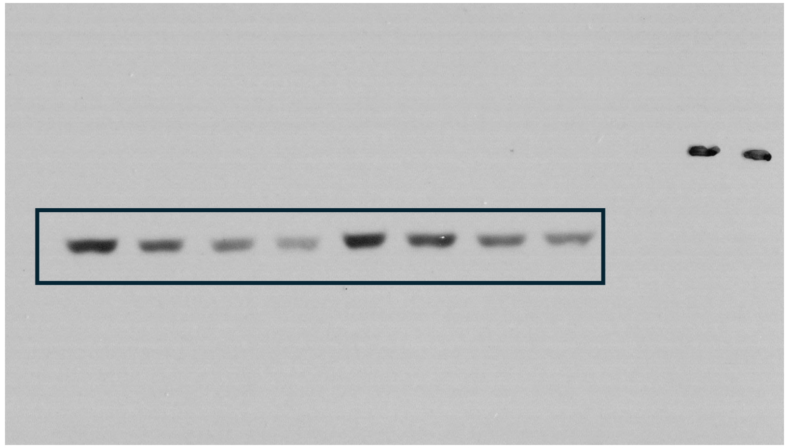

AKT

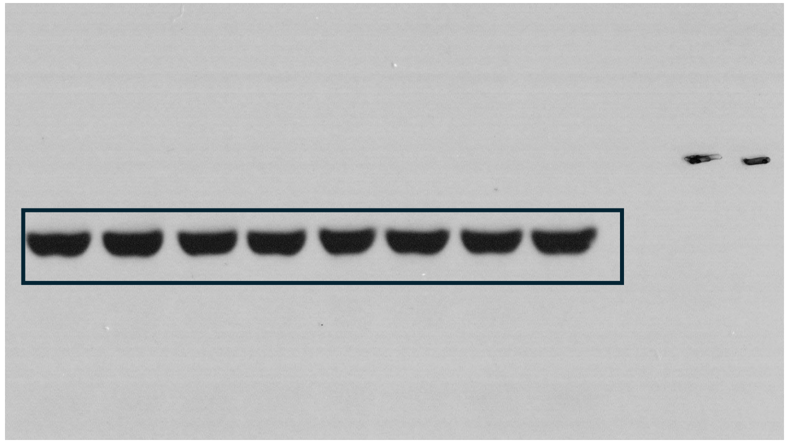

FULL UNEDITED BLOT/GEL FOR SUPPL. FIG. 6F

phospho-AMPKα (T172)

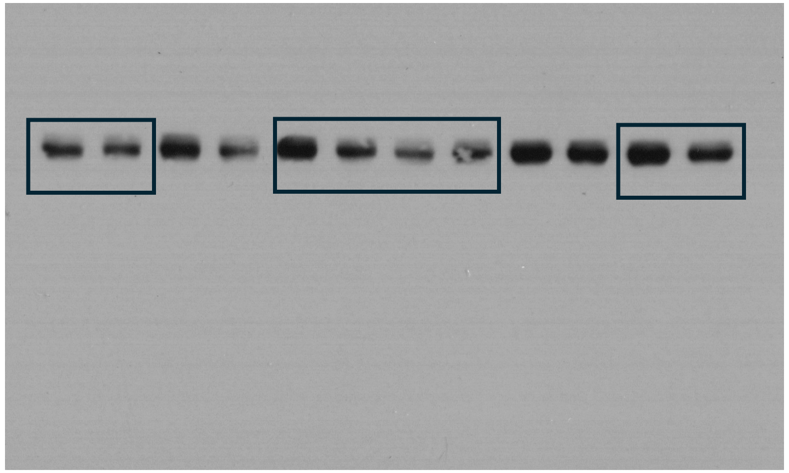

TUBULIN

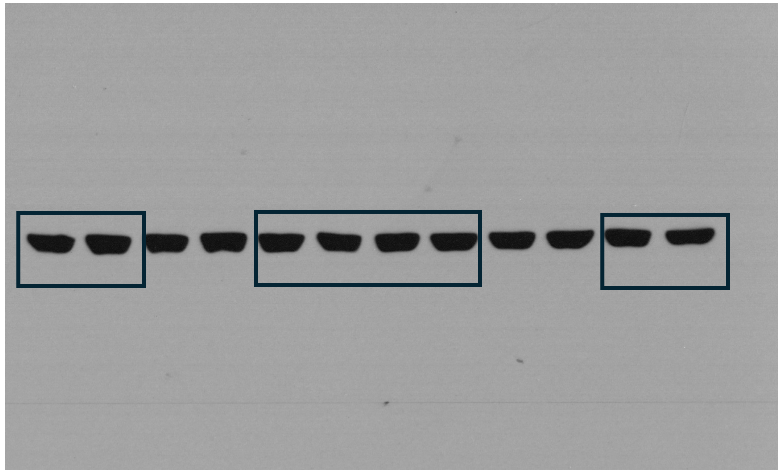

FULL UNEDITED BLOT/GEL FOR SUPPL. FIG. 6G

phospho-4EBP1

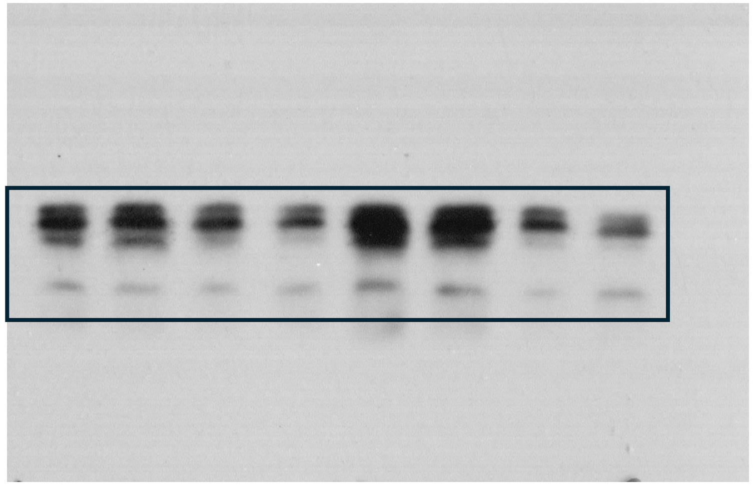

4EBP1

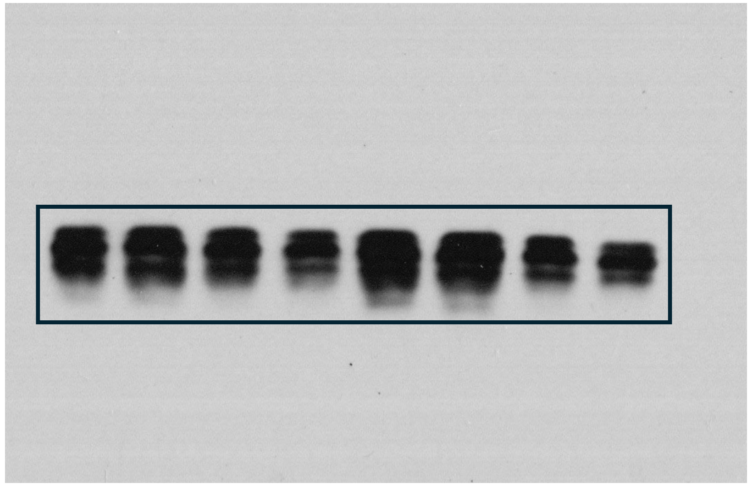

FULL UNEDITED BLOT/GEL FOR SUPPL. FIG. 6H

PHOSPHO-TBK1(S172)

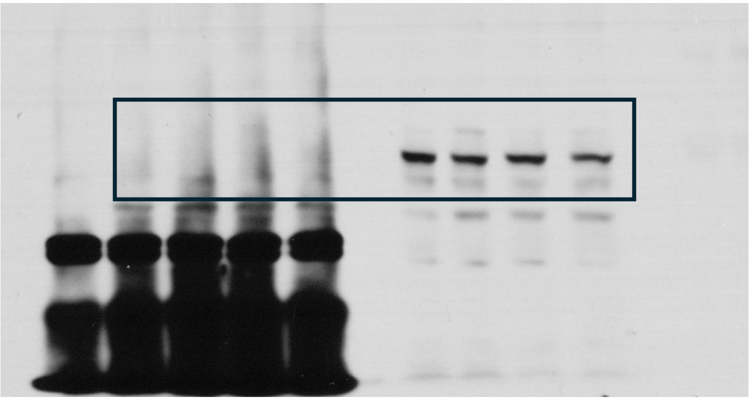

TBK1

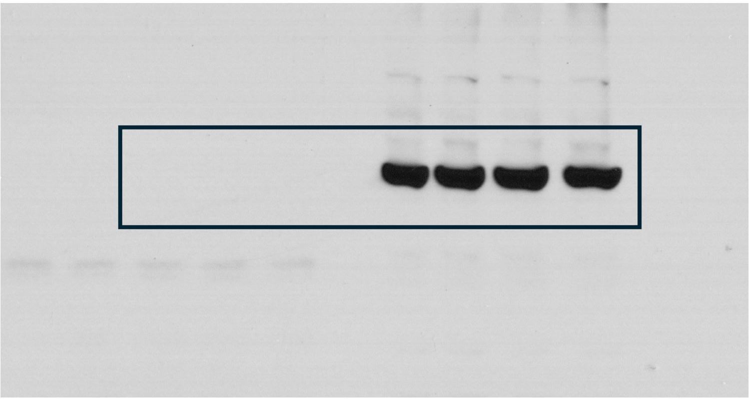

RIPK1

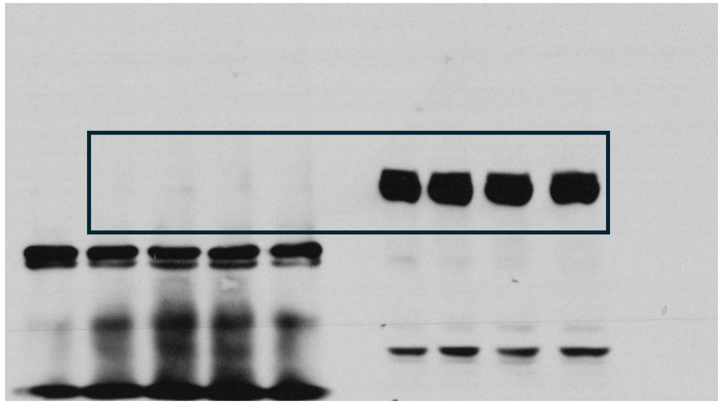

RIPK3

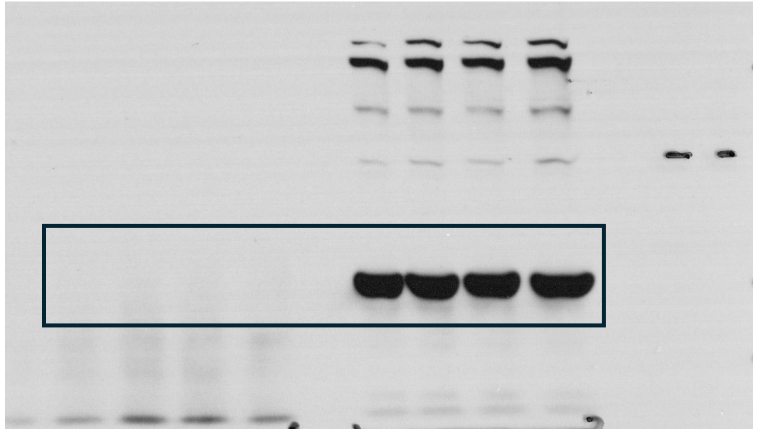

p62

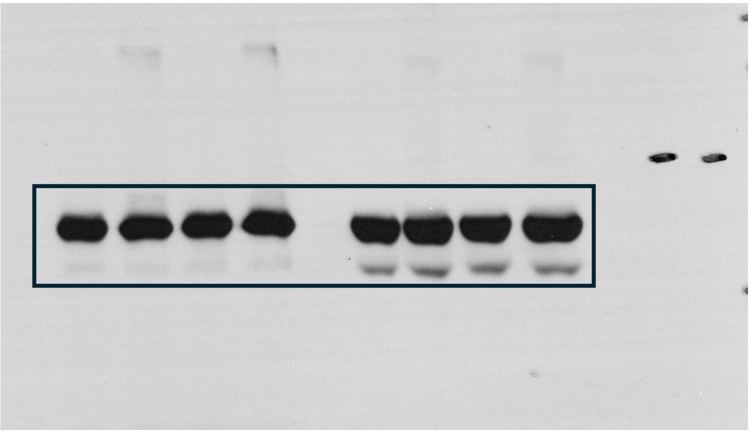

FULL UNEDITED BLOT/GEL FOR SUPPL. FIG. 6I

ZBP1

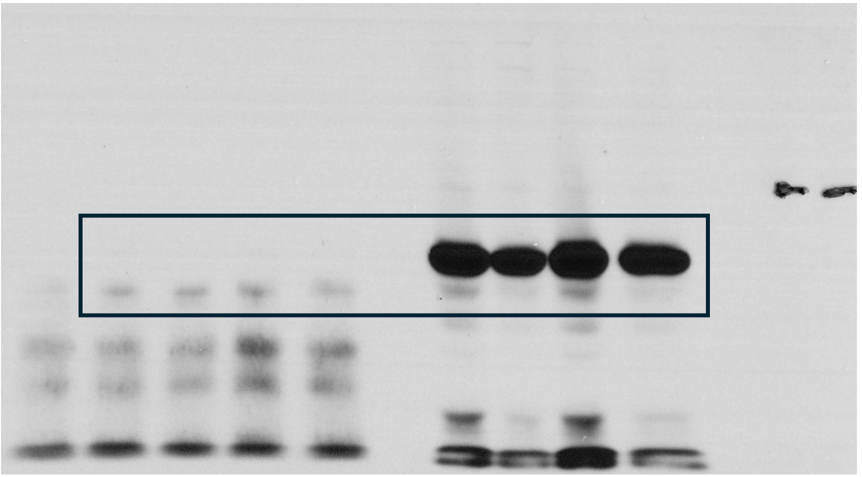

p62

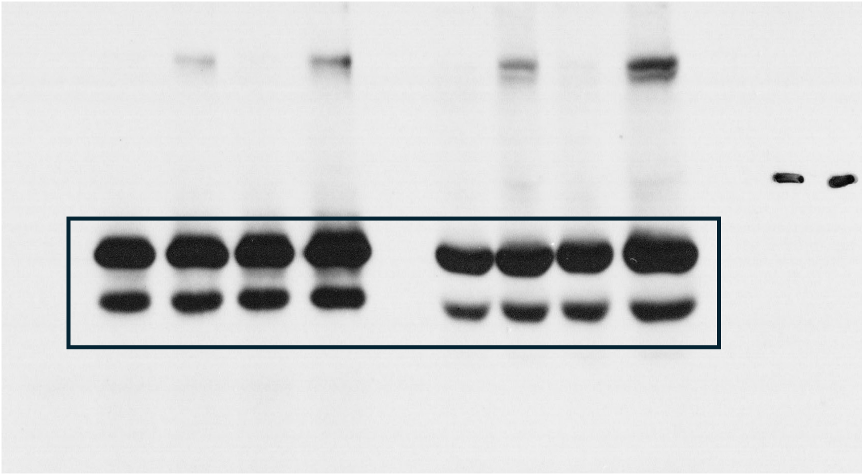

FULL UNEDITED BLOT/GEL FOR SUPPL. FIG. 6J

MLKL

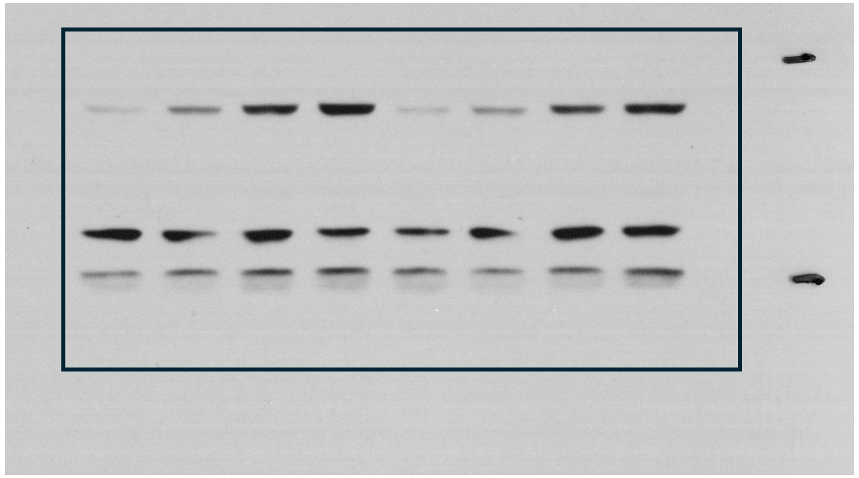

TUBULIN

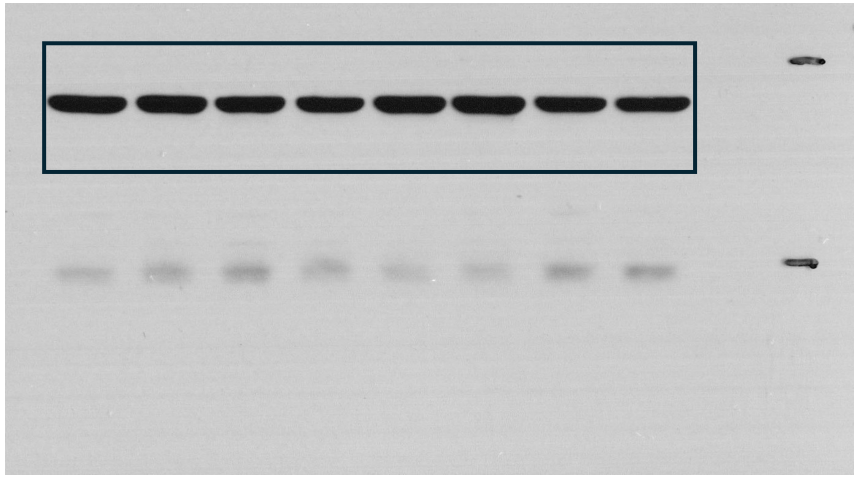

FULL UNEDITED BLOT/GEL FOR SUPPL. FIG. 6L

PLK1

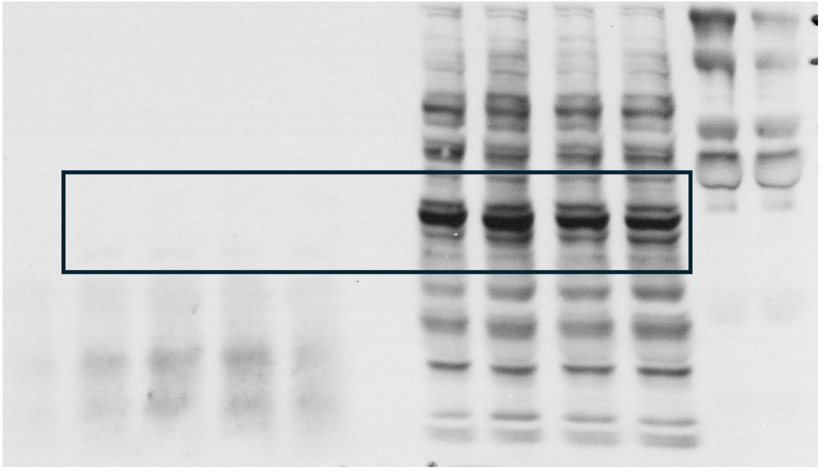

p62

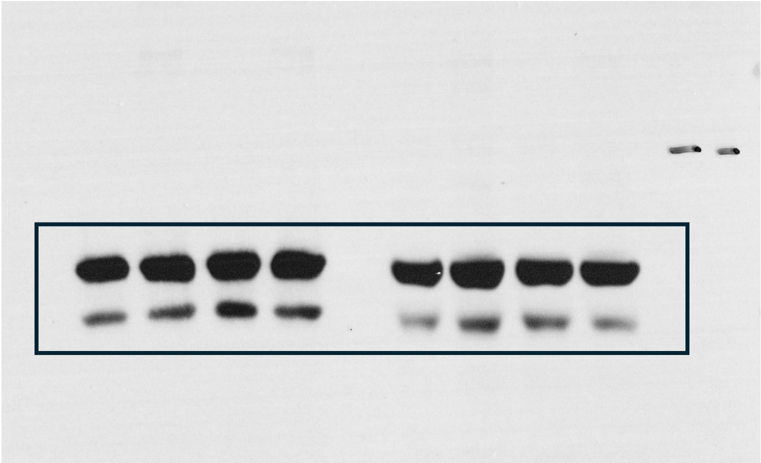

FULL UNEDITED BLOT/GEL FOR SUPPL. FIG. 6M

SOD1

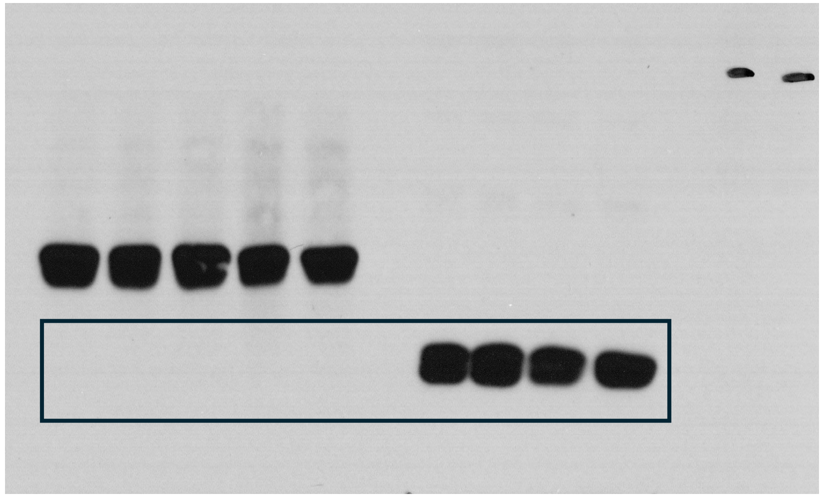

TDP-43

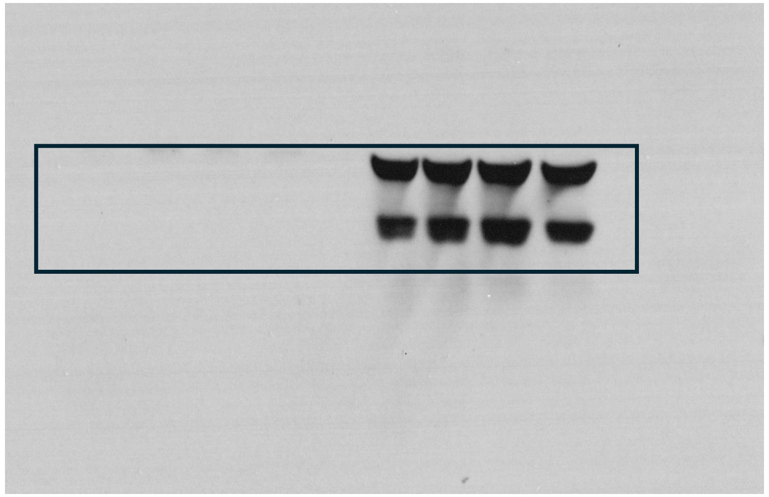

p62

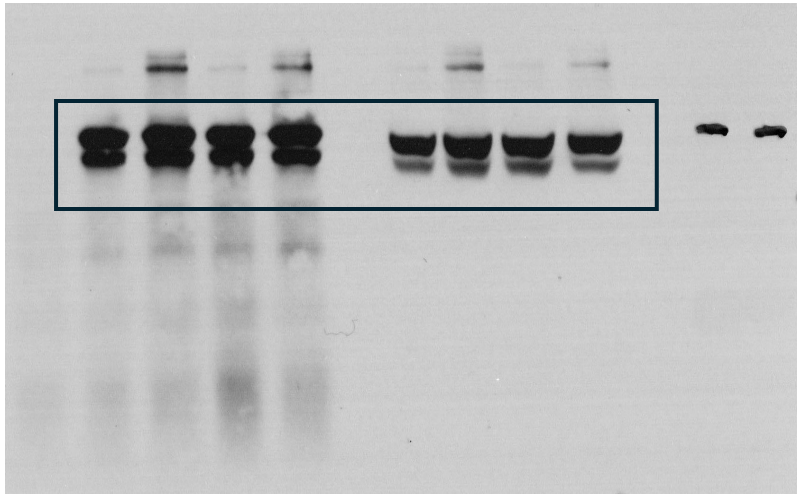

FULL UNEDITED BLOT/GEL FOR SUPPL. FIG. 6N

C9ORF72

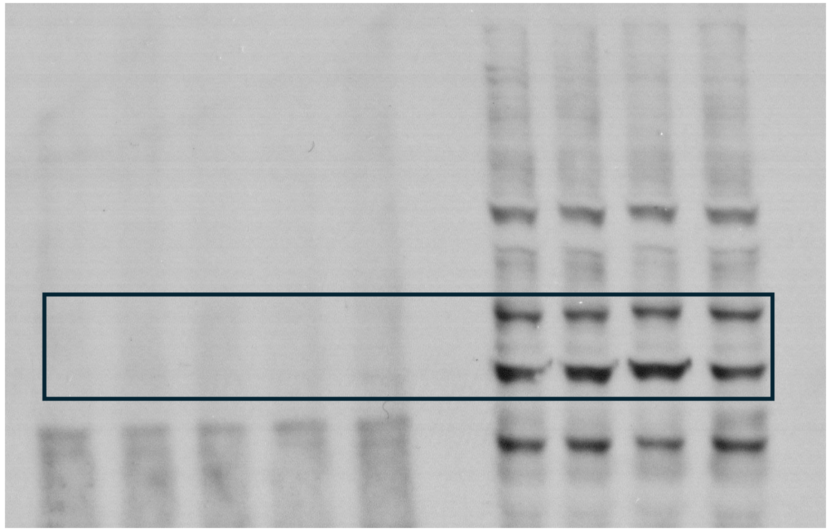

p62

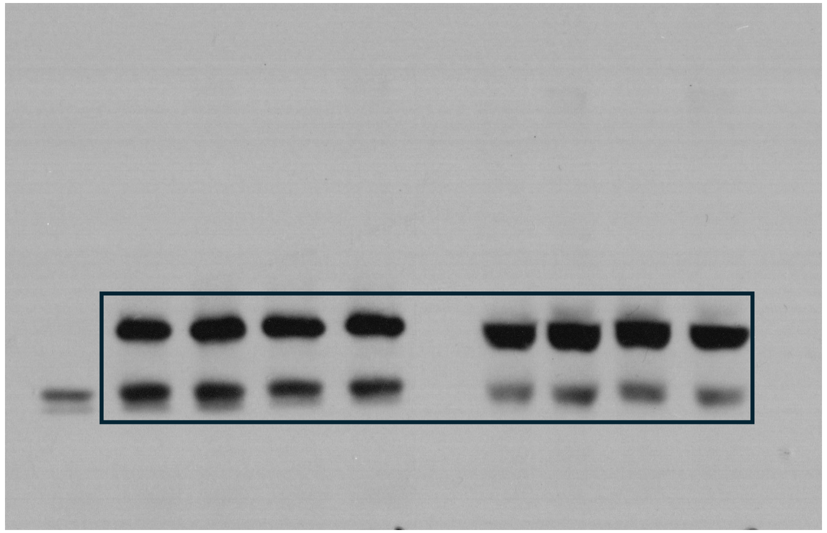

FULL UNEDITED BLOT/GEL FOR SUPPL. FIG. 6O

DEF6

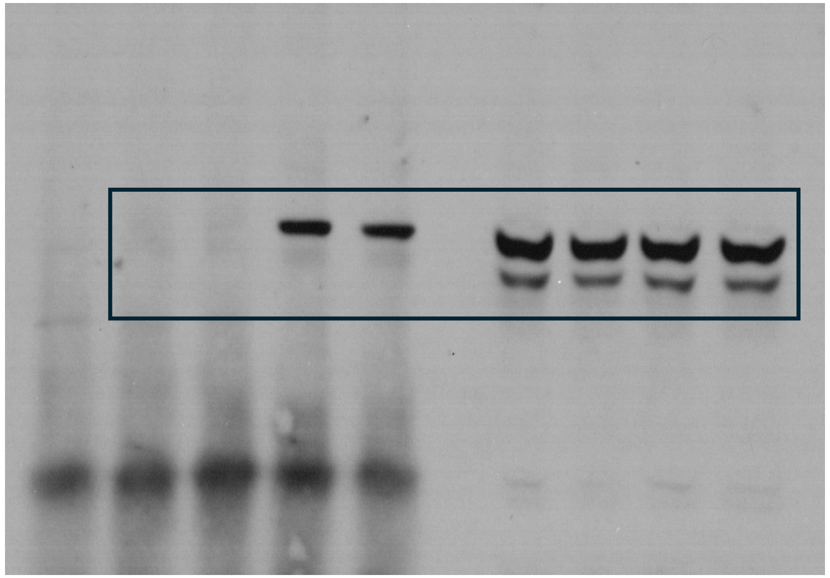

SWAP-70

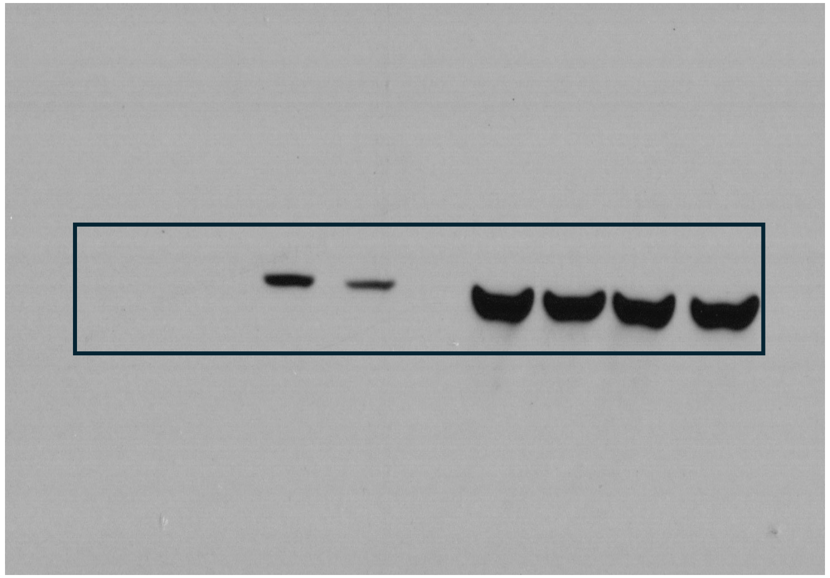

p62

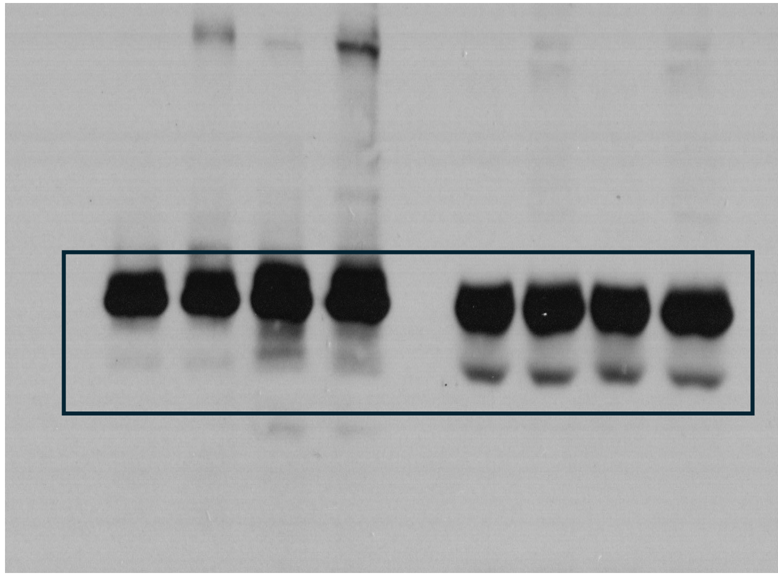

FULL UNEDITED BLOT/GEL FOR SUPPL. FIG. 6P

TDP-43 (p62 IP)

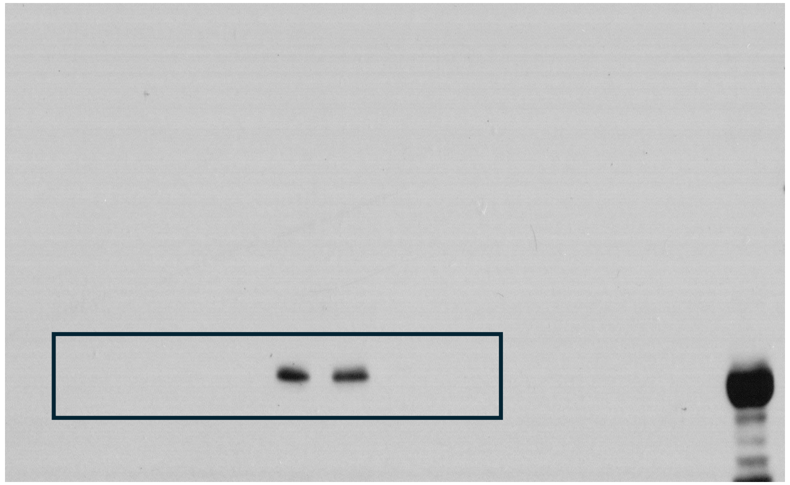

RIPK3 (p62 IP)

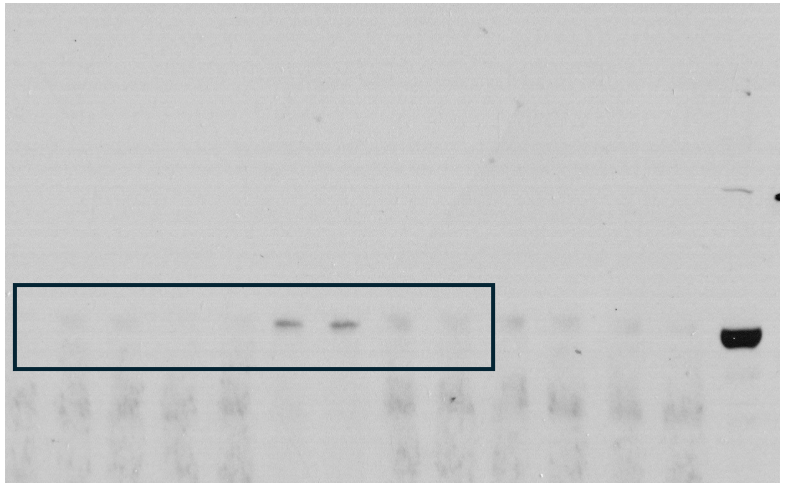

p62

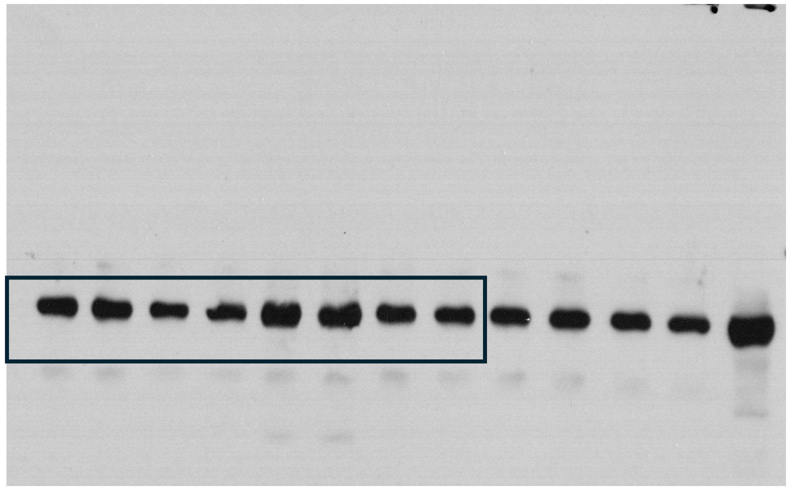

TDP-43 (INPUT)

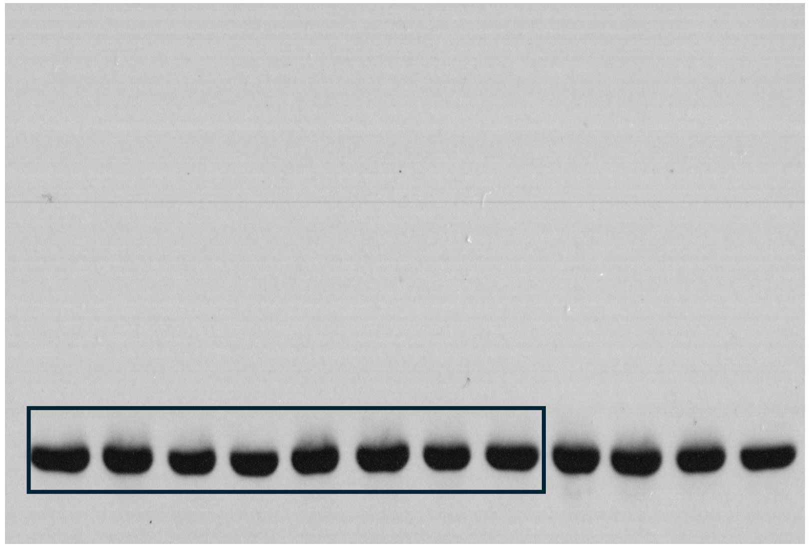

RIPK3 (p62 INPUT)

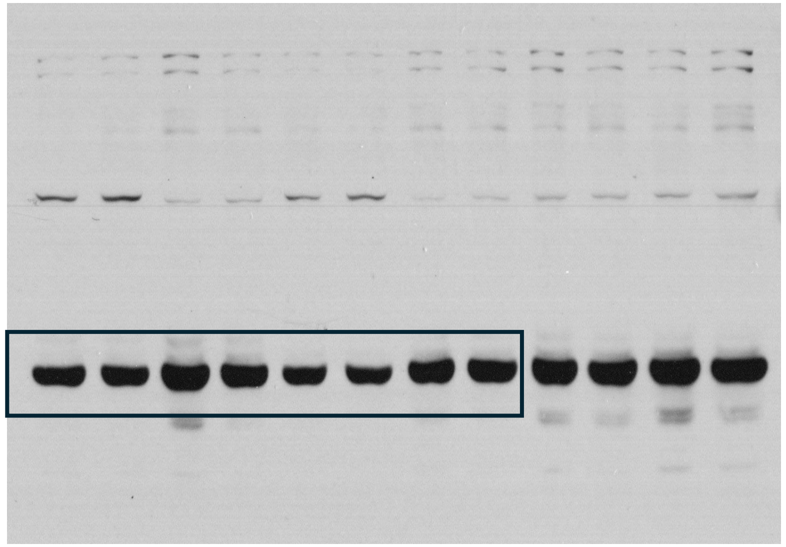

FULL UNEDITED BLOT/GEL FOR FIG. 7A

K63 Ub

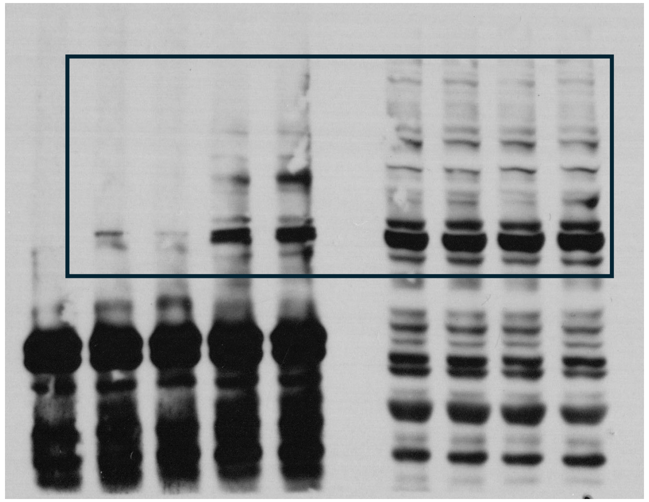

BiP

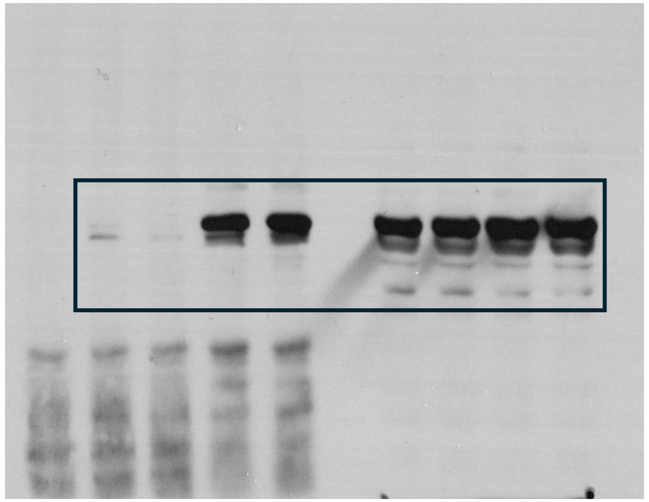

HSP90

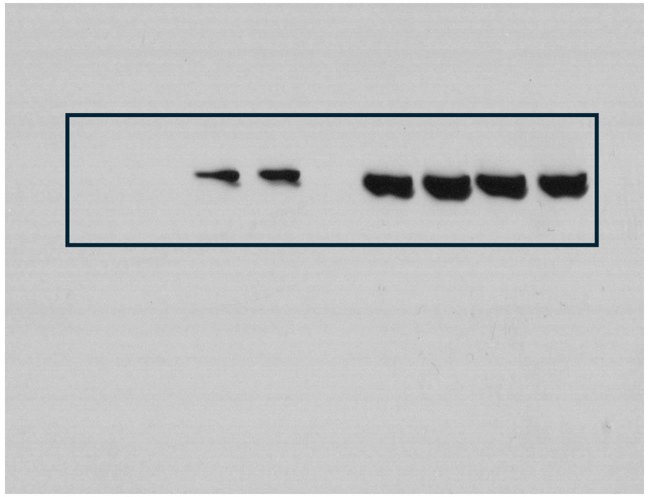

p62

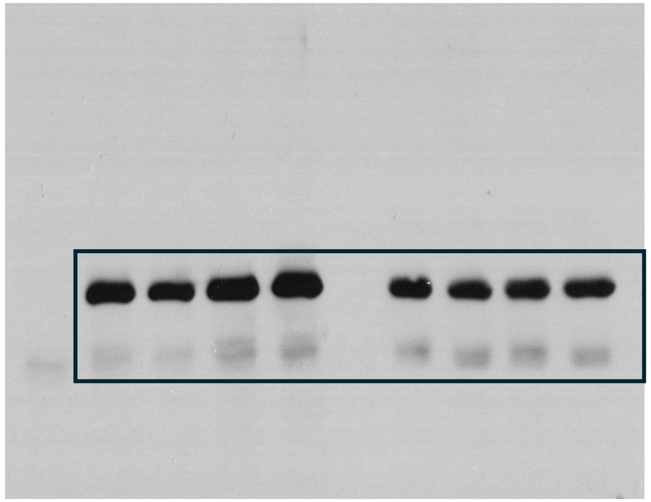

FULL UNEDITED BLOT/GEL FOR FIG. 7B

HRI

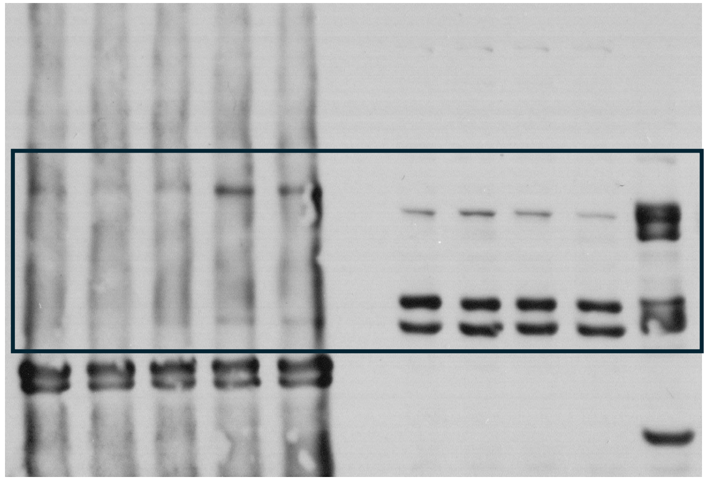

p62

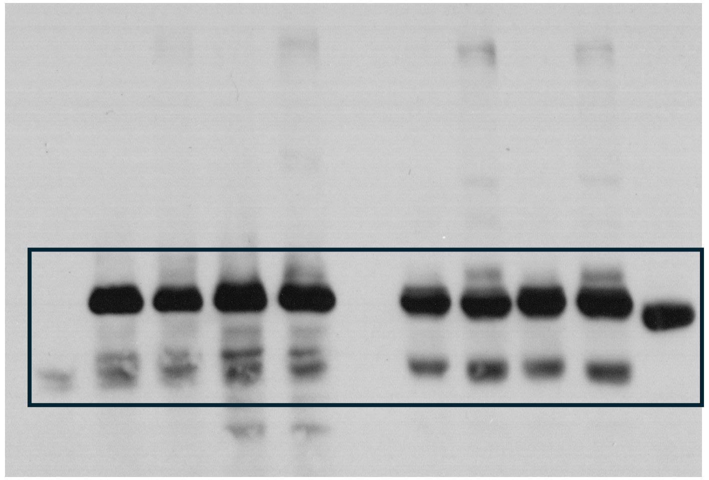

FULL UNEDITED BLOT/GEL FOR FIG. 7C

ATF4

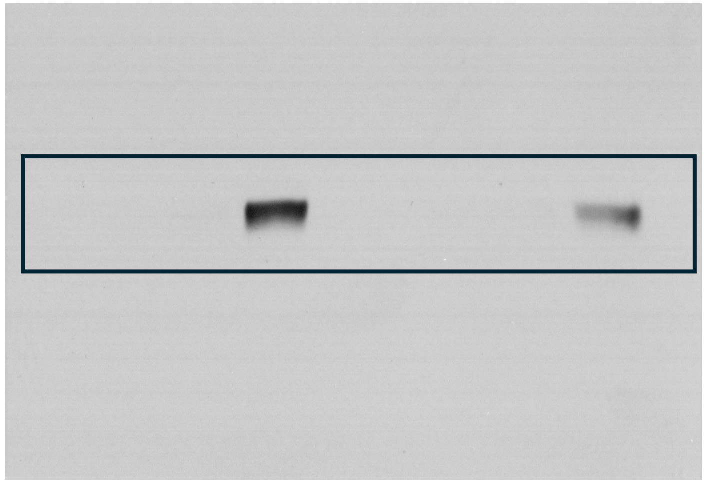

HDAC1

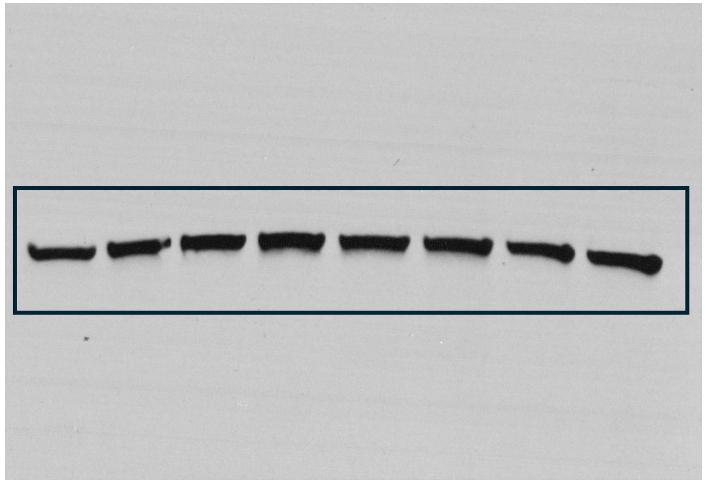

FULL UNEDITED BLOT/GEL FOR FIG. 7D

FLAG

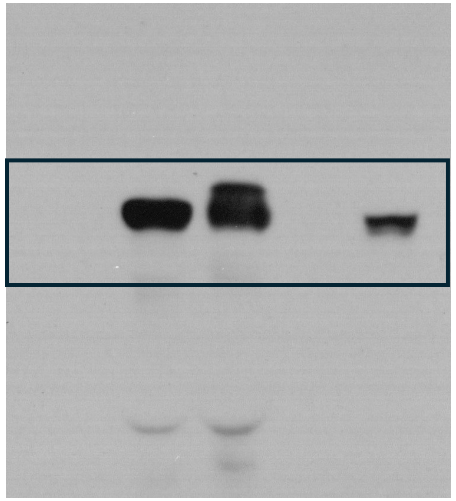

FULL UNEDITED BLOT/GEL FOR FIG. 7E

HSP90

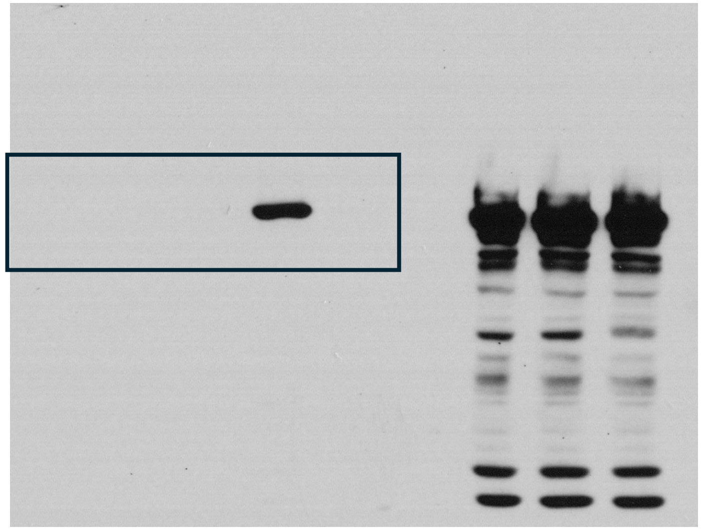

FLAG

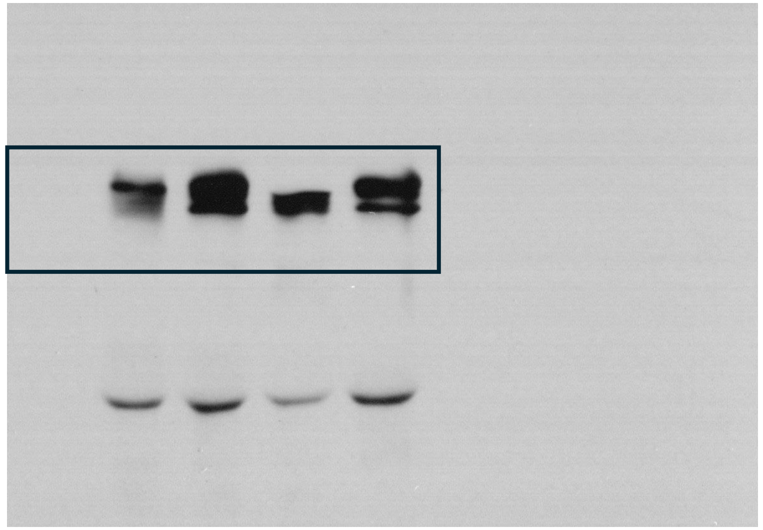

FULL UNEDITED BLOT/GEL FOR FIG. 7G

HSP90

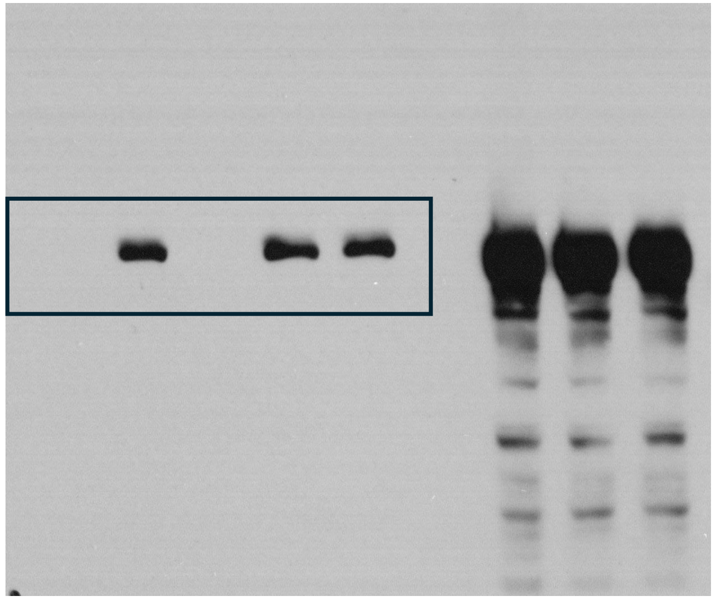

HA

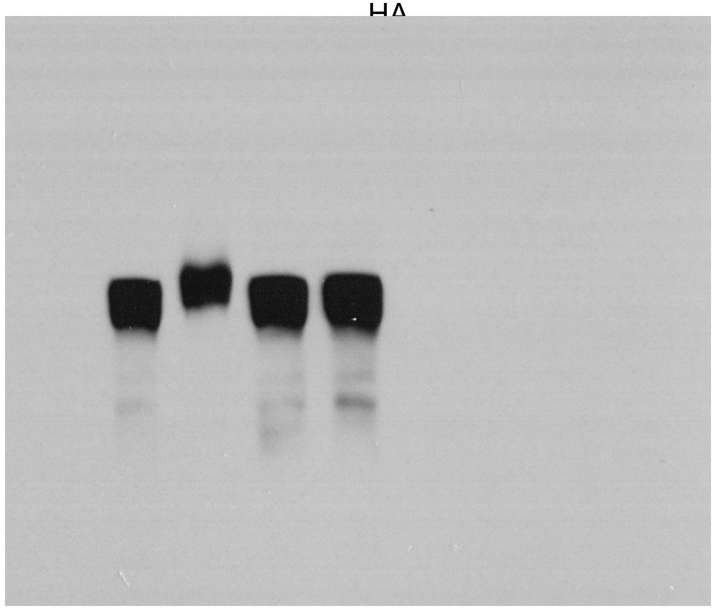

FULL UNEDITED BLOT/GEL FOR FIG. 7H

RAPTOR

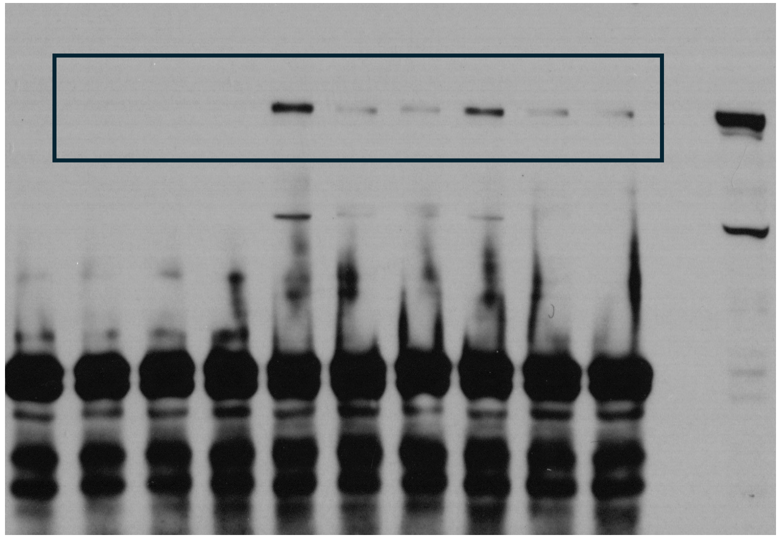

RIPK3

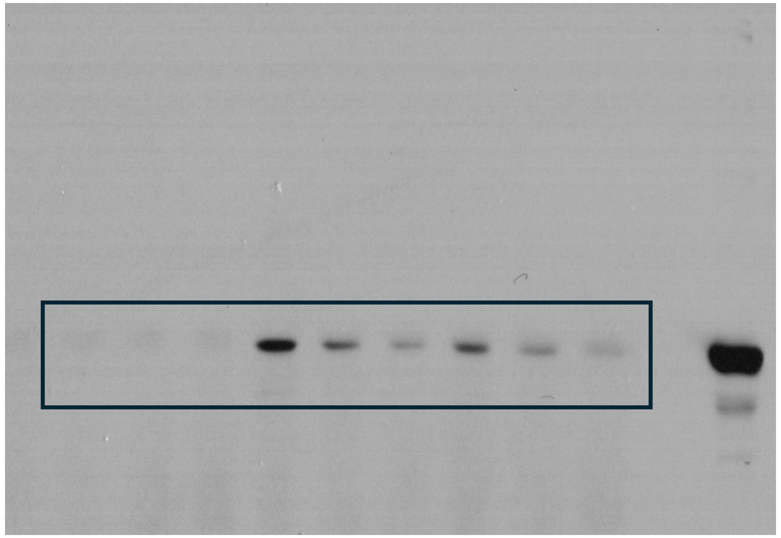

TDP-43

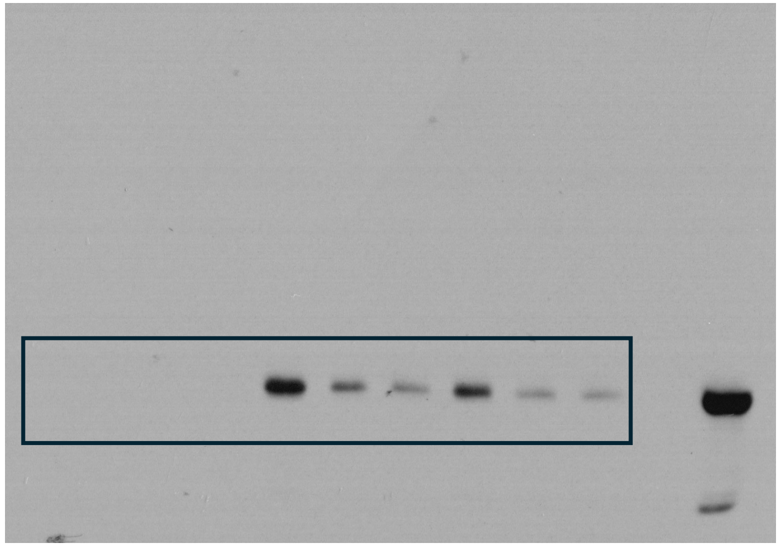

HSP90

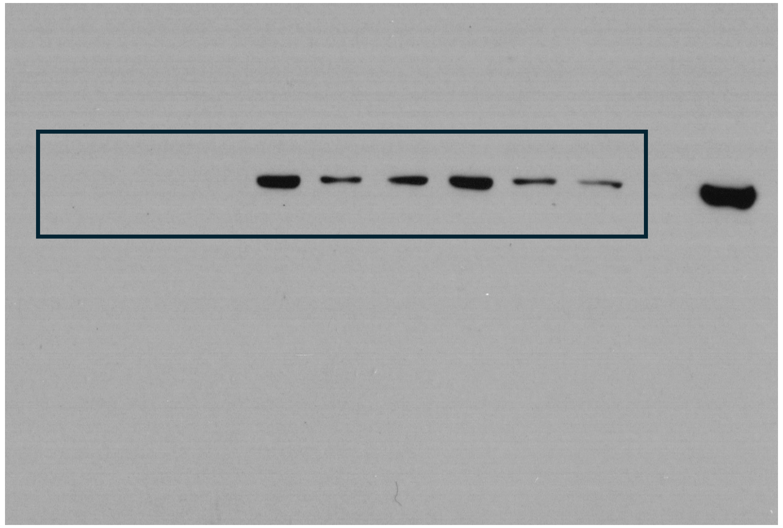

BiP

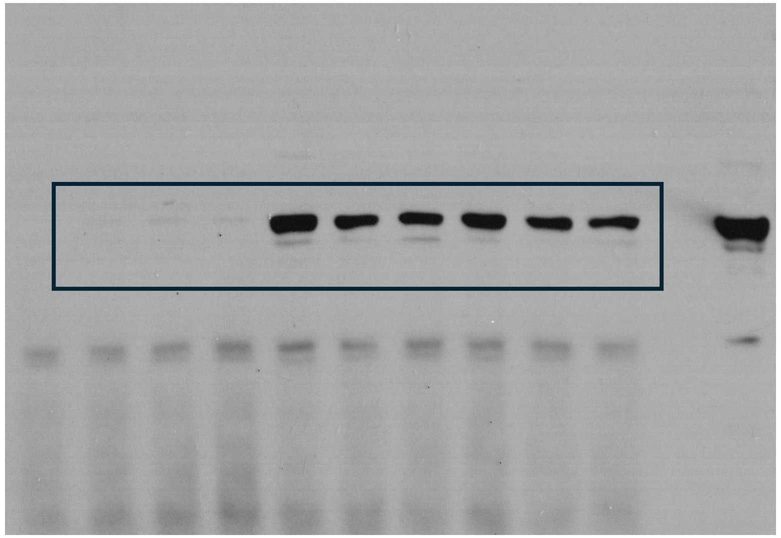

p62

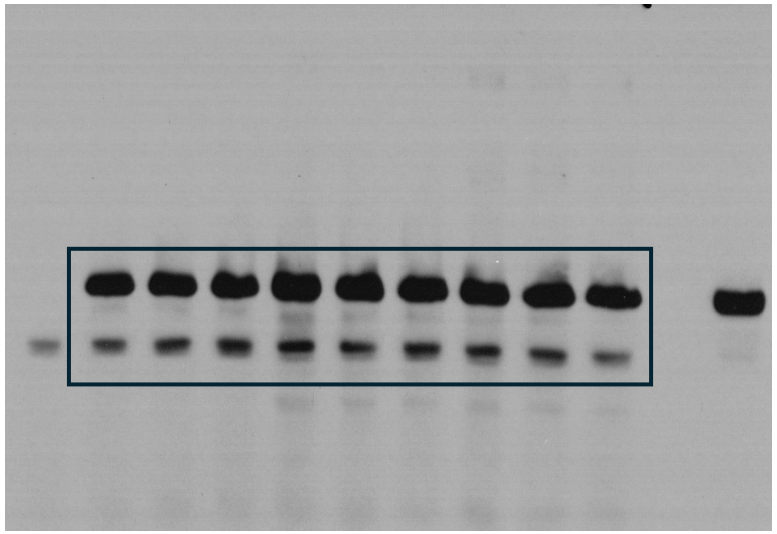

FULL UNEDITED BLOT/GEL FOR FIG. 7I

PHOSPHO-4EBP1

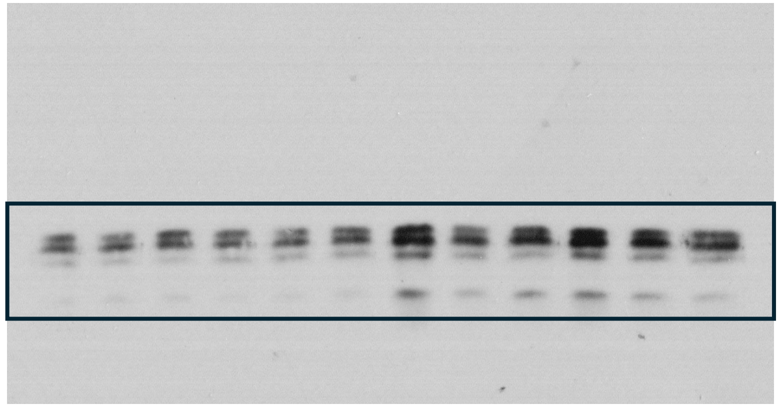

4EBP1

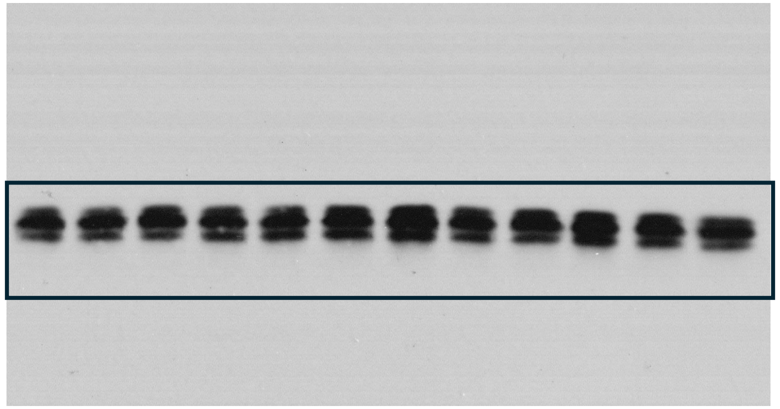

FULL UNEDITED BLOT/GEL FOR SUPPL. FIG. 7A

K63 Ub

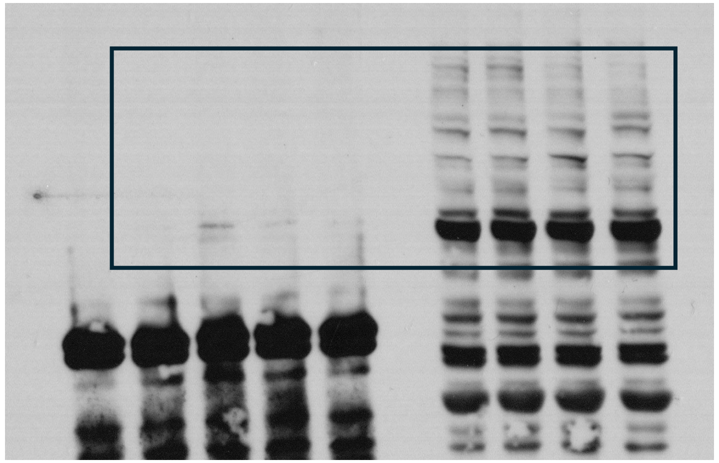

BiP

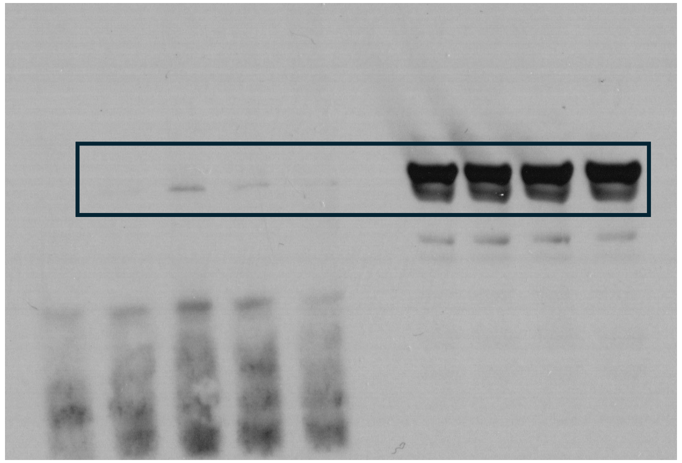

HSP90

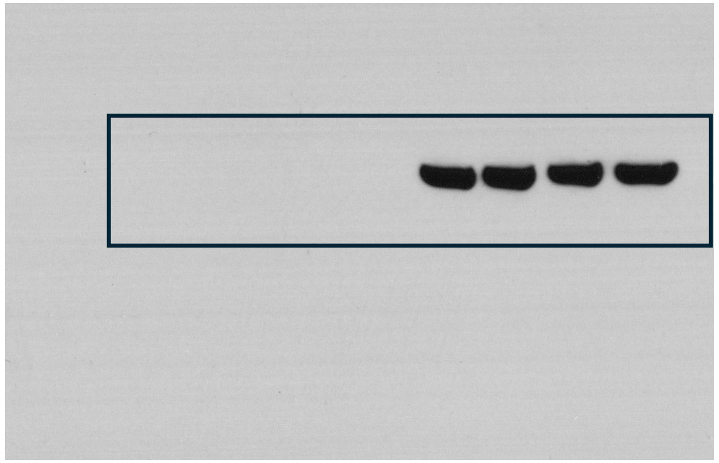

p62

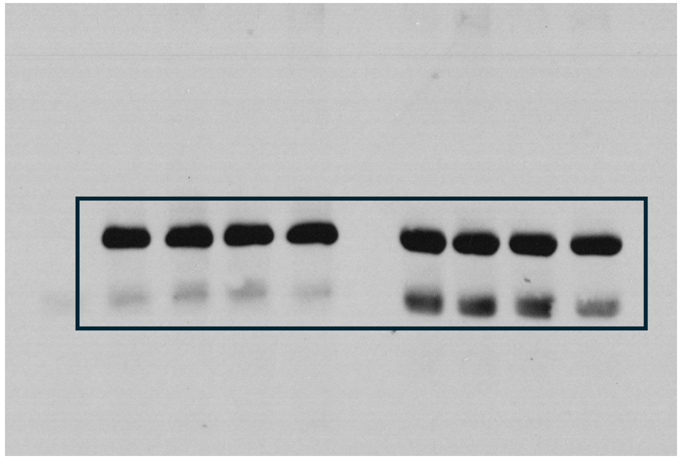

FULL UNEDITED BLOT/GEL FOR SUPPL. FIG. 7B

HRI

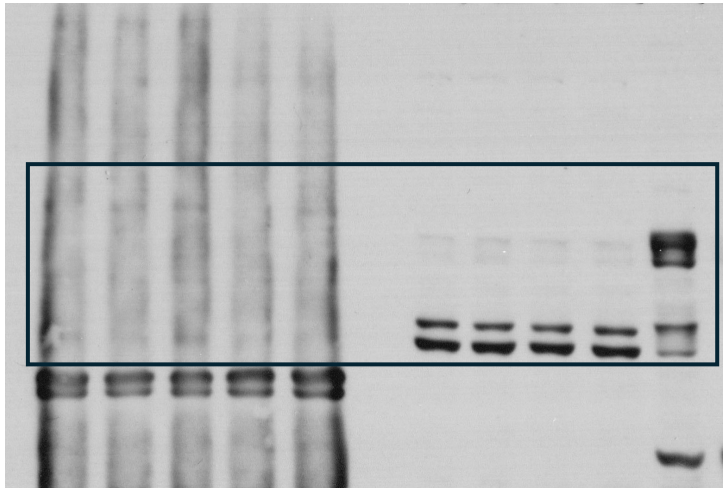

p62

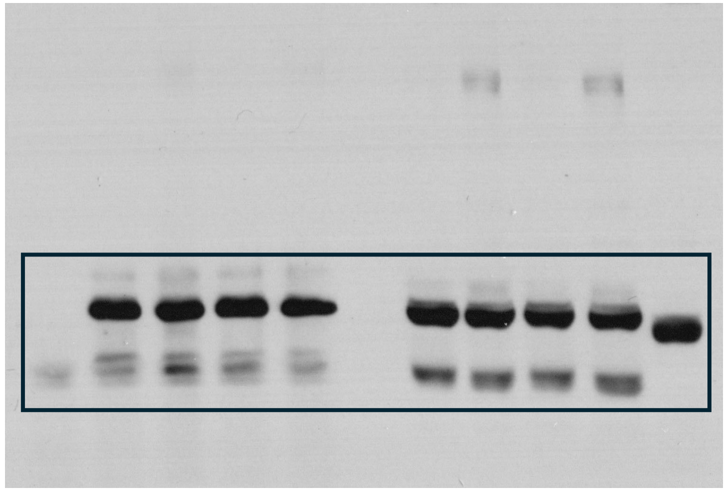

FULL UNEDITED BLOT/GEL FOR SUPPL. FIG. 7C

Phospho-eIF2α

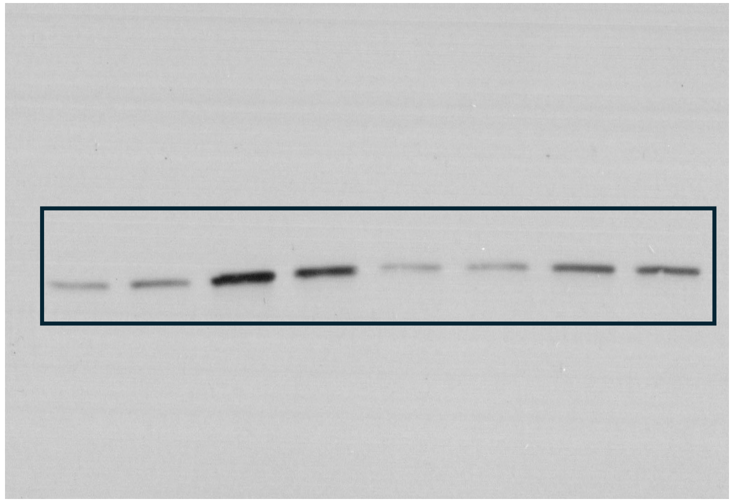

TUBULIN

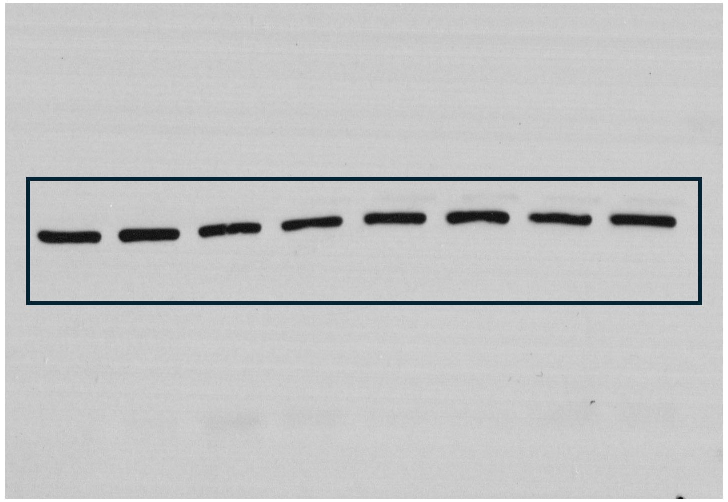

FULL UNEDITED BLOT/GEL FOR SUPPL. FIG. 7D

XBP1

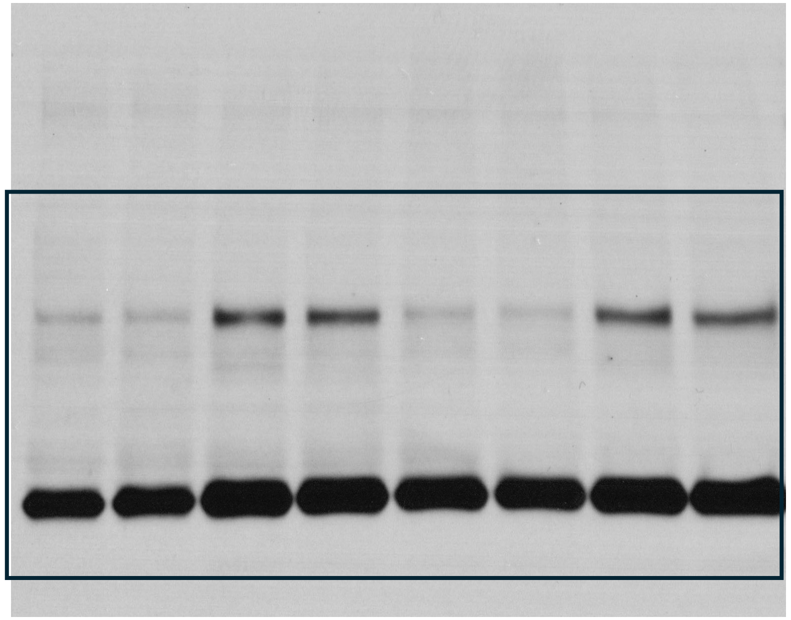

HDAC1

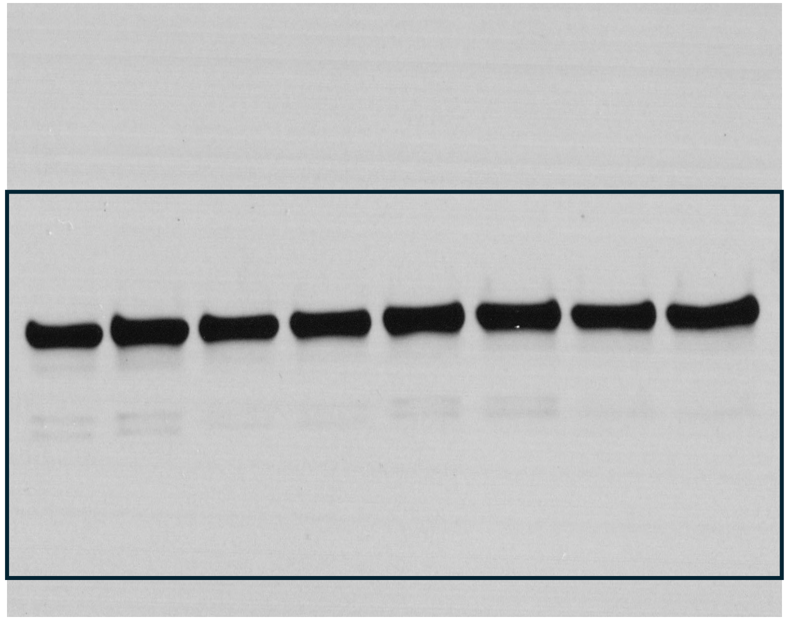

FULL UNEDITED BLOT/GEL FOR SUPPL. FIG. 7E

HA

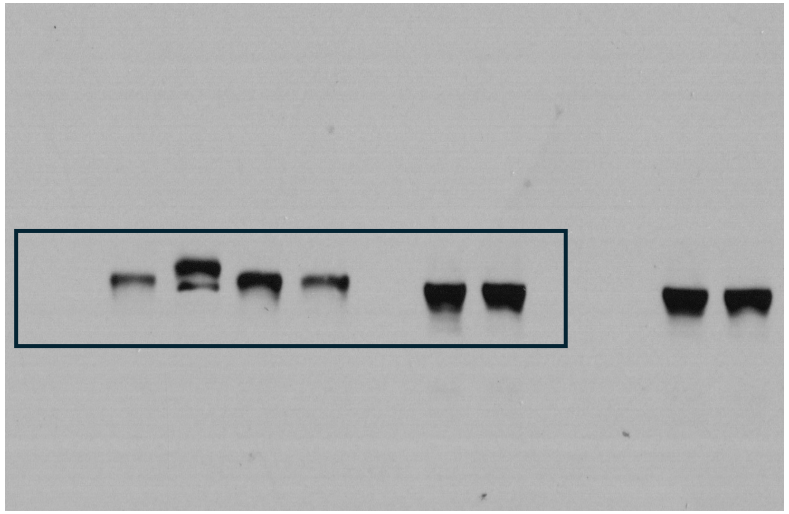

FULL UNEDITED BLOT/GEL FOR SUPPL. FIG. 7F

RAPTOR

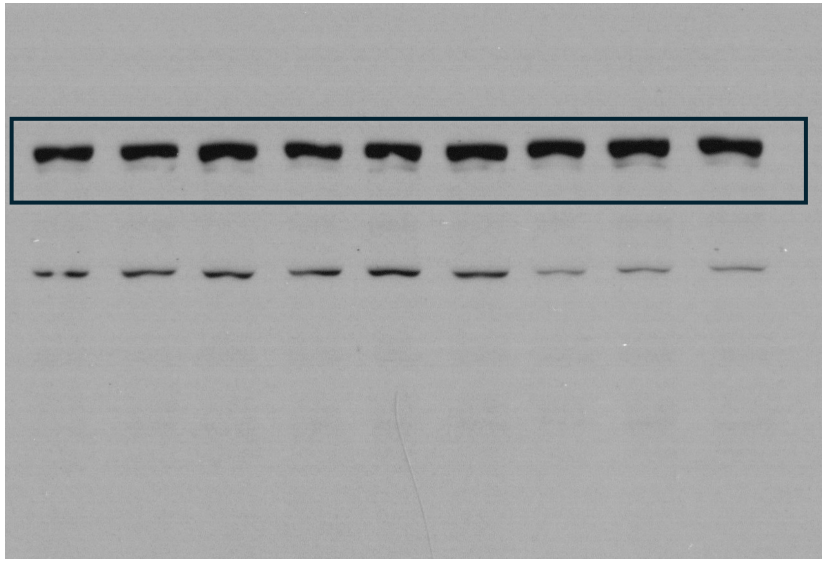

RIPK3

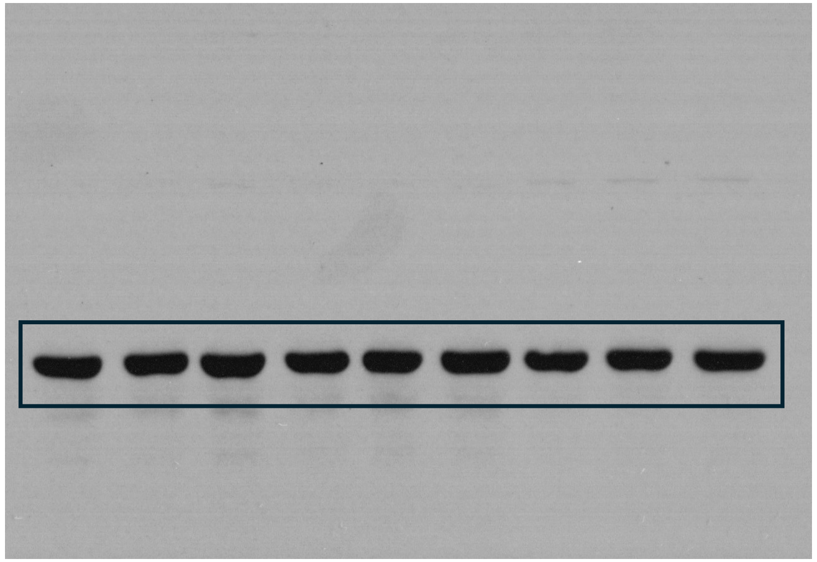

TDP-43

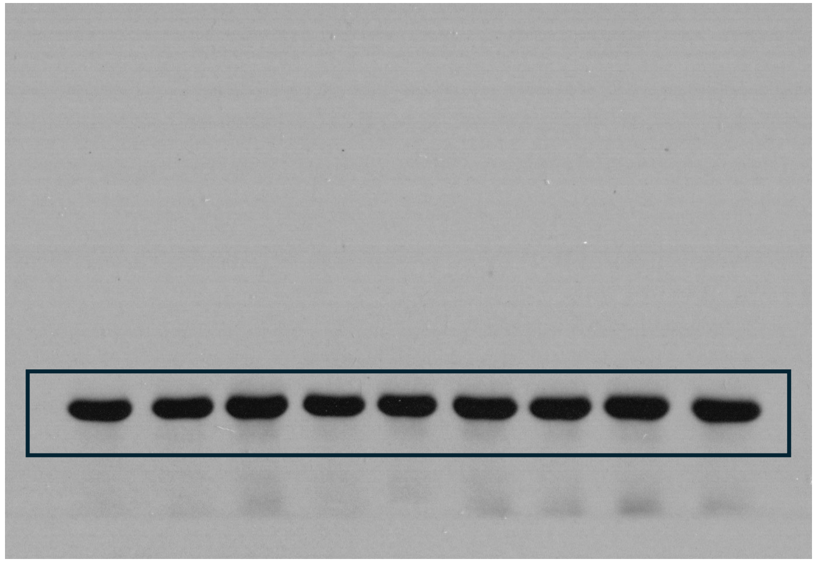

HSP90

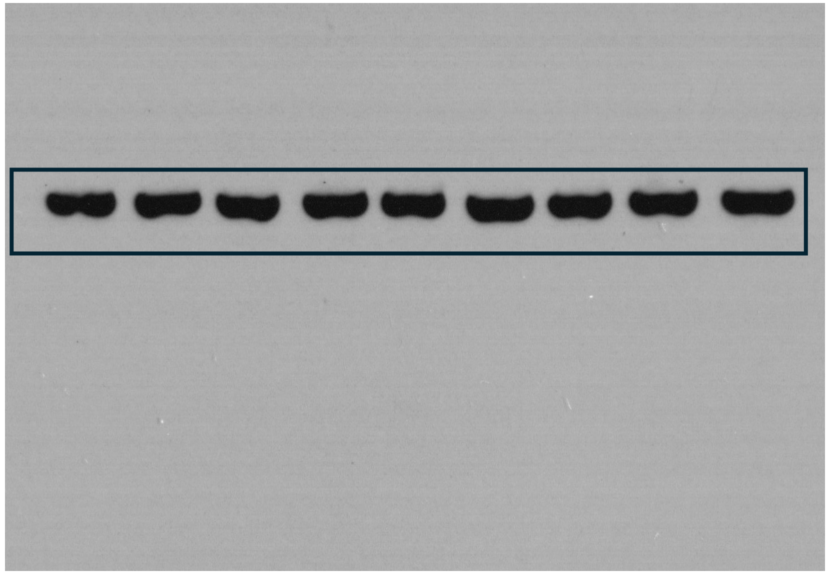

BiP

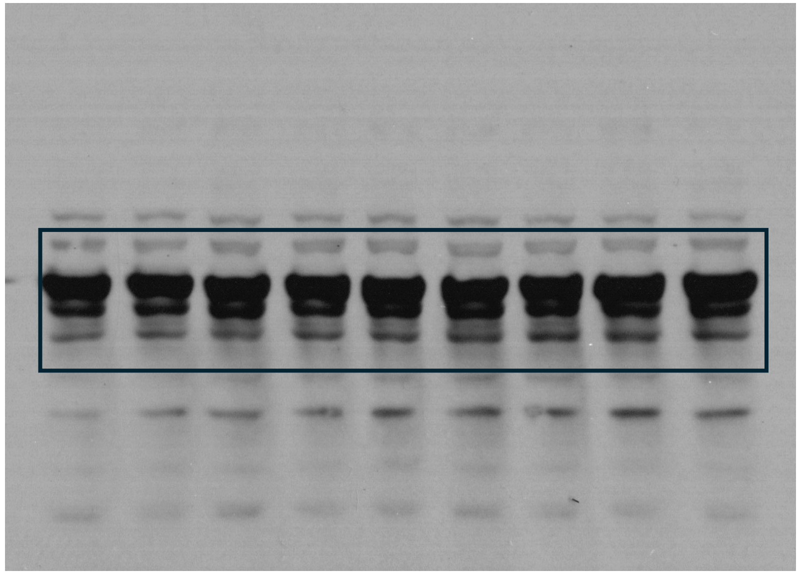

p62

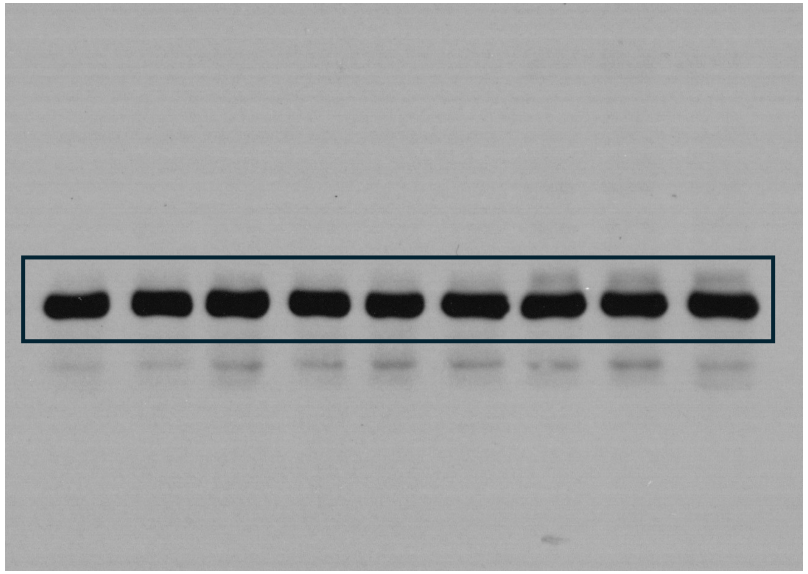

FULL UNEDITED BLOT/GEL FOR SUPPL. FIG. 7G

RAPTOR

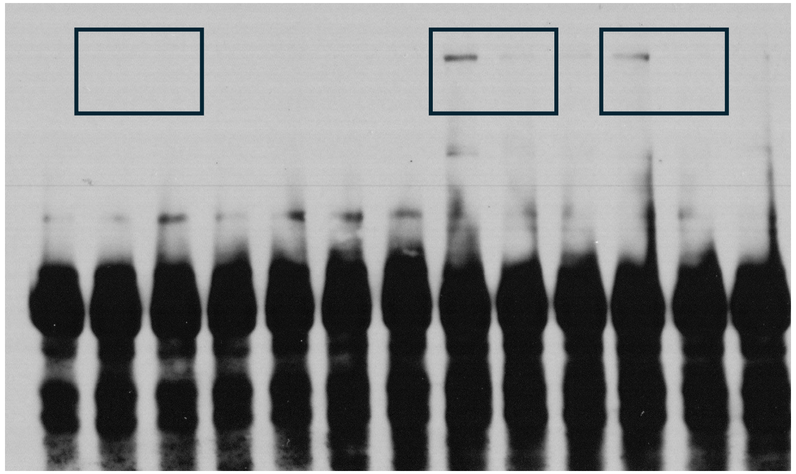

TDP-43

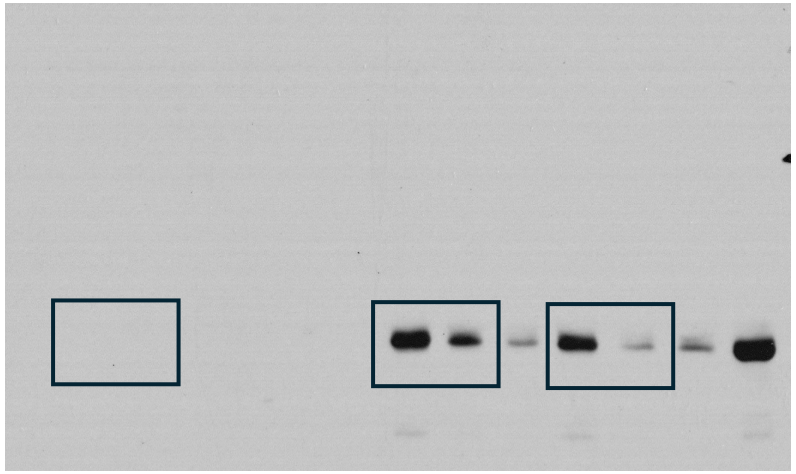

HSP90

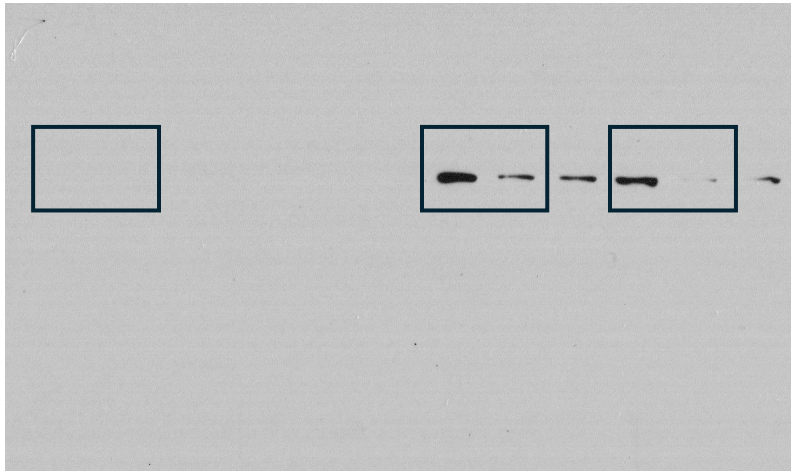

BiP

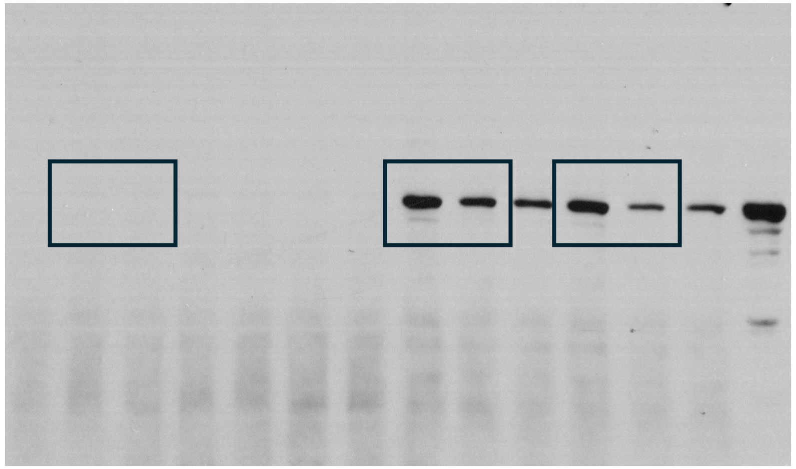

p62

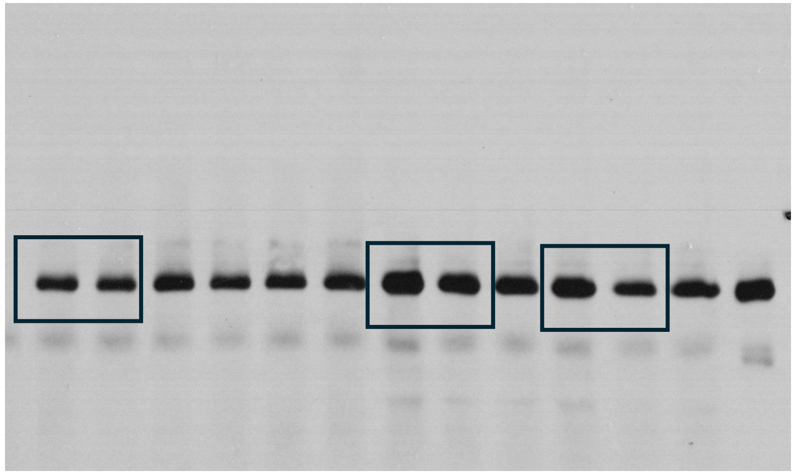

FULL UNEDITED BLOT/GEL FOR SUPPL. FIG. 7G

RAPTOR (INPUT)

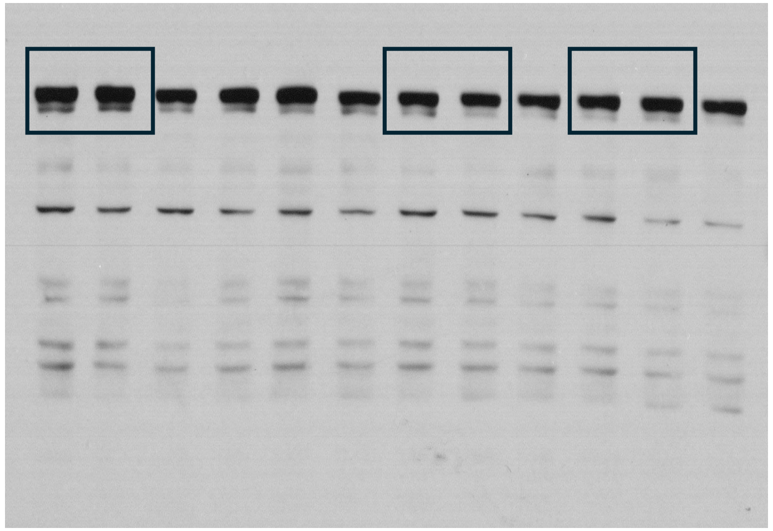

TDP-43 (INPUT)

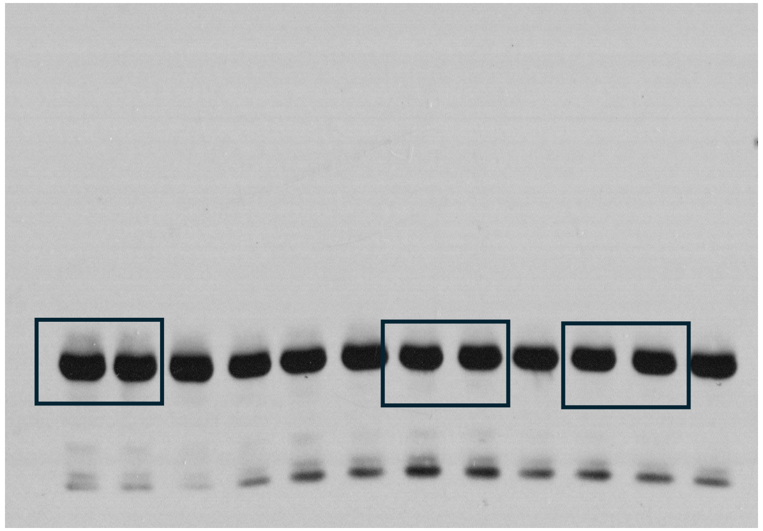

HSP90 (INPUT)

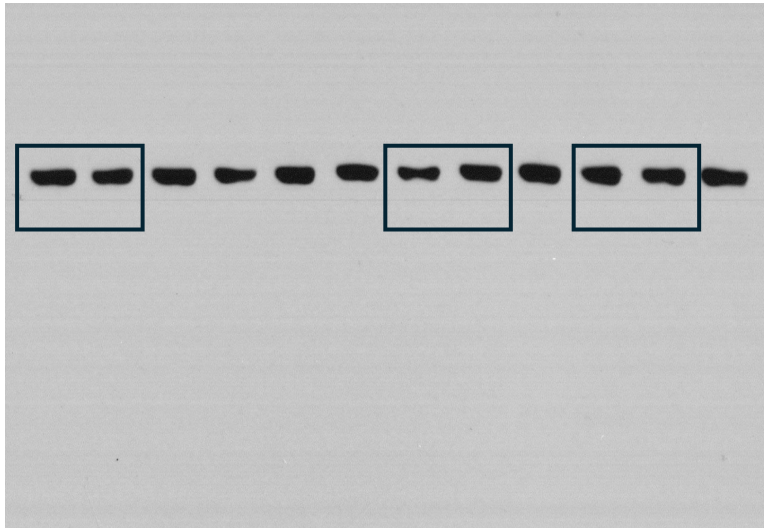

BiP (INPUT)

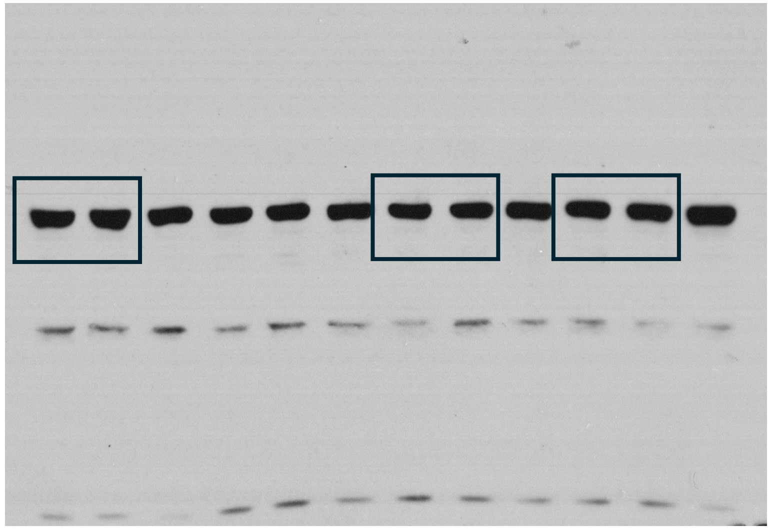

P62 (INPUT)

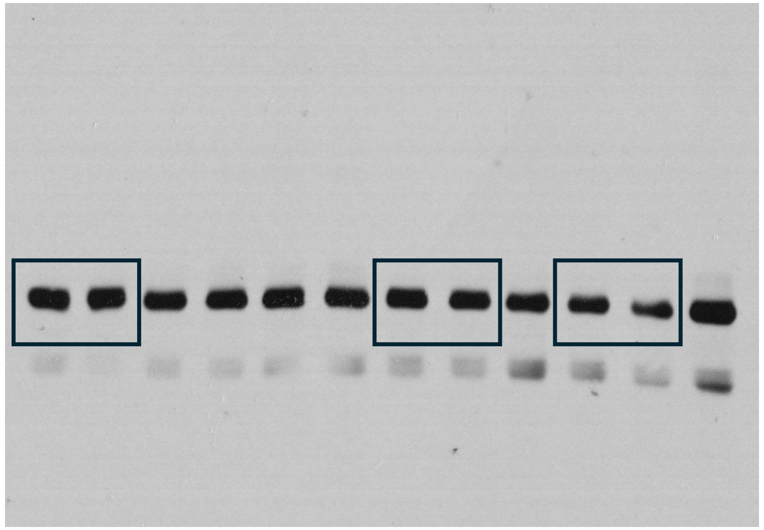

Supplement: Unedited blot and gel images [file jciinsight-10-180507-s040.pdf]
